# Biofortification of field-grown cassava by engineering expression of an iron transporter and ferritin

**DOI:** 10.1038/s41587-018-0002-1

**Published:** 2019-01-28

**Authors:** Narayanan Narayanan, Getu Beyene, Raj Deepika Chauhan, Eliana Gaitán-Solís, Jackson Gehan, Paula Butts, Dimuth Siritunga, Ihuoma Okwuonu, Arthur Woll, Dulce M. Jiménez-Aguilar, Erick Boy, Michael A. Grusak, Paul Anderson, Nigel J. Taylor

**Affiliations:** 1 Donald Danforth Plant Science Center, St. Louis, MO USA; 2 University of Puerto Rico Mayagüez, Puerto Rico, USA; 3 National Root Crops Research Institute, Umudike, Nigeria; 4 Cornell High Energy Synchrotron Source, Cornell University, Ithaca, NY USA; 5 USDA-ARS Children’s Nutrition Research Center, Baylor College of Medicine, Houston, TX USA; 6 Harvest Plus/International Food Policy Research Institute, Washington, DC USA; 7 USDA-ARS Edward T. Schafer Agricultural Research Center, Fargo, ND USA

**Keywords:** Biotechnology, Plant biotechnology, Field trials, Molecular engineering in plants, Plant sciences

## Abstract

Less than 10% of the estimated average requirement (EAR) for iron and zinc is provided by consumption of storage roots of the staple crop cassava (*Manihot esculenta* Crantz) in West African human populations. We used genetic engineering to improve mineral micronutrient concentrations in cassava. Overexpression of the *Arabidopsis thaliana* vacuolar iron transporter VIT1 in cassava accumulated three- to seven-times-higher levels of iron in transgenic storage roots than nontransgenic controls in confined field trials in Puerto Rico. Plants engineered to coexpress a mutated *A. thaliana* iron transporter (IRT1) and *A. thaliana* ferritin (FER1) accumulated iron levels 7–18 times higher and zinc levels 3–10 times higher than those in nontransgenic controls in the field. Growth parameters and storage-root yields were unaffected by transgenic fortification in our field data. Measures of retention and bioaccessibility of iron and zinc in processed transgenic cassava indicated that *IRT1* *+* *FER1* plants could provide 40–50% of the EAR for iron and 60–70% of the EAR for zinc in 1- to 6-year-old children and nonlactating, nonpregnant West African women.

## Abstract (web summary)

Cassava, a staple crop consumed by 800 million people, is enriched in iron and zinc through genetic biofortification.

## Main

Micronutrient deficiency poses a threat to human health worldwide. An estimated 161 million children under 5 years of age are stunted, partly because of hidden hunger, which occurs when foodstuffs lack essential vitamins and minerals^1^. In Nigeria, 75% of preschool children and 67% of pregnant women are anemic^2^, and 20% of children under 5 years of age have zinc deficiency^3^. Iron-deficiency anemia affects the immune system, stunts growth and impairs cognitive development in children^4^. Zinc deficiency causes increased risk of death from diarrhea, stunting and hindered cognitive development^4^. Biofortification of staple food crops through biotechnology is one of several strategies for improving essential micronutrients in foods for at-risk populations^5^. Approximately 800 million people worldwide consume the tropical root crop cassava, and one-third of the sub-Saharan African population relies on cassava for more than 50% of their caloric intake^6^. Although cassava is an excellent source of carbohydrate, the storage roots provide inadequate levels of bioavailable iron and zinc^5,7^. A lack of genetic variation for mineral traits within the cassava germplasm^8^ makes breeding new lines with improved mineral content challenging. A genetic engineering strategy has been undertaken to increase iron and zinc concentrations in cassava storage roots^9^.

Genetic engineering has been successfully applied to increase mineral concentrations in cereal crops, including rice. Iron concentrations in polished rice grains have been increased by overexpressing the soybean or rice storage protein ferritin^10^ and coexpressing *Arabidopsis* nicotianamine synthase, common bean ferritin and *Aspergillus* phytase^11^. Overexpression of *AtIRT1*, *AtNAS1* and bean *FERRITIN* in rice resulted in 3.8-fold higher iron and 1.8-fold higher zinc concentrations than in the wild-type control^12^. Recently, overexpression of the soybean ferritin SFER-H1 and rice nicotianamine synthase OsNAS2 has achieved dietary targets for both iron and zinc nutrition in rice grains^13^. Despite successes in rice, reports of engineering-improved mineral biofortification in dicotyledonous plants are rare and are mainly restricted to the model plant *A. thaliana*. Nongrass plants use a reduction-based mechanism for iron acquisition^14^ mediated by the plasma-membrane-bound oxidoreductase FRO2, and the ZIP-family transporter IRT1. Transgenic overexpression of the algal iron assimilatory protein FEA1 has resulted in a threefold increase in storage-root iron concentrations in greenhouse-grown cassava^15^, but these promising results were not maintained in field trials. Increased zinc concentrations in storage roots have been achieved by overexpression of the *A. thaliana* (At) zinc transporters AtZIP1 and AtMTP1, but shoot development in transgenic plants is impaired^16^.

We previously found that overexpression of the *A. thaliana* vacuolar iron transporter VIT1 in cassava results in a three- to four-times increase in iron concentration in storage roots compared with the concentrations in nontransgenic controls under greenhouse conditions^17^. Here, we report that coexpression of a mutant *A. thaliana* iron transporter (IRT1)^18^ and ferritin (FER1) generates transgenic cassava plants that accumulate iron and zinc in storage roots to substantial levels in the human diet. Data from field-grown *VIT1* and *IRT1* *+* *FER1* transgenic lines in Puerto Rico field trials (2014–2017) indicate that both technologies result in cassava storage roots and foodstuffs with elevated iron and zinc levels that may beneficially affect the nutritional status of consumers.

We designed *IRT1* and *FER1* expression cassettes to improve mineral uptake, by placing At*IRT1* (ref. ^18^) under control of the A14 promoter, and to store iron in plastids, by expressing At*FER1* (ref. ^19^) under control of the patatin type 1 promoter (Supplementary Fig. 1a). Transgenic cassava plants of cultivar TME 204 coexpressing *IRT1* and *FER1* mRNA were established in the greenhouse. The shoot and storage-root growth phenotypes were similar for all transgenic and nontransgenic control lines during 16 weeks of growth (Supplementary Fig. 1b–g). The presence of *IRT1* and *FER1* transgenes in the leaves of 4-week-old plants was confirmed by PCR (Supplementary Fig. 2a). Southern blot analyses verified the integration of the *IRT1* and *FER1* transgenes at one or two copies of the transfer DNA (T-DNA; Supplementary Fig. 2b), and mRNA expression of *IRT1* and *FER1* was confirmed by RT–qPCR in leaves, fibrous roots and storage roots of transgenic plants (Supplementary Fig. 2c–h). Inductively coupled plasma optical emission spectroscopy (ICP–OES) analysis revealed that the storage roots of *IRT1* *+* *FER1* transgenic plants had five- to six-times-higher iron and zinc concentrations than the storage roots of nontransgenic controls (Supplementary Fig. 1j,k). The maximum iron accumulation reached 55 ± 13 µg/g dry weight (DW; mean ± s.d.) compared with 10 ± 2 µg/g DW for storage roots of nontransgenic plants (Supplementary Fig. 1j), and 26 ± 12 µg/g DW zinc compared with 5 ± 1 µg/g DW zinc in the nontransgenic controls (Supplementary Fig. 1k). *IRT1* *+* *FER1* transgenic plants had leaf iron concentrations two to three times higher than those of nontransgenic controls (Supplementary Fig. 1h), but no increase in zinc concentration was observed in foliar tissues (Supplementary Fig. 1i). The total iron and zinc content was determined in leaves, petioles, stem, fibrous roots and storage-root peels to assess whether the elevated mineral levels in the storage-root parenchyma resulted from depletion in other organs. When assessed as whole plants, *IRT1* *+* *FER1* transgenic lines, compared with nontransgenic controls, showed significantly higher (*P* ≤ 0.01) total iron and zinc content, by up to five and two times, respectively (Supplementary Fig. 3a,b). The maximum total iron accumulation reached 7,059 ± 204 µg iron in transgenic line 8023-14 compared with 1,311 ± 38 µg iron in nontransgenic plants. The iron content increased in all organs except fibrous roots, and the greatest increase occurred in storage roots (Supplementary Fig. 3a). The zinc content was increased in storage roots, root peels and stems, but not in leaves, petioles or fibrous roots of *IRT1* *+* *FER1* plants (Supplementary Fig. 3b).

**Fig. 1:**
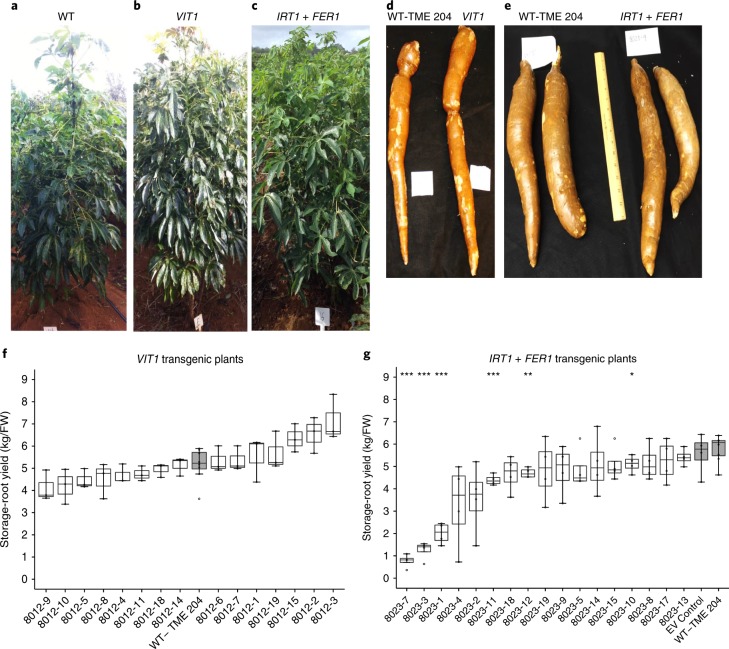
Agronomic yield of nontransgenic, *VIT1* and *IRT1* *+* *FER1* transgenic cassava plants. **a**–**e**, Shoot phenotypes of 12-month-old nontransgenic control TME 204 (**a**), *VIT1* (**b**) and *IRT1* *+* *FER1* (**c**) plants. Waxed storage roots of nontransgenic control and transgenic *VIT1* (**d**) and *IRT1* *+* *FER1* (**e**) plants. **f**,**g**, Storage-root yields for *VIT1* (**f**) and *IRT1* *+* *FER1* (**g**) transgenic plants, along with nontransgenic control. Box-and-whisker plots were constructed with the R package ggplot2. The upper whisker extends from the hinge to the largest value, no further than 1.5× the interquartile range (IQR, distance between the first and third quartiles) from the hinge. The lower whisker extends from the hinge to the smallest value, at most 1.5× the IQR of the hinge. Data beyond the ends of the whiskers are considered outlying points and are plotted individually. For *VIT1*, *n* = 9 biologically independent plants (3 plants/replicate); for *IRT1* *+* *FER1, n* = 4 biologically independent plants. Statistical tests were performed with two-sided Student’s *t* test, relative to nontransgenic control. *P ≤ 0.05; **P ≤ 0.01; ***P ≤ 0.001. WT, wild-type plants; EV control, empty-vector control plants.

*VIT1* and *IRT1* *+* *FER1* transgenic lines were evaluated in confined field trials at Isabela field station, University of Puerto Rico (Supplementary Fig. 4). Over a 12-month trial period, no significant differences were found between *VIT1* transgenic plants and nontransgenic controls for root or shoot biomass, storage-root dry-matter content, number of roots, harvest index or linamarin concentration (Fig. 1 and Supplementary Fig. 5). For *IRT1* *+* *FER1* transgenic plants, 12 of the 17 lines tested in the field generated storage-root yields (Fig. 1g) and shoot yields (Supplementary Fig. 6a) comparable to those of nontransgenic controls, and there were no significant differences in the number of storage roots, harvest index, dry matter or total linamarin concentration (Supplementary Fig. 6).

**Fig. 2:**
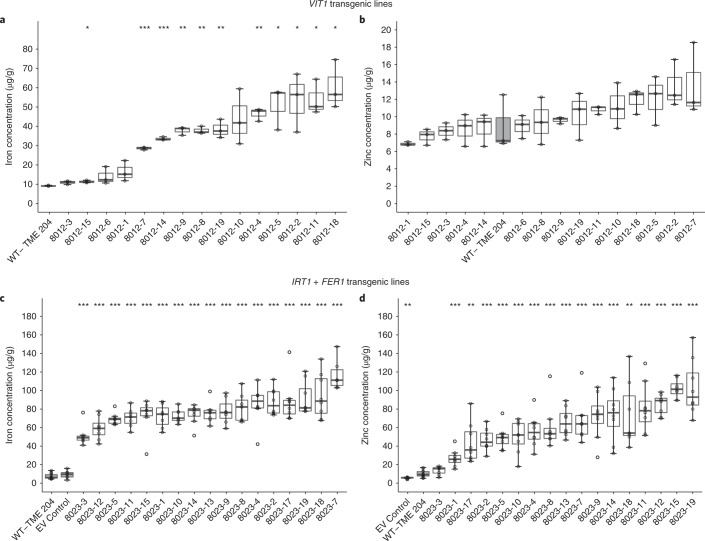
Storage-root mineral concentrations of *VIT1* and *IRT1* *+* *FER1* transgenic cassava at harvest 12 months after planting under field conditions. **a**,**b**, Iron (**a**) and zinc (**b**) concentrations in storage roots of *VIT1* transgenic cassava plants. **c**,**d**, Iron (**c**) and zinc (**d**) concentrations instorage roots of *IRT1* *+* *FER1* transgenic cassava plants. For *VIT1*, *n* = 9 biologically independent plants (3 plants/replicate); for *IRT1* *+* *FER1, n* = 4 biologically independent plants. Box-and-whisker plots were constructed with the R package ggplot2. The upper whisker extends from the hinge to the largest value, no further than 1.5× the IQR from the hinge. The lower whisker extends from the hinge to the smallest value, at most 1.5× the IQR of the hinge. Data beyond the ends of the whiskers are considered outlying points and are plotted individually. Statistical tests were performed with two-sided Student’s *t* test, relative to nontransgenic control. *P ≤ 0.05; **P ≤ 0.01; ***P ≤ 0.001.

Mineral accumulation was determined in the storage roots of field-grown plants. We previously reported that cassava plants overexpressing AtVIT1 accumulated up to 48 µg/g DW iron in storage roots under greenhouse conditions, a level three to four times higher than that in nontransgenic controls^17^. Similar results were seen in field-grown materials, in which 10 of the 15 *VIT1* lines had iron concentrations three to seven times higher, reaching a maximum of 60 ± 7 µg/g DW (Fig. 2a). No elevation in zinc concentration was observed in the storage roots produced by *VIT1* transgenic plants (Fig. 2b). All 17 *IRT1* *+* *FER1* transgenic lines grown in the field accumulated significantly elevated levels of iron (*P* ≤ 0.001) in their storage roots, which reached 130 ± 39 µg/g DW, an 18-fold increase over the 7.2 ± 3 µg/g DW in nontransgenic controls (Fig. 2c). Fifteen *IRT1* *+* *FER1* transgenic lines also had significantly elevated levels of storage-root zinc (*P* ≤ *0.001*), and line 8023-19 reached a maximum of 103 ± 30 µg/g DW, a level ten times higher than that in nontransgenic controls (Fig. 2d). The elevated iron in these storage roots was positively correlated with an elevation of zinc concentration (*r* *=* 0.64; Fig. 2c,d). *VIT1* transgenic plants showed a minor but significant elevation in copper, manganese and nickel concentrations (Supplementary Fig. 7), but the cadmium concentrations were below the detection limits. Likewise, *IRT1* *+* *FER1* plants had elevated copper and manganese concentrations (Supplementary Fig. 8), but the nickel and cadmium concentrations were below the detection limits (data not shown).

The IRT1 transporter would be expected to drive iron and zinc uptake from the soil, and FER1 would be expected to provide a sink for iron storage^19^. Native IRT1 is a high-affinity ferrous-iron transporter necessary for metal uptake and is upregulated under low-iron conditions^20^. The mutant version of IRT1 (IRT1 K146R K171R) that we used maintains an upregulated state under iron-abundant conditions, and efficacy has been demonstrated in *A. thaliana*^18^. We found that overexpression of mutant IRT1 drove elevated iron and zinc accumulation in a crop plant under iron-abundant field conditions (83 µg/g DW iron; Supplementary Fig. 4a). IRT1 can transport manganese, cadmium and cobalt in addition to iron and zinc^21^. Elevated concentrations of toxic heavy metals in biofortified foods is a safety concern. Field-grown cassava plants coexpressing *IRT1* *+* *FER1* accumulated elevated manganese and copper levels, but not to toxic levels (Supplementary Fig. 8a,b).

The cadmium concentrations in greenhouse growth medium and field soil were below detectable levels, a result that may explain why cadmium was not detected in the storage roots of transgenic cassava plants. The potential for *IRT1* *+* *FER1* plants to accumulate cadmium was further tested by growing the high-iron- and high-zinc-accumulating line 8023-19 (Fig. 2c,d) in potting medium spiked with 10 µM cadmium sulfate. Nontransgenic control plants accumulated undetectable cadmium in the control medium, with levels increasing to 0.8 ± 0.3 µg/g DW in leaves and 0.64 ± 0.2 µg/g DW in storage roots, when grown in medium supplemented with cadmium sulfate. Transgenic plants also accumulated cadmium when grown in high-cadmium medium but did so at levels two to five times lower than those in the nontransgenic controls (Supplementary Fig. 9a,b). In the presence of high cadmium, transgenic plants accumulated less iron and zinc in their storage roots than when grown in medium without supplemental cadmium (Supplementary Fig. 9d,f), thus indicating possible competition among cadmium, iron and zinc transport in cassava. Cultivation of cassava plants on medium artificially supplemented with cadmium indicated that *IRT1* *+* *FER1* transgenic plants accumulated cadmium at levels lower than those in nontransgenic controls (Supplementary Fig. 9a,b), thus suggesting that *IRT1* *+* *FER1* transgenic plants would not pose a higher risk of cadmium toxicity than nonmodified plants if grown in high-cadmium soils.

We planted stake cuttings from nontransgenic controls, three *VIT1* (8012-4, 8012-11 and 8012-18) and three *IRT1* *+* *FER1* (8023-8, 8023-10 and 8023-17) transgenic lines in the field to assess the stability of mineral enhancement across the vegetative cropping cycle (Supplementary Fig. 10). After 12 months of growth, the storage roots were harvested and analyzed. All *VIT1* lines showed a significant (*P* ≤ 0.001) six- to seven-times-higher iron concentration than that in nontransgenic controls, reaching a maximum of 62 ± 14 µg/g DW (Supplementary Fig. 11a), levels equivalent to those obtained in the first planting cycle (Fig. 2a). *VIT1* transgenic plants also showed a minor but significant increase in zinc concentration (Supplementary Fig. 11b). Likewise, *IRT1* *+* *FER1* lines established from stake cuttings accumulated iron and zinc, reaching 80 µg/g DW and 60 µg/g DW, respectively, in their storage roots (Supplementary Fig. 11g,h), levels equivalent to the concentrations measured in the first cropping cycle (Fig. 2c,d). At the end of the second 12-month growing period, there were no significant differences between shoot and storage-root yields in two of the *VIT1* transgenic lines (Supplementary Fig. 11c–f). Lower shoot and root yields were observed in *IRT1* *+* *FER1* transgenic plants than in nontransgenic controls over the second cropping cycle (Supplementary Fig. 11i–l). However, the storage-root yields observed for all three transgenic lines remained equivalent to historical averages achieved for the control cultivar TME 204, as measured across five confined field trials previously performed at the Isabela field station, Puerto Rico (Supplementary Fig. 12).

We analyzed the localization of iron and zinc in the stems and storage roots of transgenic plants by using elemental mapping through synchrotron X-ray fluorescence microscopy (XRF)^22^. On the basis of variations in tissue-section thickness and hydration, elemental and Compton-scattering XRF maps were obtained to compare and report elemental distributions (Supplementary Fig. 13a,b). The maps revealed that accumulated iron was associated with vascular tissues of the stem and storage roots in *VIT1* and *IRT1* *+* *FER1* plants (Supplementary Fig. 14). *VIT1* stems and storage roots showed strong localization of iron but minimal localization of zinc (Supplementary Fig. 14b,e), whereas strong colocalization of iron and zinc was seen in *IRT1* *+* *FER1* plants within the same tissue types (Supplementary Fig. 14c,f). In stems, this colocalization was associated with the stele and in the storage root with xylem vessels of the storage parenchyma (Supplementary Fig. 14c,f).

**Fig. 3:**
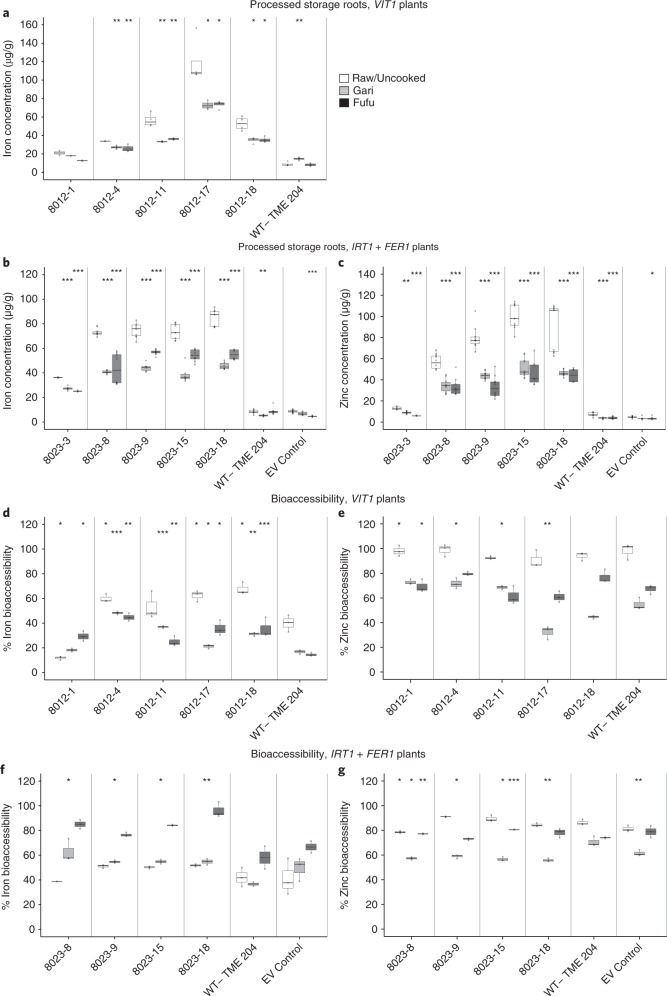
Mineral retention and bioaccessibility of processed *VIT1* and *IRT1* *+* *FER1* storage roots. **a**, Iron concentrations after processing of gari and fufu from the storage roots of *VIT1* plants. **b**,**c**, Iron (**b**) and zinc (**c**) concentrations after processing of gari and fufu from storage roots of *IRT1* *+* *FER1* plants. **d**,**e**, Iron (**d**) and zinc (**e**) bioaccessibility of after processing of gari and fufu from storage roots of *VIT1* plants. **f**,**g**, Iron (**f**) and zinc (**g**) bioaccessibility after processing of gari and fufu from storage roots of *IRT1* *+* *FER1* plants. For *VIT1, n* = 3 biologically independent plants; for *IRT1* *+* *FER1*, *n* = 4 biologically independent plants (2 technical replicates/plant). Box-and-whisker plots were constructed with the R package ggplot2. The upper whisker extends from the hinge to the largest value, no further than 1.5× the IQR from the hinge. The lower whisker extends from the hinge to the smallest value, at most 1.5× the IQR of the hinge. Data beyond the ends of the whiskers are considered outlying points and are plotted individually. Statistical tests were performed with two-sided Student’s *t* test, relative to raw storage roots within each line (**a**–**c**) or to nontransgenic control (**d**). *P ≤ 0.05; **P ≤ 0.01; ***P ≤ 0.001.

To be nutritionally useful, the increased mineral concentrations in transgenic plants must be retained in foodstuffs after processing. Therefore, we assessed the retention and bioaccessibility of iron and zinc in foods prepared from biofortified storage roots. Peeling and boiling of cassava is performed by many communities in East Africa^23^. *VIT1* and *IRT1* *+* *FER1* transgenic storage-root parenchyma tissues showed no significant decrease in iron or zinc content after boiling (Supplementary Fig. 15a–c). Processing to produce the West African cassava foodstuffs gari and fufu^23^ is a more complex process involving chopping, soaking, fermenting, pressing and roasting. Iron retention in gari and fufu reached a minimum of 60% in both *VIT1* and *IRT1* *+* *FER1* storage parenchyma compared with raw roots from the same plants (Fig. 3a,b), thus indicating the release of iron from the food matrix during processing. Zinc concentrations were 25–45% lower in gari and 55–60% lower in fufu than in unprocessed storage roots harvested from *IRT1* *+* *FER1* transgenic lines (Fig. 3c). Importantly, however, equal rates of mineral loss were also found in gari and fufu prepared from storage roots of nontransgenic controls (Fig. 3a–c), thus indicating that the minerals in transgenic plants were retained at levels similar to the baseline levels present in nonmodified tissues. The steps in processing resulting in loss of iron and zinc are unknown, but a similar loss of iron has been reported during milling of rice, millet and wheat^24^, and in cooked cowpea meal^25^.

Bioaccessibility was assessed to determine the potential of availability of iron and zinc present within cassava foods for absorption in the gut after digestion. *VIT1* transgenic storage roots had significantly higher iron bioaccessibility in uncooked roots (up to 1.5 times higher), processed gari (up to 2.8 times higher) and processed fufu (up to 3 times higher) than that of foods processed from the nontransgenic control (Fig. 3d). No differences were observed in zinc bioaccessibility in raw and processed fufu in the *VIT1* transgenic storage roots and nontransgenic controls (Fig. 3e), whereas processed gari had significantly higher levels in three events and lower levels in two transgenic events, as compared with nontransgenic controls (Fig. 3e). No significant differences in iron or zinc bioaccessibility were detected in processed foods from *IRT1* *+* *FER1* and nontransgenic control plants (Fig. 3f,g). Interestingly, *VIT1* transgenic storage roots had significantly lower levels of both iron (20–65%) and zinc (15–65%) bioaccessibility in processed gari and fufu than in uncooked samples from the same plants (Fig. 3d,e). *IRT1* *+* *FER1* transgenic storage roots showed significantly higher levels (27–54%) of iron bioacessibility in processed fufu (Fig. 3f) and significantly lower levels (18–36%) of zinc bioaccessibility in processed gari than in uncooked samples (Fig. 3g). The significantly higher iron bioaccessibility in *VIT1* transgenic plants than in nontransgenic controls (Fig. 3d) may have been due to an association of stored iron with soluble organic acids within the vacuole^26^, whereas the lack of differences in bioaccessibility in *IRT1* *+* *FER1* transgenic plants relative to nontransgenic controls (Fig. 3f,g) may have resulted from iron stored as ferritin being less available for release from the storage-root tissues^19^.

**Fig. 4:**
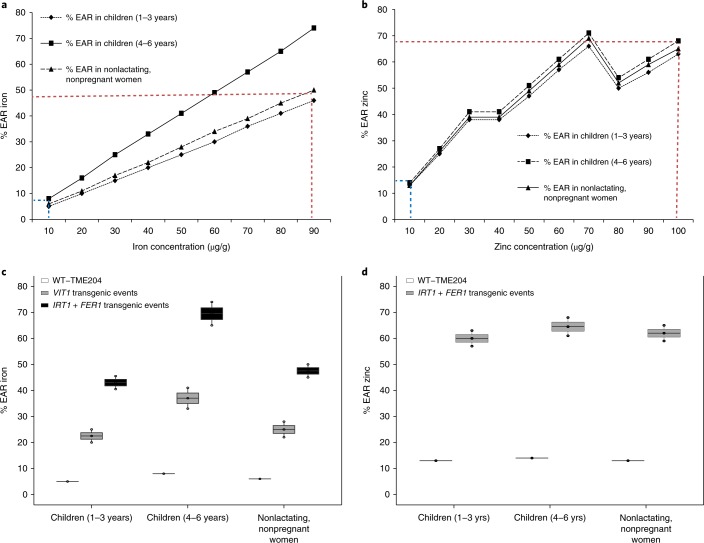
Contribution of biofortified transgenic cassava to EARs for iron and zinc. **a**,**b**, Graphical plots of iron (**a**) and zinc (**b**) concentrations from the transgenic cassava plants against the percentage EAR for children (1–3 years of age), children (4–6 years of age) and nonpregnant nonlactating women. Blue dashed line, percentage EAR calculated from processed food of baseline wild-type storage roots; red dashed line, percentage EAR calculated from processed food of transgenic cassava storage roots. **c**, Iron-biofortified *VIT1* (containing an additional 40–50 µg/g iron) or *IRT1* *+* *FER1* (containing an additional 80–90 µg/g iron) cassava lines, showing the potential nutritional contribution toward the EARs for different demographic groups relative to baseline WT-TME 204 (10 µg/g iron). **d**, Zinc-biofortified *IRT1* *+* *FER1* (containing an additional 90–100 µg/g zinc) cassava lines showing the potential nutritional contribution toward the EARs for different demographic groups relative to baseline WT-TME 204 (10 µg/g zinc).

The nutritional effects of consuming cassava storage roots biofortified by overexpression of *VIT1* and *IRT1* *+* *FER1* was assessed by calculating their potential contribution to the EAR^27^ for iron and zinc. On the basis of consumption patterns in West Africa^28^, iron and zinc present in nonmodified cassava storage roots provide only 5–8% and 13–14% of the EAR for iron and zinc, respectively, for children 1–3 years old (Fig. 4a,d). Our data on mineral accumulation and retention enabled us to predict that *IRT1* *+* *FER1* transgenic plants of TME 204 contributed up to 40–50% of the EAR for iron for children (1–3 years old) and nonlactating, nonpregnant women, and 65–75% of the EAR for iron for children (4–6 years old) (Fig. 4a,c). In addition, *IRT1* *+* *FER1* plants provided 60–70% of the EAR for zinc for children (1–3 years old), children (4–6 years old) and nonlactating, nonpregnant women (Fig. 4b,d).

Caco-2 studies were not undertaken on the iron- and zinc-biofortified foods reported here, because it can assay for only iron bioavailability but does not generate data for zinc bioavailability or provide quantifiable values for mineral release from a digested food. Instead, an in vitro bioaccessibility assay was used to assess the release of iron and zinc from gari and fufu in transgenic versus nontransgenic derived foods, thus enabling calculation of the EAR for both minerals (Fig. 4).

In summary, of the 18 transgenic cassava plant lines coexpressing *IRT1* *+* *FER1* mRNA, 17 (94%) attained nutritionally meaningful levels of iron and zinc that were able to provide 30–50% of the EAR for iron, and 15 attained levels were able to provide 40–70% of the EAR for zinc for children and nonlactating, nonpregnant women (Figs. 2 and 4). The success of our method surpasses that previously reported for approaches to engineer biofortification by using *Agrobacterium*-mediated integration into the plant genome. For example, in rice, more than 1,600 transgenic T_0_ lines were required to generate two low-T-DNA-copy lines with nutritionally meaningful iron and zinc elevation^13^. It is therefore possible that even low-level transgenic expression of mutated *IRT1* may be effective for driving substantial uptake of iron and zinc from the growth medium into plant cells. The loss of iron and zinc during cassava processing occurred at levels higher than expected (Fig. 3), but, crucially, the loss rates were equivalent in transgenic and nontransgenic plants, and the available iron and zinc in processed foods remained meaningful in terms of nutritional value (Fig. 4). Factors such as the levels of vitamin C and organic acids present in the diet can increase mineral bioaccessibility and improve mineral absorption in the digestive tract^29^. Cassava is not a recognized source of organic acids^30^, whereas vitamin C present in fresh storage roots is degraded up to 99% by commonly used processing techniques^8^. The nutritional value of the elevated iron and zinc present in biofortified cassava foodstuffs can be improved by the consumption of other foods in the diet that contain these absorption promoters. Therefore future studies should evaluate the effects of dietary vitamin C levels on mineral micronutrient bioavailability in biofortified cassava foodstuffs.

The United Nations Sustainable Development Goals call for an end to global hunger and decreases in all forms of malnutrition by the year 2030 (ref. ^31^). Nutritional security for the global population could be improved through biofortification of staple food crops such as cassava. We report that iron-enriched and iron- and zinc-enriched cassava storage roots can be grown in the field without decreases in yield. Our biofortified plant lines, or indeed other staple dicot crops such as sweet potato and potato, may be exploited as exemplars to improve the nutritional quality of cassava cultivars grown in different regions.

## Methods

### Generation of transgene constructs, plant transformation and plant materials

The binary vector p8023 bearing dual expression cassettes for *A*. *thaliana* iron-regulated transporter (At*IRT1*, At4g19690.2) and ferritin 1 (At*FER1*, At5g01600) was generated in the p5000 binary vector^32^. The amino acid sequence information for mutated At*IRT1* (ref. ^18^) in binary vector pELC (provided by E. Connolly, University of South Carolina) was used to commercially synthesize this sequence after *M. esculenta* codon optimization. Both the promoter and the 3′ UTR for driving expression of At*IRT1* were obtained from *Arabidopsis* cysteine/histidine-rich C1 domain–containing protein (At5g43040.1) also known as A14 (ref. ^33^). The *A. thaliana* ferritin 1 (AtFER1, At5G01600) expression cassette was generated with the type I patatin promoter and patatin 3′ UTR. Both At*FER1* and At*IRT1* expression cassettes were cloned into the binary vector p5000 (ref. ^32^). The resulting binary vector bearing the T-DNA with At*IRT1*, At*FER1* and the plant selectable marker *npt*II driven by the duplicated CaMV 35 S promoter was named p8023. This construct was electroporated into *Agrobacterium* strain LBA4404 and used for transformation of cassava cultivar TME 204, as described earlier^34^. The mutated version of At*IRT1* is referred to as *IRT1* herein for simplicity.

### Molecular characterization of in vitro– and greenhouse-grown transgenic plants

Total RNA was extracted from 50 mg of leaves obtained from 4- to 6-week-old in vitro–grown cassava plantlets with a Promega RNA isolation kit, as described earlier^17^ and RT–PCR reactions were performed with At*IRT1*- and At*FER1*-specific primers (Table 1). For RT–qPCR, total RNA was extracted from 50 mg of leaves, fibrous roots and lyophilized storage roots obtained from 16-week-old greenhouse-grown plants^17^, and RT–qPCR reactions were performed with At*IRT1-* and At*FER1-*specific primers (Table 1). Transcript levels in transgenic and wild-type plants were normalized to the expression level of protein phosphatase 2 A with the relative standard-curve method^17^.

**Table 1:** Primers used in the study and their purposes

Name	Sequence	Purpose
At*IRT1*	F, 5′-GCTTCGGACTTGTAAGATTCATCAGA-3′	RT–PCR
	R, 5′-TCATTCTGTTGTGATCGGACTTTCC-3′	
At*FER1*	F, 5′-TGAGACGATAGGGTGGAGTTTCAC-3′	RT–PCR
	R, 5′-ACCGGAGTCGTGTTCCAGCCTT-3′	
Cassava tubulin	F, 5′-GATCCTACTGGGAAGTACATTGG-3′	RT–PCR
	R, 5′-CTGCATTCTCCACCAACTGA-3′	
At*IRT1*	F, 5′-TCCATCAGCTTCGGACTTGTAAGA-3′	RT–qPCR
	R, 5′-GGAGGAATGTCCATTATCGCCA-3′	
At*FER1*	F, 5′-GAGGTTGAAATACTTAAATGCGTGC-3′	RT–qPCR
	R, 5′-TAAGGATGATCGGCAAAGGCCA-3′	
Cassava protein phosphatase 2 A	F, 5′-TGCAAGGCTCACACTTTCATC-3′	RT–qPCR
	R, 5′-CTGAGCGTAAAGCAGGGAAG-3′	

F, forward; R, reverse

### Plant establishment and growth in the greenhouse

In vitro transgenic and wild-type plantlets were planted in Fafard 51 mixture potting compost (Conrad Fafard) and established in the greenhouse^35^. For all experiments, eight independent transgenic plant lines were established, and plants were grown at 32 °C/27 °C (day/night) with 70–95% relative humidity. Plants were watered with reverse-osmosis water two or three times per day, as required, and fertilized twice weekly with Jack’s professional fertilizer (JR Peters) at a rate of 100 µg/g DW (ref. ^35^).

### Determination of total biomass in the greenhouse

Total biomass was determined for transgenic and wild-type plants after 16 weeks of growth in the greenhouse. Senescent leaves were collected in brown bags on alternate days over the 16-week period and dried for 4–5 d in an oven at 60 °C. After 16 weeks, fresh weights of leaves, petioles, stems, fibrous roots, storage-root peels and storage-root parenchyma were recorded. Collected tissues were dried as described above. Samples of storage-root parenchyma (20–25 g fresh weight) were placed in 50-mL conical tubes, frozen at −80 °C for 2–3 h and freeze-dried with a lyophilizer (Freezone18, Labconco) for at least 48 h. Freeze-dried storage-root samples were weighed to determine dry-matter content, and total biomass per plant was calculated.

### Determination of T-DNA copy number

T-DNA copy number was determined by extraction of genomic DNA from 1–2 g of young leaves obtained from 4- to 6-week-old greenhouse-grown transgenic and control plants with the CTAB protocol. Genomic DNA (20 μg) from each sample was digested with 80 units of SpeI (New England BioLabs) overnight, subjected to electrophoresis on a 1% agarose gel and transferred with standard procedures. A 2× 35 S promoter (50 ng) was used as a probe. Hybridization and washing procedures were performed as previously described^34^.

### Growth of transgenic plants in medium spiked with cadmium

Plants were treated with cadmium by transferring wild-type plants and plants transgenic for p8023 into 3-inch pots, weaning in a mist chamber for 1 week (ref. ^35^) and growing on an open bench at 26 °C/25 °C (day/night) for 2 weeks. After 4 weeks, plants were transferred to a growth chamber and maintained under a 16 h/8 h photoperiod at a 35 °C/30 °C (day/night) temperature cycle and 80–90% relative humidity for 13 weeks. Plants were watered with reverse-osmosis water two or three times per day, as required, and fertilized twice weekly with Jack’s professional fertilizer (JR Peters) at a rate of 100 p.p.m. (ref. ^35^). One set of plants was treated with 10 μM CdSO_4_ added along with the fertilizer twice per week, and the other was maintained as a control with a 0 μM cadmium treatment. Four biological replicates were maintained for each control and transgenic plant line. After 13 weeks, leaves and storage roots were harvested and processed for measurement of minerals by ICP–OES.

### Establishment and execution of confined field trials

Confined field trials were conducted at the Isabela Agriculture Research Station, Puerto Rico, Mayaguez. Soil samples (~250 g) were obtained from 10 cm below the soil surface with a wooden spatula, and chemical analysis was performed by A&L Analytical Laboratories (Supplementary Fig. 4). In vitro plantlets were established in 50-mL conical tubes and shipped from DDPSC, St. Louis, to the University of Puerto Rico. Plantlets were transferred to Rain Forest potting soil (Sungro) in 4-inch pots and hardened for 1 month under light shelves (12 h light), followed by an additional 1 month in a glasshouse before being planted in the field. Confined field trials were established with a randomized block design with three replicates and six plants per line per replicate (Supplementary Fig. 4). A distance of 1.5 m was maintained between plants across all plants in the trial, which in turn were surrounded by a single row of nontransgenic wild-type plants. Drip irrigation was used in the dry season; otherwise, the trials were rain fed. The final harvest was performed 12 months after planting, and storage roots were harvested manually. At harvest, agronomic traits including shoot biomass, number of roots and root yield were determined. The harvest index was calculated as the storage root fresh weight divided by the total fresh weight of storage roots plus shoot biomass on a per-plant basis. Dry-matter content was assessed by lyophilization of 35 g fresh weight of storage-root parenchyma for 2 d. Three storage roots per transgenic line for each field-plot replicate were cleaned, dried, waxed and shipped to DDPSC, St. Louis. After receipt, the storage roots were peeled, chopped with ceramic knives and lyophilized as previously described^17^, in preparation for elemental and bioaccessibility analysis. Stake-derived field trials were established by obtaining stem cuttings from nontransgenic controls, three *VIT1* (8012-4, 8012-11 and 8012-18) and three *IRT1* *+* *FER1* (8023-8, 8023-10 and 8023-17) transgenic lines at the time of harvest, 12 months after planting from in vitro plants. Stake cuttings were obtained from wood-stem material 6.0–8.0 inches in length and 1.5–2.0 inches in diameter, comprising five to seven nodes. Stakes were used to establish a randomized block design with four replicates and 20 plants per line per replicate. Other conditions remained the same as described above.

### Measurement of mineral concentrations

All plant tissues were harvested and dried for 48 h in a 60 °C oven. Two subsamples (~0.5 g dry weight each) of ground, homogenized dried tissue were predigested overnight with 3 mL of ultrapure nitric acid in borosilicate glass tubes. Samples were digested as previously described^36^, and elemental concentrations were determined by ICP–OES (CIROS ICP Model FCE12; Spectro). Peach-leaf standards (SRM 1547 A; National Institute of Standards and Technology) were digested and analyzed along with each run of experimental samples to verify reliability of the procedures and analytical measurements; all values for peach-leaf standards were within their certified range. Mineral-content determinations were calculated by multiplication of each sample concentration by the total dry weight of that tissue sample.

### Mineral analysis by synchrotron X-ray fluorescence spectroscopy (µ-XRF)

Synchrotron-based XRF was used to obtain elemental concentrations and Compton scattering at the F3 station of the Cornell High Energy Synchrotron Source (CHESS), Cornell University. A double-crystal Si (220) monochromator was used to select an incident beam energy of 11.94 keV (Δ*E*/*E* ~10^−4^), and a single-bounce monocapillary lens (capillary PeB605)^37^ was used to focus the beam to a 20-µm-diameter spot. For scans, stem and storage-root thin cross-sections (0.2–0.3 mm thick) were obtained from live-tissue samples, weighed and mounted between one layer of 6.5-µm Kapton polyimide film and a layer of 25-µm Kapton tape, such that the thinner layer faced the detector. Fluorescence was measured with a 384-element Maia detector placed upstream of the sample and operating in backscatter geometry^38^. XRF images were obtained by continuous scanning of the sample horizontally across the beam, with typical integration times of 0.005 s/pixel. For petioles, stems and storage roots, two sections were obtained from plants of 8012-5, 8012-11 (*VIT1* transgenic plants) and 8023-9, 8023-15 (*IRT1* *+* *FER1* transgenic plants) and one each of the wild type and the empty-vector controls. Petioles were mounted alongside the stems. Sections were mounted and scanned simultaneously. Fluorescence data were analyzed with the dynamic analysis method to obtain elemental maps with the software GeoPIXE v7.1. The incident flux was calibrated with reference films of known weight concentration, which were used to calculate elemental-weight concentrations from XRF peak areas via a fundamental-parameters approach.

### Determination of total linamarin concentration in field-grown storage roots

Total linamarin concentrations were assessed in field-grown storage roots. Approximately 10 mg of peeled storage-root tissue was lyophilized to a dry powder and extracted with buffer containing 100 mg/mL 2% acetonitrile, 1% HCOOH and 10 μM phenyl‐β‐d‐glucoside by vortexing for 15 min at room temperature. Samples was centrifuged to remove debris and filtered through an 0.8-μm polyethersulfone spin filter. One microliter of each sample was injected, and reversed-phase LC–MS/MS was performed with a 0.5 × 100-mm polymeric reversed phase (PLRPS) column and 0.1% HCOOH in water and acetonitrile as solvents. Sodiated linamarin and β-glucopyranoside (Sigma) were used as internal standards.

### Mineral retention in foodstuffs generated from processed storage roots

Retention studies was performed on boiled storage roots and processed gari and fufu samples prepared from field-grown transgenic storage roots. Three biological replicates from five independent transgenic lines of each *VIT1* and *IRT1* *+* *FER1* storage roots were assessed. Approximately 10 g of fresh peeled storage root tissues/line/replicate was chopped and boiled in 200 mL MQ water for 10–12 min. Boiled samples were patted dry with paper towels, lyophilized and digested for ICP–OES elemental analysis. Processing of storage roots for gari and fufu food products was performed as described earlier^23^. For gari preparation, storage root (150–200 g fresh weight) was peeled and grated, fermented in sacks and pressed with a hydraulic jack between wooden platforms to remove excess liquid from the pulp. Dewatered and fermented pulp was dried at room temperature for 48–72 h, then forced through a 16-inch sieve mesh (10 mm) (Winco Industries). Fine pulp was roasted in a griddle at approximately 275 °C for 10–15 min until the gari turned light brown. Fufu preparation was achieved by steeping 150–200 g fresh peeled roots in water for 3–4 d. Tissues were grated and blended in a food processor (Magic Bullet, Homeland Housewares), then pressed with a hydraulic jack between wooden platforms to remove water. Samples were dried in an oven at 90 °C for 24 h and milled to a powder with a food blender. Gari and fufu along with uncooked storage root samples were subjected to ICP–OES elemental analysis.

### Bioaccessibility studies

Uncooked field-grown transgenic and wild-type cassava storage roots, plus gari, fufu and control samples were subjected to bioaccessibility studies. The bioaccessibility assays were performed in triplicate with a model simulating the digestive process in the mouth, stomach (gastric digestion) and small intestine (intestinal digestion). Enzyme solutions used for the in vitro digestion were prepared according to Glahn et al.^39^, with some modifications. Shortly before use, α-amylase, pepsin and pancreatin/bile solutions were prepared separately as follows. (i) For α-amylase solution, α-amylase, Chelex-100 resin and 140 mM NaCl were mixed in a ratio of 1.0 g/2.5 g/15 mL, respectively. (ii) to For pepsin solution, 208 mg of pepsin and 1.9 g of Chelex-100 resin were dissolved in 7.5 mL 0.1 M HCl. (iii) For pancreatin/bile solution, 30 mg pancreatin, 150 mg bile and 3.5 g Chelex-100 resin were dissolved in 7.5 mL 0.6 M NaHCO_3_. Each solution was mixed by being kept at room temperature for 30 min with vortexing every 5 min. Solutions were filtered by gravity flow through a 30-µm mesh filter to remove the Chelex-100 resin from the solution, and each filtrate was recovered. Chelex treatments were used to decrease the background levels of minerals associated with the enzyme reagents. The reagents α-amylase (1.5 units/mg protein), porcine pepsin (800–2,500 units/mg protein), porcine bile extract and pancreatic enzymes (4× USP) were obtained from Sigma–Aldrich. Chelex-100 resin was obtained from Bio-Rad. In vitro digestion was performed according to Glahn et al.^39^ with some modifications. Simulation of digestion in the mouth was performed in a centrifuge tube by addition of 9 mL of α-amylase solution to 1 g of freeze-dried sample; the mixture was incubated for 10 min at room temperature. Simulation of digestion in the stomach was carried out by addition of 10 mL 140 mM NaCl, 5 mL of 5 mM KCl and 1 mL of pepsin solution to the prior mixture, and the pH of the solution was adjusted to 2 by addition of concentrated HCl. This mixture was kept in an incubator shaker at 37 °C at 260 r.p.m. for 2 h, and simulation of intestinal digestion was carried out in the same tube by addition of 1 mL of pancreatin/bile solution and 0.6 M NaHCO_3_ until the pH reached 5.7. The mixture was incubated for 2 h at 37 °C and then placed into an ice bath to stop the digestion. The tube was centrifuged at 3,750 r.p.m. for 30 min, and approximately 21 mL of supernatant was recovered. The recovered supernatant was split into two samples of 10 mL, each aliquot was dried down to approximately 1 mL, and samples were processed for elemental analysis. The semidried samples were predigested with 1 mL concentrated HNO_3_ for 16 h at room temperature in 150-mL digestion tubes, and most of the CO_2_ was allowed to bubble out of the solution. An additional 2 mL of concentrated HNO_3_ was added, and samples were heated for 1 h at 90 °C, then at 125 °C until fuming stopped, after which 1.5 mL of H_2_O_2_ was added, and the sample was maintained at 125 °C for 1 h. This process was repeated an additional time, and samples were then dried at 200 °C, and the residues were dissolved in 7 mL of 2% HNO_3_. Mineral concentrations were determined by ICP–OES (Ciros ICP- FCE12). Certified standards were used to calibrate the ICP–OES instrument. ‘Blank’ samples, consisting of digestion solutions with no cassava material, were used to determine background levels of minerals from the bioaccessibility solutions. Duplicate 0.5 g (dry weight) aliquots of each of the uncooked, gari or fufu samples were digested and analyzed by ICP–OES as described above, to determine the starting mineral concentrations of each sample. Duplicate mineral concentrations were averaged and converted to an average content (1 g basis). Mineral concentrations in the duplicate supernatant samples were averaged and multiplied by the number of milliliters of digestion solution (sum of all volumes added in the bioaccessibility assay) and converted to average mineral amounts released from each sample (normalized to a 1 g basis). Bioaccessibility percentages were then calculated with these content values: percentage bioaccessibility = (amount of each mineral released × 100)/starting content of each mineral. Triplicate aliquots of each food sample (uncooked root, gari or fufu) were used to calculate a mean and s.d. for each sample.

### Determination of EARs

The EAR is a commonly used method to determine and express the average daily nutrient intake estimated to meet requirements of half the healthy individuals in a particular life stage and sex group within a given population. Variables that contribute to nutrient intake include the amount of food consumed per day, nutrients retained after boiling/cooking, proportion of nutrients absorbed and additional nutrient concentration present in the biofortified food. Nutrient requirements depend on the age and sex of the target population. The EAR was determined by dividing the physiological requirement of the nutrient by the fractional nutrient absorption. The potential nutritional contribution of iron-biofortified cassava lines toward the EARs for different demographic groups were calculated on the basis of assumptions made from food consumption^28^ (66.7 g DW/d), nutrient retention (70%), absorbed proportion (5%) and nutrient required (460 µg) for children 1–3 years of age; on the basis of assumptions made from food consumption (116.7 g DW/d), nutrient retention (70%), absorbed proportion (5%) and nutrient required (500 µg) for children 4–6 years of age; and on the basis of assumptions made from food consumption (233.3 g DW/d), nutrient retention (70%), absorbed proportion (5%) and nutrient required (1,460 µg) for nonlactating, nonpregnant women (Fig. 4a, c). Zinc-biofortified cassava lines showing the potential nutritional contribution toward the EARs for different demographic groups were calculated on the basis of assumptions made from food consumption (66.7 g DW/d), nutrient retention (70%), absorbed proportion (15%) and nutrient required (740 µg) for children 1–3 years of age; on the basis of assumptions made from food consumption (116.7 g DW/d), nutrient retention (70%), absorbed proportion (15%) and nutrient required (1200 µg) for children 4–6 years of age; and on the basis of assumptions made from food consumption (233.3 g DW/d), nutrient retention (70%), absorbed proportion (15%) and nutrient required (2,500 µg) for nonlactating, nonpregnant women^40,41^ (Fig. 4b,d). Nutrient absorption was calculated by multiplication of the food consumed per day with the additional nutrient concentration, nutrient retention and absorbed proportion of the food^6^.

### Statistical analysis

The construction of graphics and the statistical analysis were performed with R software, version 3.4.0 (ref. ^42^). Box-and-whisker plots were constructed with the R package ggplot2 (ref. ^43^). The upper whisker extends from the hinge to the largest value no further than 1.5× the IQR from the hinge (where IQR is the distance between the first and third quartiles). The lower whisker extends from the hinge to the smallest value, at most 1.5× the IQR of the hinge. Data beyond the end of the whiskers were deemed outlying points and are plotted individually. To generate *P* values as indicated in the graphics, a two-sample *t* test was performed, comparing the wild type to the transgenic lines, unless otherwise stated.

### Reporting Summary

Further information on research design is available in the Nature Research Reporting Summary linked to this article.

## Online content

Any methods, additional references, Nature Research reporting summaries, source data, statements of data availability and associated accession codes are available at 10.1038/s41587-018-0002-1.

## Supplementary Information

### Integrated supplementary information

Supplementary Figure 1 Transgenic plants overexpressing IRT1 + FER1. (**a**) Schematic representation of the *IRT1* *+* *FER1* T-DNA construct for genetic transformation in cassava. RB and LB symbolizes the right and left borders of the T-DNA respectively, A14: epidermal promoter from *Arabidopsis*, *AtIRT1*: iron regulated transporter from *Arabidopsis*, 3’ *A14*: 3’UTR from A14, 35 s polyA: 3’ UTR from Cauliflower mosaic virus, patatin: promoter from potato, *AtFER1*: ferritin storage protein from *Arabidopsis, 3’ pat*: 3’ UTR from patatin, 3’Nos: 3’ UTR from *Agrobacterium*. (**b**-**d**) Phenotype (**e**-**g**) storage roots of 16-week old wildtype TME 204 and *IRT1* *+* *FER1* transgenic plants grown under greenhouse conditions. Scale bars at the lower left correspond to 1 cm. (**h**) Leaf iron (**i**) leaf zinc (**j**) storage root iron and (**k**) storage root zinc concentrations of *IRT1* *+* *FER1* transgenic plants. For *IRT1* *+* *FER1*, n = 4 biologically independent plants. Box and whisker plots were constructed using the R software package The upper whisker extends from the hinge to the largest value no further than 1.5* IQR from the hinge (where IQR is the inter-quartile range, or distance between the first and third quartiles). The lower whisker extends from the hinge to the smallest value at most 1.5* IQR of the hinge. Data beyond the end of the whiskers are called “outlying” points and are plotted individually. Statistical tests were two-sided using Student’s t-test compared to non-transgenic control. *, ** and *** represent significance levels at *p* ≤ 0.05, *p* ≤ 0.01 and *p* ≤ 0.001 respectively.

Supplementary Figure 2 Molecular characterization of IRT1 + FER1 transgenic plants. (**a**) PCR screening for presence of *IRT1* and *FER1* transgenes in leaves of 4-week old *in vitro* plants. Tubulin was used as a control. M- marker; WT- wildtype; EV- empty vector; N- negative water control; (**b**) Genomic DNA blot hybridization from wildtype and transgenic *IRT1* *+* *FER1* cassava leaves (6-week old) to determine T-DNA insertion number. Positive represents the plasmid DNA. Both gel and blots were cropped for better resolution. Quantitative expression of *IRT1* and *FER1* in (**c**, **f**) leaves, (**d**, **g**) fibrous roots and (**e**, **h**) storage roots of *IRT1* *+* *FER1* transgenic cassava plants. Tissues were collected from 16-week-old plants grown in the greenhouse. Expression was compared and normalized to protein phosphatase 2 (pp2A). Line 8023-2 expression values were adjusted to a value of 1 and all other expression values expressed relative to this line. Values are means of 4 biologically independent plants and error bars represent SD. This experiment was repeated 2 times independently with similar results.

Supplementary Figure 3 Total iron and zinc content of IRT1 + FER1 transgenic cassava plants under greenhouse conditions. (**a**) Iron and (**b**) zinc. Tissues were collected from 16-week old cassava plants and subjected to ICP-OES elemental analysis. Values are means of four biological independent plants. Bars represent SD. *, and ** stands for significant difference, respectively, at *p* *≤* *0.05, p* *≤* *0.01*, using Student’s t-test compared to wildtype. WT; wild type. EV control, empty vector control plants.

Supplementary Figure 4 Plot layout of VIT1 and IRT1 + FER1 transgenic lines. **a**) Chemical properties of soil at Isabela field station, University of Puerto Rico, Mayaguez, PR, USA. (**b**) Plot layout of replicated field trial of *VIT1* transgenic lines in 2014. (**c**) Plot layout of *IRT1* *+* *FER1* transgenic lines in 2015. WT; wildtype, X; buffer row with wildtype plants.

Supplementary Figure 5 Agronomic harvest data of VIT1 transgenic plants. (**a**) Shoot yield, (**b**) number of roots, (**c**) harvest index, (**d**) dry matter content and (**e**) linamarin concentration of *VIT1* transgenic plants and non-transgenic control plants grown in confined field trial conditions. For *VIT1*, n = 9 biologically independent plants (3 plants/replicate). Box and whisker plots were constructed using the R software package The upper whisker extends from the hinge to the largest value no further than 1.5* IQR from the hinge (where IQR is the inter-quartile range, or distance between the first and third quartiles). The lower whisker extends from the hinge to the smallest value at most 1.5* IQR of the hinge. Data beyond the end of the whiskers are called “outlying” points and are plotted individually. Statistical tests were two-sided using Student’s t-test compared to non-transgenic control. *, **, and *** represent significance levels at *p* ≤ 0.05, *p* ≤ 0.01 and *p* ≤ 0.001 respectively.

Supplementary Figure 6 Agronomic harvest data of IRT1 + FER1 transgenic plants. (**a**) Shoot yield, (**b**) number of roots, (**c**) harvest index, (**d**) dry matter content and (**e**) linamarin concentration of *IRT1* *+* *FER1* transgenic plants and control plants grown in the confined field trial at University of Puerto Rico, Mayaguez. For *IRT1* *+* *FER1*, n = 4 biologically independent plants.). Box and whisker plots were constructed using the R software package The upper whisker extends from the hinge to the largest value no further than 1.5* IQR from the hinge (where IQR is the inter-quartile range, or distance between the first and third quartiles). The lower whisker extends from the hinge to the smallest value at most 1.5* IQR of the hinge. Data beyond the end of the whiskers are called “outlying” points and are plotted individually. Statistical tests were two-sided using Student’s t-test compared to non-transgenic control. *, ** and *** represent significance levels at *p* ≤ 0.05, *p* ≤ 0.01 and *p* ≤ 0.001 respectively.

Supplementary Figure 7 Other mineral concentrations of storage roots of VIT1 transgenic cassava plants under field conditions 12 months after planting. (**a**) Manganese, (**b**) Nickel, (**c**) Copper concentration. For *VIT1*, n = 9 biologically independent plants (3 plants/replicate).). Box and whisker plots were constructed using the R software package The upper whisker extends from the hinge to the largest value no further than 1.5* IQR from the hinge (where IQR is the inter-quartile range, or distance between the first and third quartiles). The lower whisker extends from the hinge to the smallest value at most 1.5* IQR of the hinge. Data beyond the end of the whiskers are called “outlying” points and are plotted individually. Statistical tests were two-sided using Student’s t-test compared to non-transgenic control. *, **, and *** represent significance levels at *p* ≤ 0.05, *p* ≤ 0.01 and *p* ≤ 0.001 respectively.

Supplementary Figure 8 Other mineral concentrations of storage roots of IRT1 + FER1 transgenic cassava plants under field conditions 12 months after planting. (**a**) Manganese and (**b**) copper concentration. For *IRT1* *+* *FER1*, n = 4 biologically independent plants. Box and whisker plots were constructed using the R software package The upper whisker extends from the hinge to the largest value no further than 1.5* IQR from the hinge (where IQR is the inter-quartile range, or distance between the first and third quartiles). The lower whisker extends from the hinge to the smallest value at most 1.5* IQR of the hinge. Data beyond the end of the whiskers are called “outlying” points and are plotted individually. Statistical tests were two-sided using Student’s t-test compared to non-transgenic control. *, ** and *** represent significance levels at *p* ≤ 0.05, *p* ≤ 0.01 and *p* ≤ 0.001 respectively.

Supplementary Figure 9 Response of IRT1 + FER1 transgenic plants to culture in high-cadmium conditions. Plants were treated with 10 μM CdSO_4_ (grey bars) twice a week for with a 0 μM treatment (white bars). (**a**) Leaf cadmium, (**c**) iron and (**e**) zinc concentrations of WT and *IRT1* *+* *FER1* transgenic plants grown at low and high cadmium. Storage root (**b**) cadmium, (**d**) iron and (**f**) zinc concentrations of WT and *IRT1* *+* *FER1* transgenic plants grown at low and high cadmium. Values are means of 4 biologically independent plants. Box and whisker plots were constructed using the R software package The upper whisker extends from the hinge to the largest value no further than 1.5* IQR from the hinge (where IQR is the inter-quartile range, or distance between the first and third quartiles). The lower whisker extends from the hinge to the smallest value at most 1.5* IQR of the hinge. Data beyond the end of the whiskers are called “outlying” points and are plotted individually. Statistical tests were two-sided using Student’s t-test compared to non-transgenic control. *, ** and *** represent significance levels at *p* ≤ 0.05, *p* ≤ 0.01 and *p* ≤ 0.001 respectively.

Supplementary Figure 10 IRT1 + FER1 transgenic plants 10 months after establishment from stem cuttings growing under confined-field-trial conditions at Isabela Research Station, University of Puerto Rico. CFT was established using a randomized block design with 4 reps and 20 plants/line/rep.

Supplementary Figure 11 Stake-derived VIT1, IRT1 + FER1 transgenic and nontransgenic control cassava plants grown under field conditions. **a**) Iron, (**b**) Zinc concentration of storage roots (**c**) Shoot yield, (**d**) root yield, (**e**) number of roots and (**f**) harvest index from stake-derived *VIT1* transgenic and non-transgenic control cassava plants grown under field conditions. (**g**) Iron, (**h**) Zinc concentration of storage roots (**i**) Shoot yield, (**j**) root yield, (**k**) number of roots and (**l**) harvest index from stake-derived *IRT1* *+* *FER1* transgenic and non-transgenic control cassava plants. Values are means of 20 biologically independent plants in 4 replicates (5 plants per replicate). Box and whisker plots were constructed using the R software package The upper whisker extends from the hinge to the largest value no further than 1.5* IQR from the hinge (where IQR is the inter-quartile range, or distance between the first and third quartiles). The lower whisker extends from the hinge to the smallest value at most 1.5* IQR of the hinge. Data beyond the end of the whiskers are called “outlying” points and are plotted individually. Statistical tests were two-sided using Student’s t-test compared to non-transgenic control. *, ** and *** represent significance levels at *p* ≤ 0.05, *p* ≤ 0.01 and *p* ≤ 0.001 respectively.

Supplementary Figure 12 Average storage root yields of wild-type TME 204 (gray bars) and empty-vector controls (white bars) under field conditions across different confined field trials, harvested 12 months after planting. For CFT 27 and CFT 30, n = 3 biologically independent plants; CFT 31, n = 9 (3 plants per replicate); CFT33, n = 8; CFT 36 and 37, n = 20 (5 plants per replicate). Box and whisker plots were constructed using the R software package The upper whisker extends from the hinge to the largest value no further than 1.5* IQR from the hinge (where IQR is the inter-quartile range, or distance between the first and third quartiles). The lower whisker extends from the hinge to the smallest value at most 1.5* IQR of the hinge. Data beyond the end of the whiskers are called “outlying” points and are plotted individually. Statistical tests were two-sided using Student’s t-test compared to non-transgenic control. *, ** and *** represent significance levels at *p* ≤ 0.05, *p* ≤ 0.01 and *p* ≤ 0.001 respectively.

Supplementary Figure 13 Elemental and Compton scattering maps of VIT1 and IRT1 + FER1 transgenic cassava stems and petioles. **a**) Elemental and Compton scattering maps obtained from a single scan (run 14761) of a series of transgenic, empty vector control (EV) and non-transgenic control cassava stems and petioles. Scan area was 96 mm × 15 mm with 0.02 mm pitch, and obtained with a collection time of 0.005 seconds/pixel for a total scan time of approximately 5 h. **b**) Elemental and Compton scattering maps obtained from a single scan (run 14762) of a series of transgenic, empty vector (EV) and wild type cassava storage roots. Scan area was 118 mm × 13 mm with 0.02 mm pitch, and obtained with a collection time of 0.006 seconds/pixel for a total scan time of approximately 7 h. The maximum intensity of each image has been scaled independently.

Supplementary Figure 14 Elemental mapping by synchrotron XRF of VIT1 and IRT1 + FER1 transgenic cassava stems and storage roots. Composite, 2D elemental concentration maps of wet, as cut thin sections of (**a**, **d**) wild type, (**b**, **e**) *VIT1* and (**c**, **f**) *IRT1* *+* *FER1* cassava (**a**-**c**) stems and (**d**-**f**) storage roots. Relative concentrations of iron, zinc and calcium are represented as the intensity of red, green and blue respectively. The brightness has been scaled separately for each element, but is identical for each sample type. Scale bars at the lower left correspond to 1 mm. The color guide at mid-right illustrates the appearance of overlapping colors. In particular, yellow regions in (**c**) indicate strong co-localization of Fe and Zn in vascular bundles in *IRT1* *+* *FER1* stems.

Supplementary Figure 15 Mineral retention of raw (white bars) and boiled (gray bars) VIT1 and IRT1 + FER1 storage roots. Fe concentration after (**a**) boiling from storage roots of *VIT1* transgenic cassava plants. (**b**) Fe and (**c**) Zn concentration after boiling from storage roots of *IRT1* *+* *FER1* transgenic cassava plants. For *VIT1*, n = 3 biologically independent plants and for *IRT1* *+* *FER1*, n = 4 biologically independent plants (2 technical replicates/plant). Box and whisker plots were constructed using the R software package The upper whisker extends from the hinge to the largest value no further than 1.5* IQR from the hinge (where IQR is the inter-quartile range, or distance between the first and third quartiles). The lower whisker extends from the hinge to the smallest value at most 1.5* IQR of the hinge. Data beyond the end of the whiskers are called “outlying” points and are plotted individually. Statistical tests were two-sided using Student’s t-test compared to raw storage roots. *, ** and *** represent significance levels at *p* ≤ 0.05, *p* ≤ 0.01 and *p* ≤ 0.001 respectively.

### Supplementary information

Supplementary Text and Figures Supplementary Figures 1–15

Reporting Summary

## Data Availability

The datasets generated and/or analyzed during the current study are available from the corresponding author on reasonable request. All data generated or analyzed during this study are included in this published article and its supplementary information files.
