# Author Correction: Biofortification of field-grown cassava by engineering expression of an iron transporter and ferritin

**DOI:** 10.1038/s41587-019-0066-6

**Published:** 2019-02-20

**Authors:** Narayanan Narayanan, Getu Beyene, Raj Deepika Chauhan, Eliana Gaitán-Solís, Jackson Gehan, Paula Butts, Dimuth Siritunga, Ihuoma Okwuonu, Arthur Woll, Dulce M. Jiménez-Aguilar, Erick Boy, Michael A. Grusak, Paul Anderson, Nigel J. Taylor

**Affiliations:** 1 Donald Danforth Plant Science Center, St. Louis, MO USA; 2 University of Puerto Rico Mayagüez, Puerto Rico, USA; 3 National Root Crops Research Institute, Umudike, Nigeria; 4 Cornell High Energy Synchrotron Source, Cornell University, Ithaca, NY USA; 5 USDA-ARS Children’s Nutrition Research Center, Baylor College of Medicine, Houston, TX USA; 6 Harvest Plus/International Food Policy Research Institute, Washington, DC USA; 7 USDA-ARS Edward T. Schafer Agricultural Research Center, Fargo, ND USA

**Keywords:** Biotechnology, Plant biotechnology, Field trials, Molecular engineering in plants, Plant sciences

Correction to: *Nature Biotechnology* 10.1038/s41587-018-0002-1, published online 28 January 2019.

In the version of this article initially published, a relevant work was not cited. The following sentence has been inserted following the sentence ending “*Aspergillus* phytase” in the third paragraph of the article: “Overexpression of *AtIRT1, AtNAS1* and bean *FERRITIN* in rice resulted in 3.8-fold higher iron and 1.8-fold higher zinc concentrations than in the wild-type control^12^.” A corresponding reference has been added: 12. Boonyaves, K., Wu, T. Y., Gruissem, W. & Bhullar, N. K. Enhanced grain iron levels in rice expressing an *IRON-REGULATED METAL TRANSPORTER, NICOTIANAMINE SYNTHASE*, and *FERRITIN* gene cassette. *Front. Plant Sci*. **8**, 130 (2017). The error has been corrected in the HTML and PDF versions of the article.

