# Supplementary figures and images for: Biofortification of field-grown cassava by engineering expression of an iron transporter and ferritin
Source: Nat Biotechnol. 2019 Jan 28;37(2):144–51. doi: 10.1038/s41587-018-0002-1 (PMC6784895; doi:10.1038/s41587-018-0002-1)

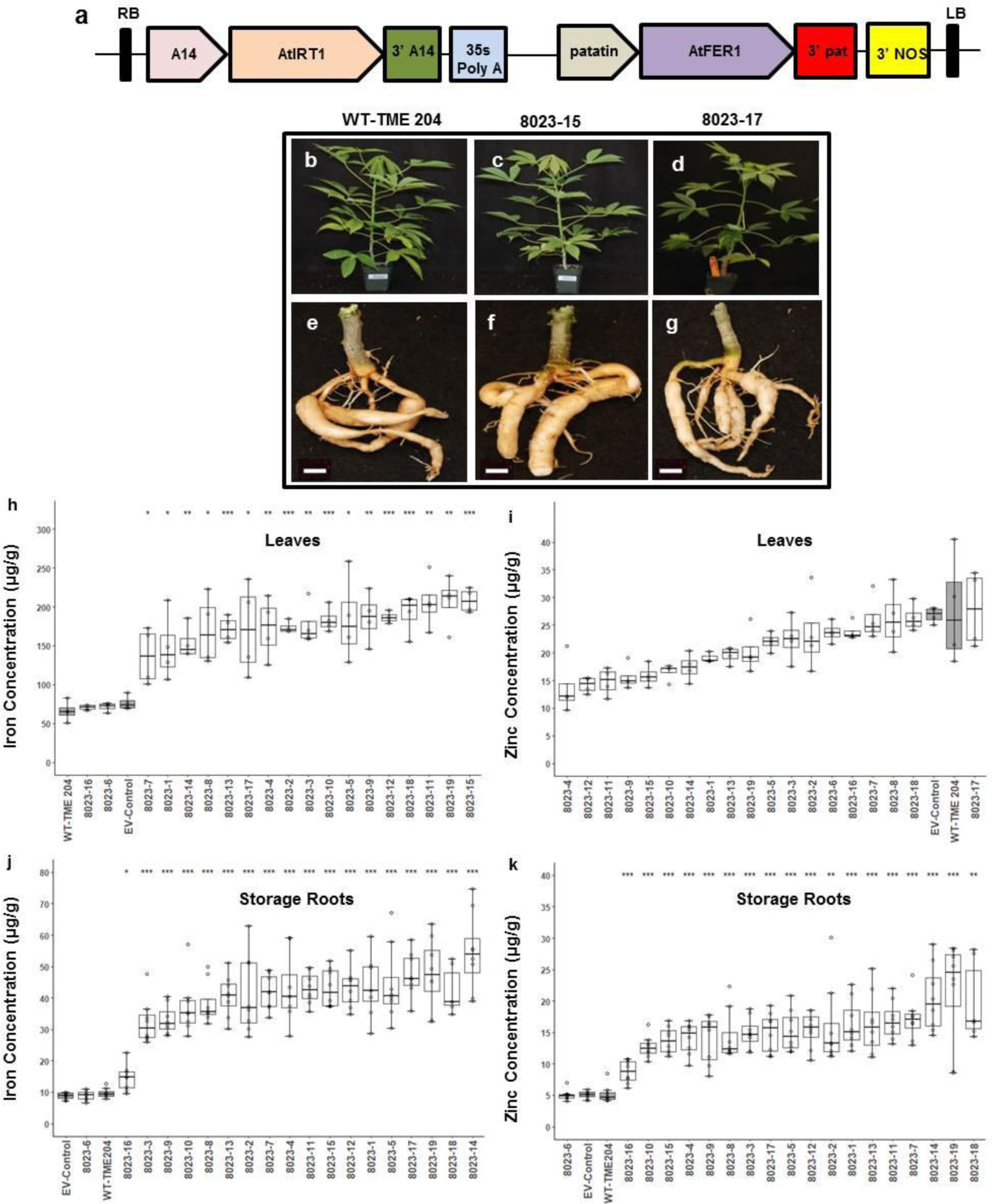

Supplement: Transgenic plants overexpressing IRT1 + FER1. — (a) Schematic representation of the IRT1 + FER1 T-DNA construct for genetic transformation in cassava. RB and LB symbolizes the right and left borders of the T-DNA respectively, A14: epidermal promoter from Arabidopsis, AtIRT1: iron regulated transporter from Arabidopsis, 3’ A14: 3’UTR from A14, 35 s polyA: 3’ UTR from Cauliflower mosaic virus, patatin: promoter from potato, AtFER1: ferritin storage protein from Arabidopsis, 3’ pat: 3’ UTR from patatin, 3’Nos: 3’ UTR from Agrobacterium. (b-d) Phenotype (e-g) storage roots of 16-week old wildtype TME 204 and IRT1 + FER1 transgenic plants grown under greenhouse conditions. Scale bars at the lower left correspond to 1 cm. (h) Leaf iron (i) leaf zinc (j) storage root iron and (k) storage root zinc concentrations of IRT1 + FER1 transgenic plants. For IRT1 + FER1, n = 4 biologically independent plants. Box and whisker plots were constructed using the R software package The upper whisker extends from the hinge to the largest value no further than 1.5* IQR from the hinge (where IQR is the inter-quartile range, or distance between the first and third quartiles). The lower whisker extends from the hinge to the smallest value at most 1.5* IQR of the hinge. Data beyond the end of the whiskers are called “outlying” points and are plotted individually. Statistical tests were two-sided using Student’s t-test compared to non-transgenic control. *, ** and *** represent significance levels at p ≤ 0.05, p ≤ 0.01 and p ≤ 0.001 respectively. [file 41587_2018_2_Fig5_ESM.jpg]

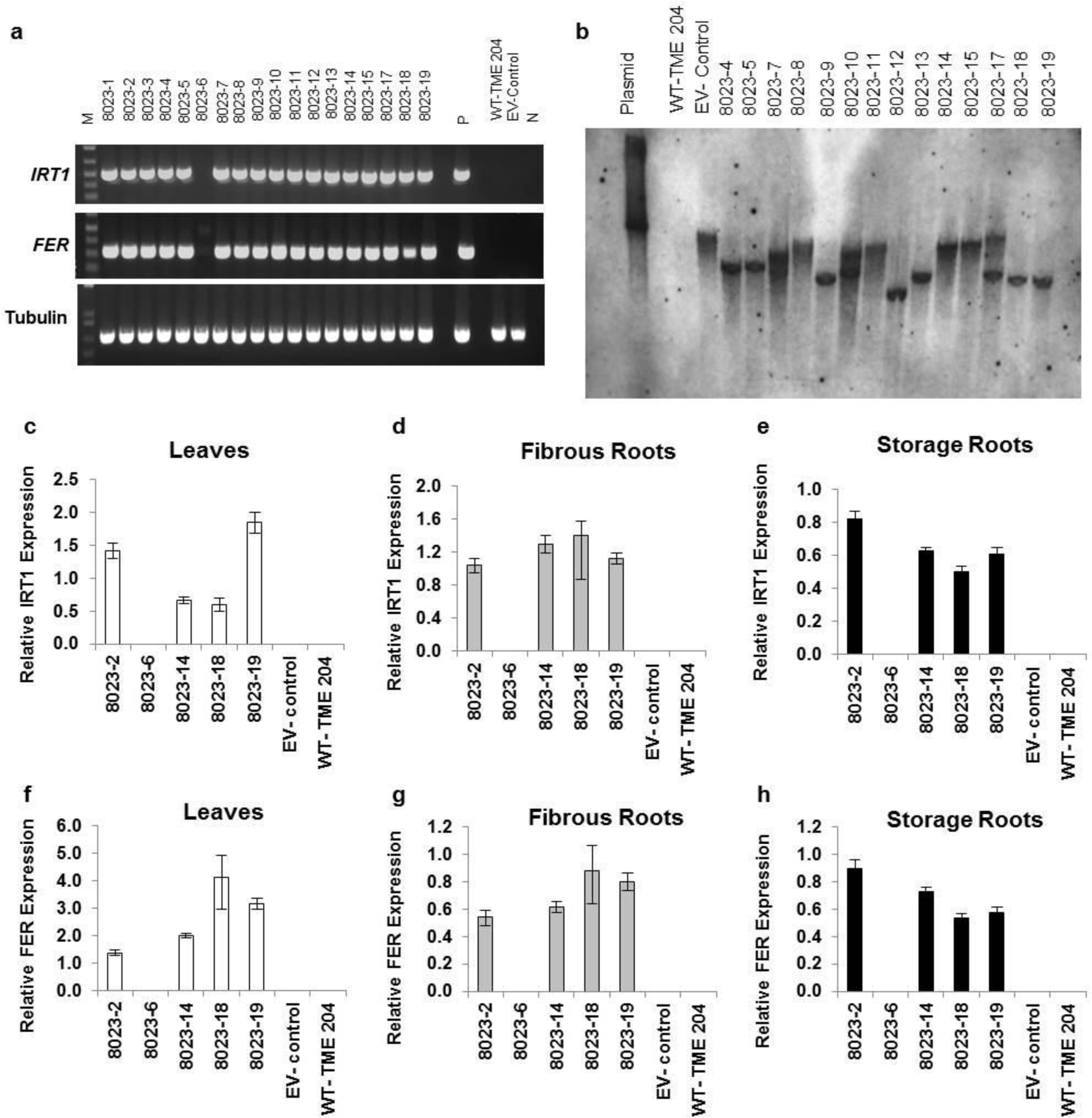

Supplement: Molecular characterization of IRT1 + FER1 transgenic plants. — (a) PCR screening for presence of IRT1 and FER1 transgenes in leaves of 4-week old in vitro plants. Tubulin was used as a control. M- marker; WT- wildtype; EV- empty vector; N- negative water control; (b) Genomic DNA blot hybridization from wildtype and transgenic IRT1 + FER1 cassava leaves (6-week old) to determine T-DNA insertion number. Positive represents the plasmid DNA. Both gel and blots were cropped for better resolution. Quantitative expression of IRT1 and FER1 in (c, f) leaves, (d, g) fibrous roots and (e, h) storage roots of IRT1 + FER1 transgenic cassava plants. Tissues were collected from 16-week-old plants grown in the greenhouse. Expression was compared and normalized to protein phosphatase 2 (pp2A). Line 8023-2 expression values were adjusted to a value of 1 and all other expression values expressed relative to this line. Values are means of 4 biologically independent plants and error bars represent SD. This experiment was repeated 2 times independently with similar results. [file 41587_2018_2_Fig6_ESM.jpg]

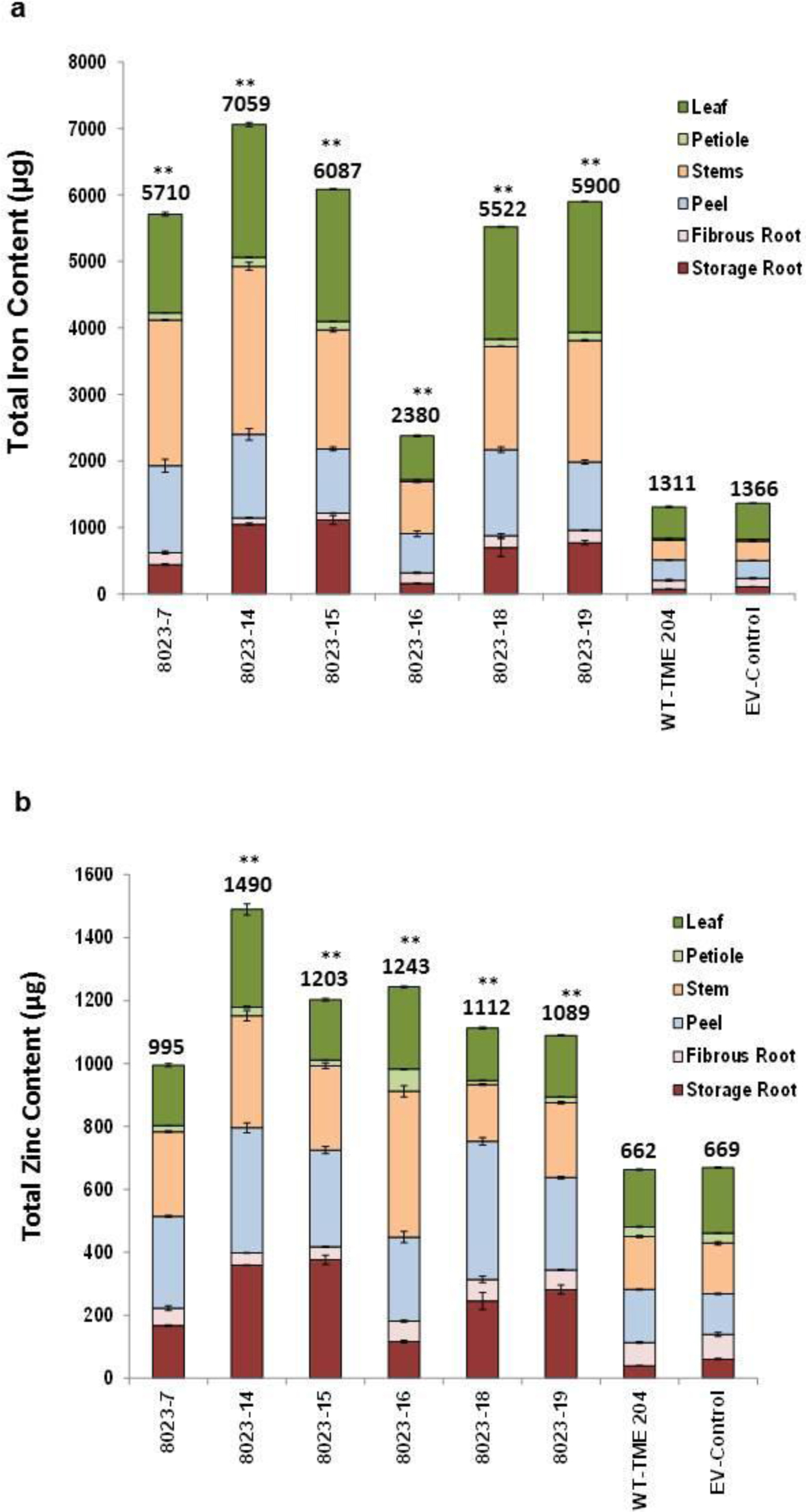

Supplement: Total iron and zinc content of IRT1 + FER1 transgenic cassava plants under greenhouse conditions. — (a) Iron and (b) zinc. Tissues were collected from 16-week old cassava plants and subjected to ICP-OES elemental analysis. Values are means of four biological independent plants. Bars represent SD. *, and ** stands for significant difference, respectively, at p ≤ 0.05, p ≤ 0.01, using Student’s t-test compared to wildtype. WT; wild type. EV control, empty vector control plants. [file 41587_2018_2_Fig7_ESM.jpg]

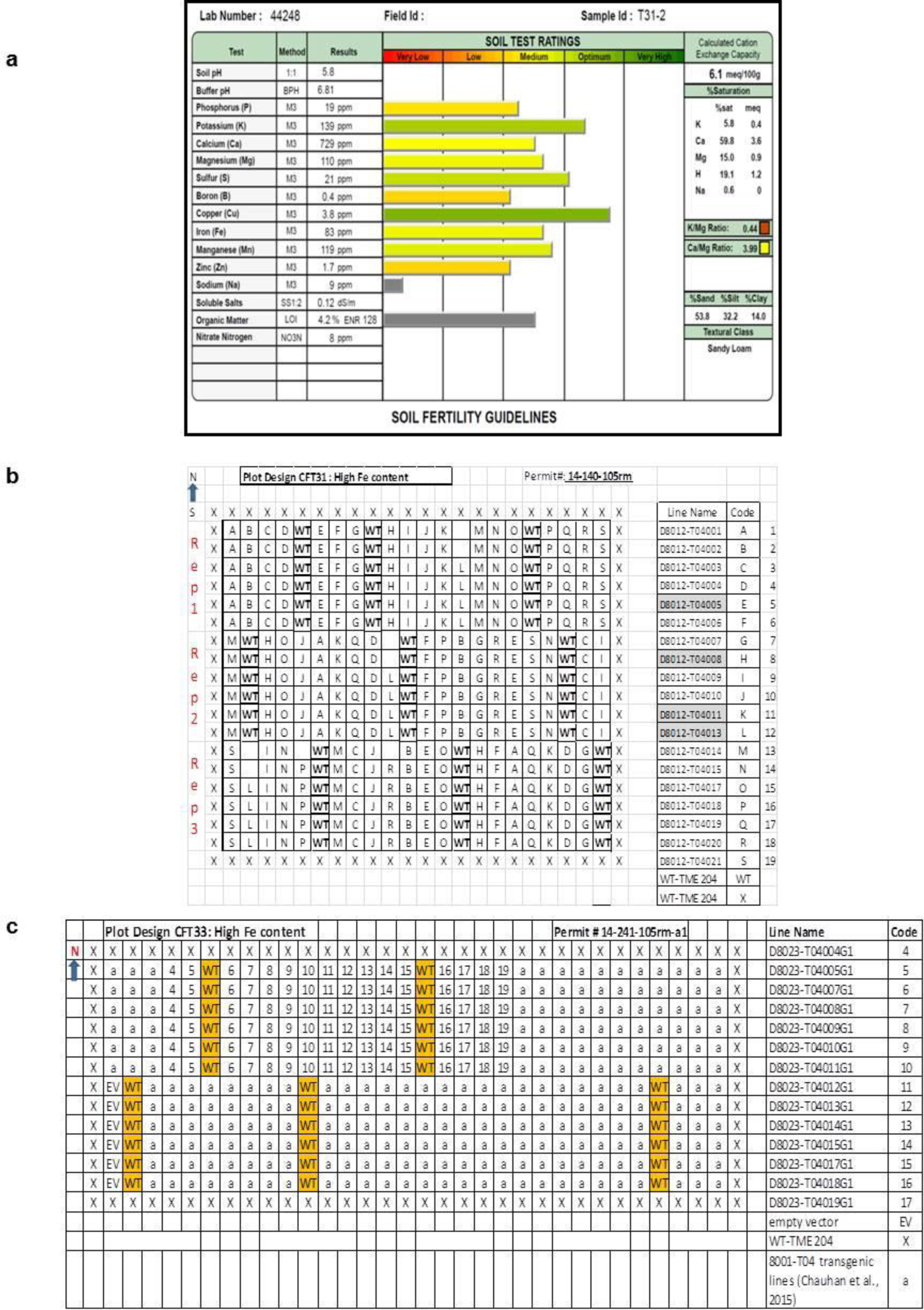

Supplement: Plot layout of VIT1 and IRT1 + FER1 transgenic lines. — a) Chemical properties of soil at Isabela field station, University of Puerto Rico, Mayaguez, PR, USA. (b) Plot layout of replicated field trial of VIT1 transgenic lines in 2014. (c) Plot layout of IRT1 + FER1 transgenic lines in 2015. WT; wildtype, X; buffer row with wildtype plants. [file 41587_2018_2_Fig8_ESM.jpg]

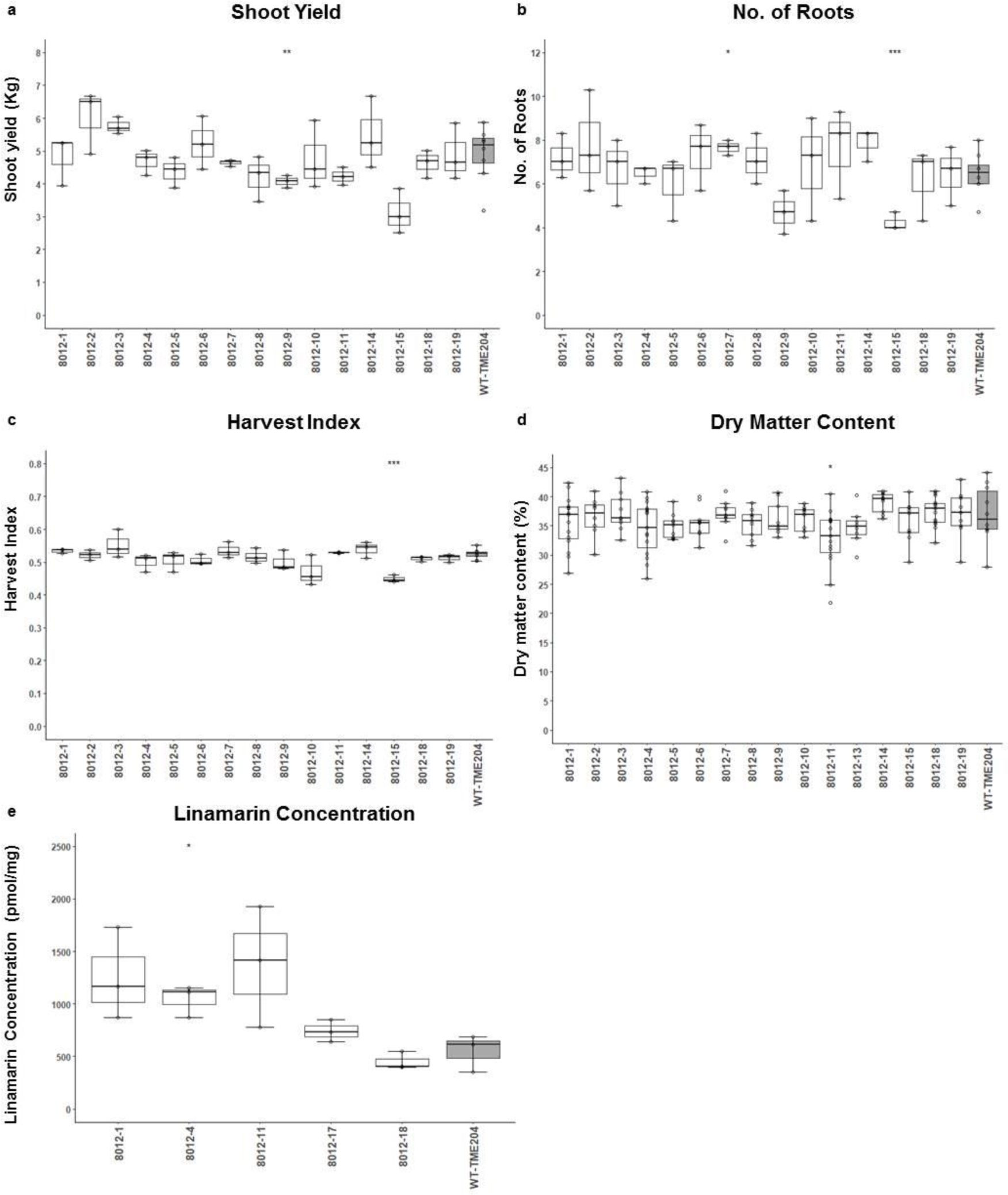

Supplement: Agronomic harvest data of VIT1 transgenic plants. — (a) Shoot yield, (b) number of roots, (c) harvest index, (d) dry matter content and (e) linamarin concentration of VIT1 transgenic plants and non-transgenic control plants grown in confined field trial conditions. For VIT1, n = 9 biologically independent plants (3 plants/replicate). Box and whisker plots were constructed using the R software package The upper whisker extends from the hinge to the largest value no further than 1.5* IQR from the hinge (where IQR is the inter-quartile range, or distance between the first and third quartiles). The lower whisker extends from the hinge to the smallest value at most 1.5* IQR of the hinge. Data beyond the end of the whiskers are called “outlying” points and are plotted individually. Statistical tests were two-sided using Student’s t-test compared to non-transgenic control. *, **, and *** represent significance levels at p ≤ 0.05, p ≤ 0.01 and p ≤ 0.001 respectively. [file 41587_2018_2_Fig9_ESM.jpg]

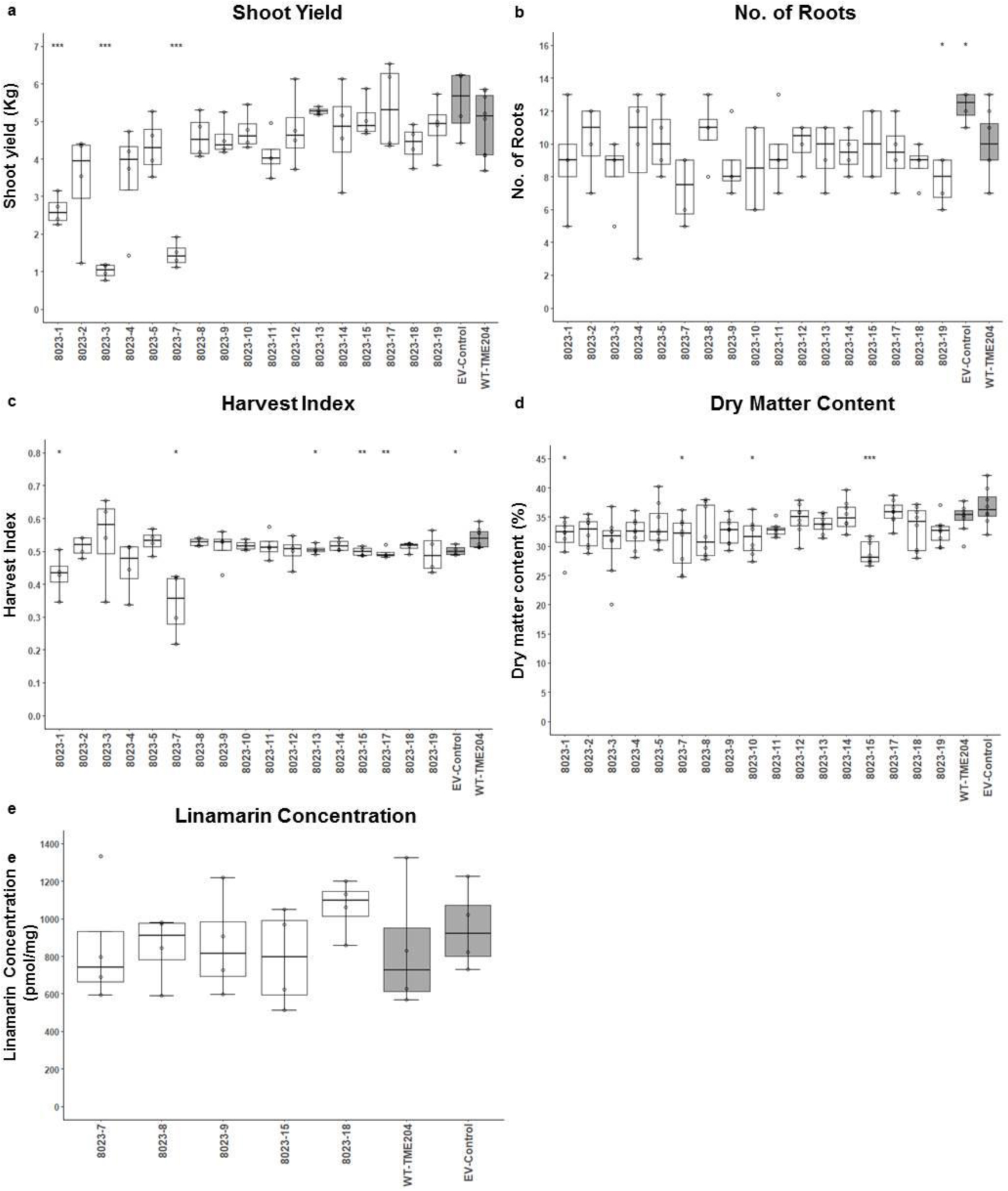

Supplement: Agronomic harvest data of IRT1 + FER1 transgenic plants. — (a) Shoot yield, (b) number of roots, (c) harvest index, (d) dry matter content and (e) linamarin concentration of IRT1 + FER1 transgenic plants and control plants grown in the confined field trial at University of Puerto Rico, Mayaguez. For IRT1 + FER1, n = 4 biologically independent plants.). Box and whisker plots were constructed using the R software package The upper whisker extends from the hinge to the largest value no further than 1.5* IQR from the hinge (where IQR is the inter-quartile range, or distance between the first and third quartiles). The lower whisker extends from the hinge to the smallest value at most 1.5* IQR of the hinge. Data beyond the end of the whiskers are called “outlying” points and are plotted individually. Statistical tests were two-sided using Student’s t-test compared to non-transgenic control. *, ** and *** represent significance levels at p ≤ 0.05, p ≤ 0.01 and p ≤ 0.001 respectively. [file 41587_2018_2_Fig10_ESM.jpg]

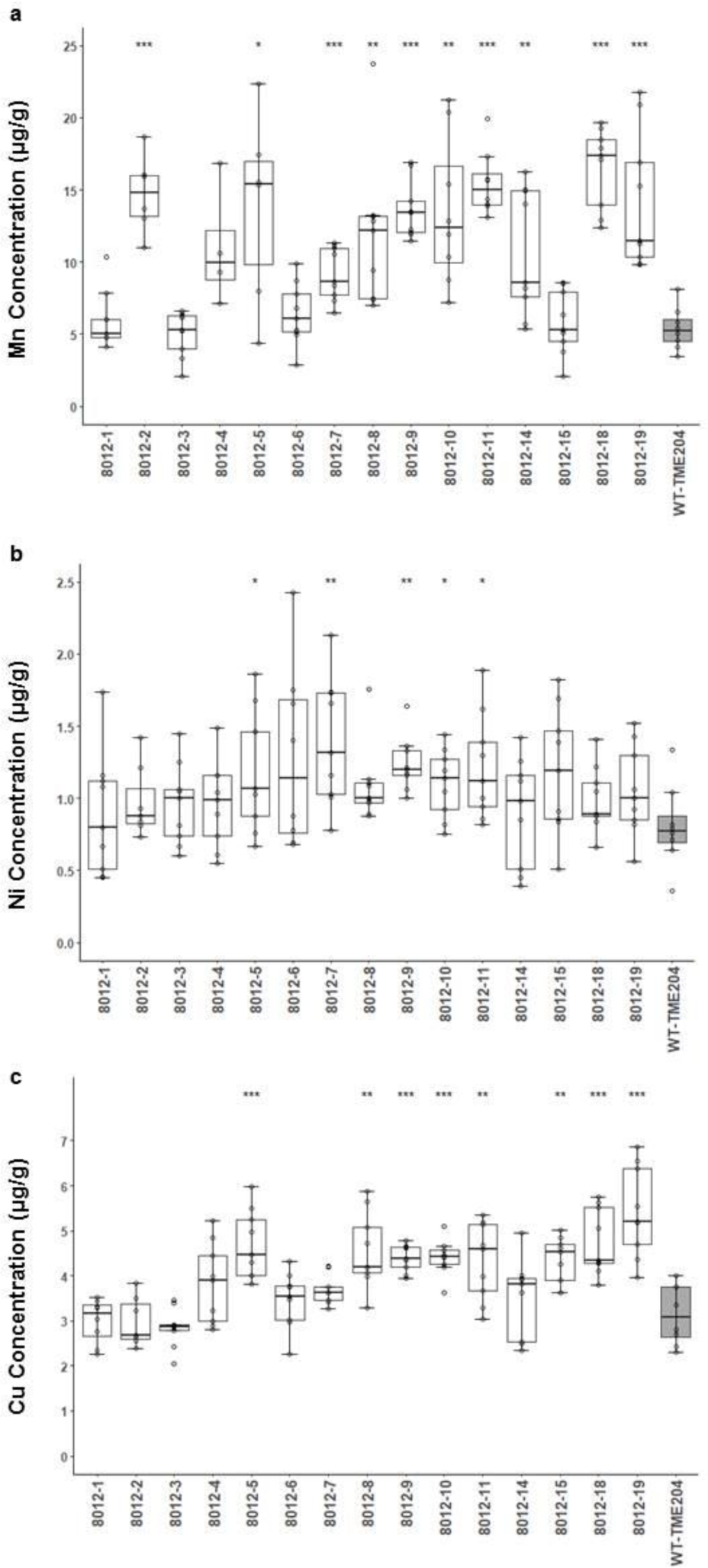

Supplement: Other mineral concentrations of storage roots of VIT1 transgenic cassava plants under field conditions 12 months after planting. — (a) Manganese, (b) Nickel, (c) Copper concentration. For VIT1, n = 9 biologically independent plants (3 plants/replicate).). Box and whisker plots were constructed using the R software package The upper whisker extends from the hinge to the largest value no further than 1.5* IQR from the hinge (where IQR is the inter-quartile range, or distance between the first and third quartiles). The lower whisker extends from the hinge to the smallest value at most 1.5* IQR of the hinge. Data beyond the end of the whiskers are called “outlying” points and are plotted individually. Statistical tests were two-sided using Student’s t-test compared to non-transgenic control. *, **, and *** represent significance levels at p ≤ 0.05, p ≤ 0.01 and p ≤ 0.001 respectively. [file 41587_2018_2_Fig11_ESM.jpg]

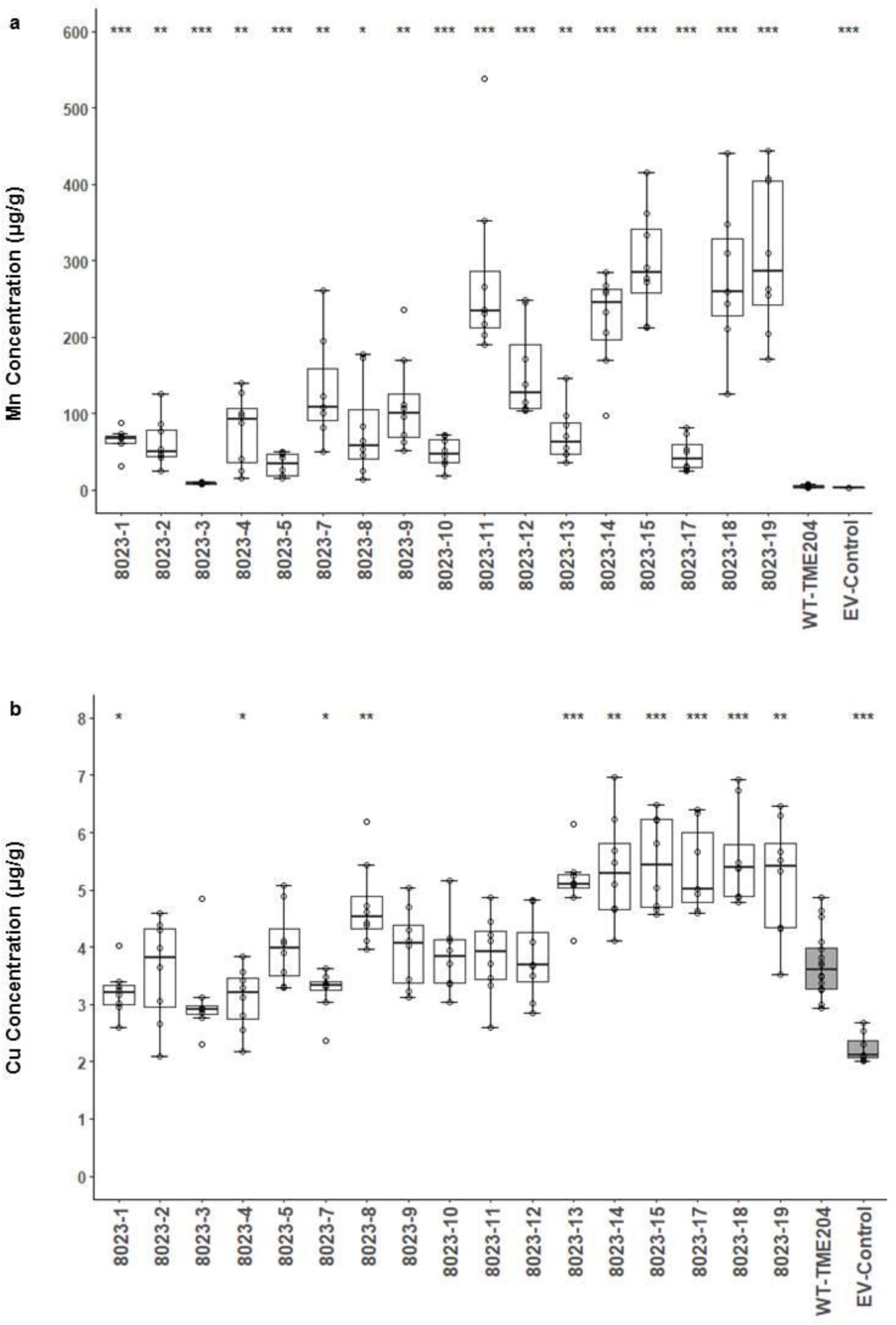

Supplement: Other mineral concentrations of storage roots of IRT1 + FER1 transgenic cassava plants under field conditions 12 months after planting. — (a) Manganese and (b) copper concentration. For IRT1 + FER1, n = 4 biologically independent plants. Box and whisker plots were constructed using the R software package The upper whisker extends from the hinge to the largest value no further than 1.5* IQR from the hinge (where IQR is the inter-quartile range, or distance between the first and third quartiles). The lower whisker extends from the hinge to the smallest value at most 1.5* IQR of the hinge. Data beyond the end of the whiskers are called “outlying” points and are plotted individually. Statistical tests were two-sided using Student’s t-test compared to non-transgenic control. *, ** and *** represent significance levels at p ≤ 0.05, p ≤ 0.01 and p ≤ 0.001 respectively. [file 41587_2018_2_Fig12_ESM.jpg]

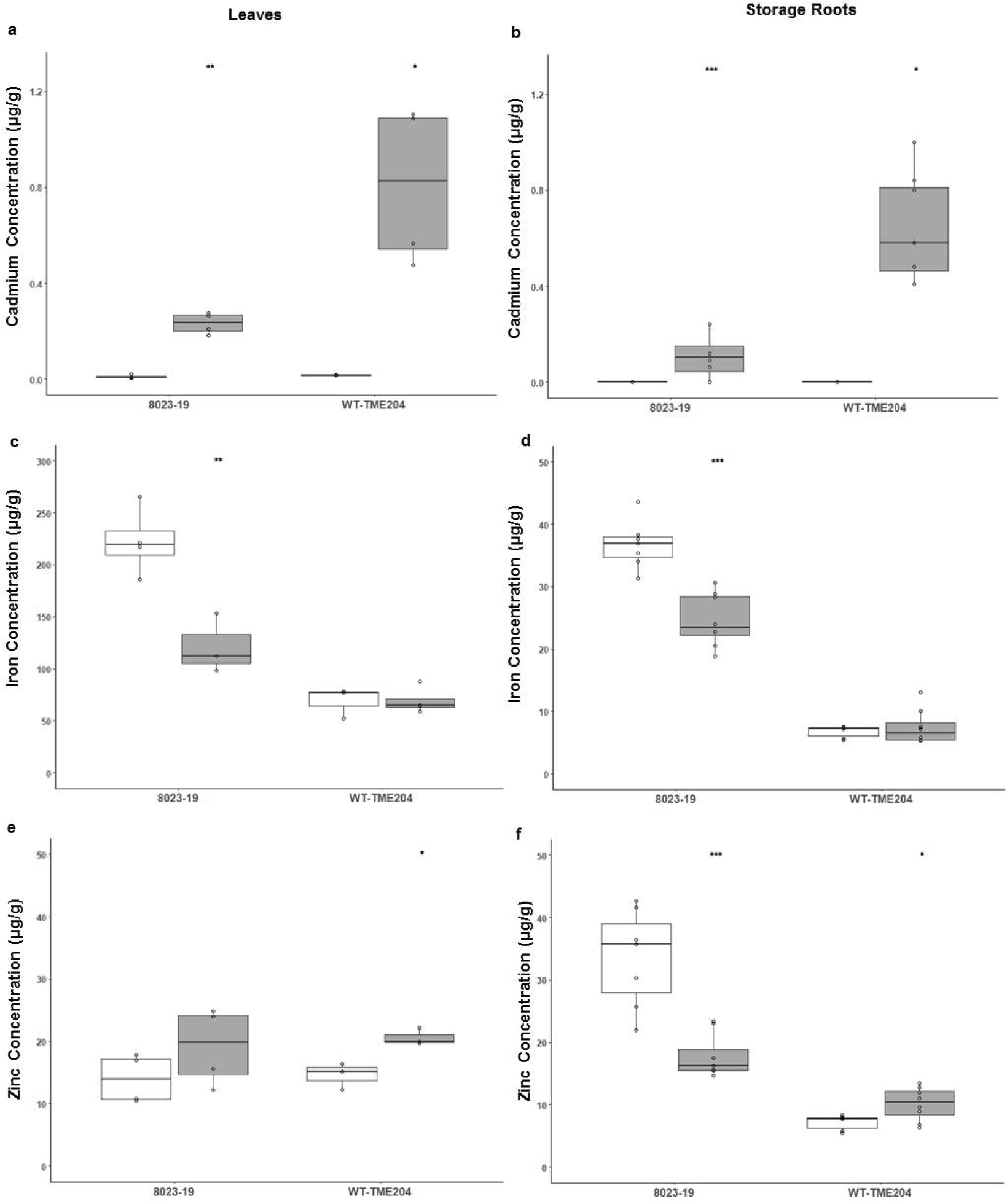

Supplement: Response of IRT1 + FER1 transgenic plants to culture in high-cadmium conditions. — Plants were treated with 10 μM CdSO4 (grey bars) twice a week for with a 0 μM treatment (white bars). (a) Leaf cadmium, (c) iron and (e) zinc concentrations of WT and IRT1 + FER1 transgenic plants grown at low and high cadmium. Storage root (b) cadmium, (d) iron and (f) zinc concentrations of WT and IRT1 + FER1 transgenic plants grown at low and high cadmium. Values are means of 4 biologically independent plants. Box and whisker plots were constructed using the R software package The upper whisker extends from the hinge to the largest value no further than 1.5* IQR from the hinge (where IQR is the inter-quartile range, or distance between the first and third quartiles). The lower whisker extends from the hinge to the smallest value at most 1.5* IQR of the hinge. Data beyond the end of the whiskers are called “outlying” points and are plotted individually. Statistical tests were two-sided using Student’s t-test compared to non-transgenic control. *, ** and *** represent significance levels at p ≤ 0.05, p ≤ 0.01 and p ≤ 0.001 respectively. [file 41587_2018_2_Fig13_ESM.jpg]

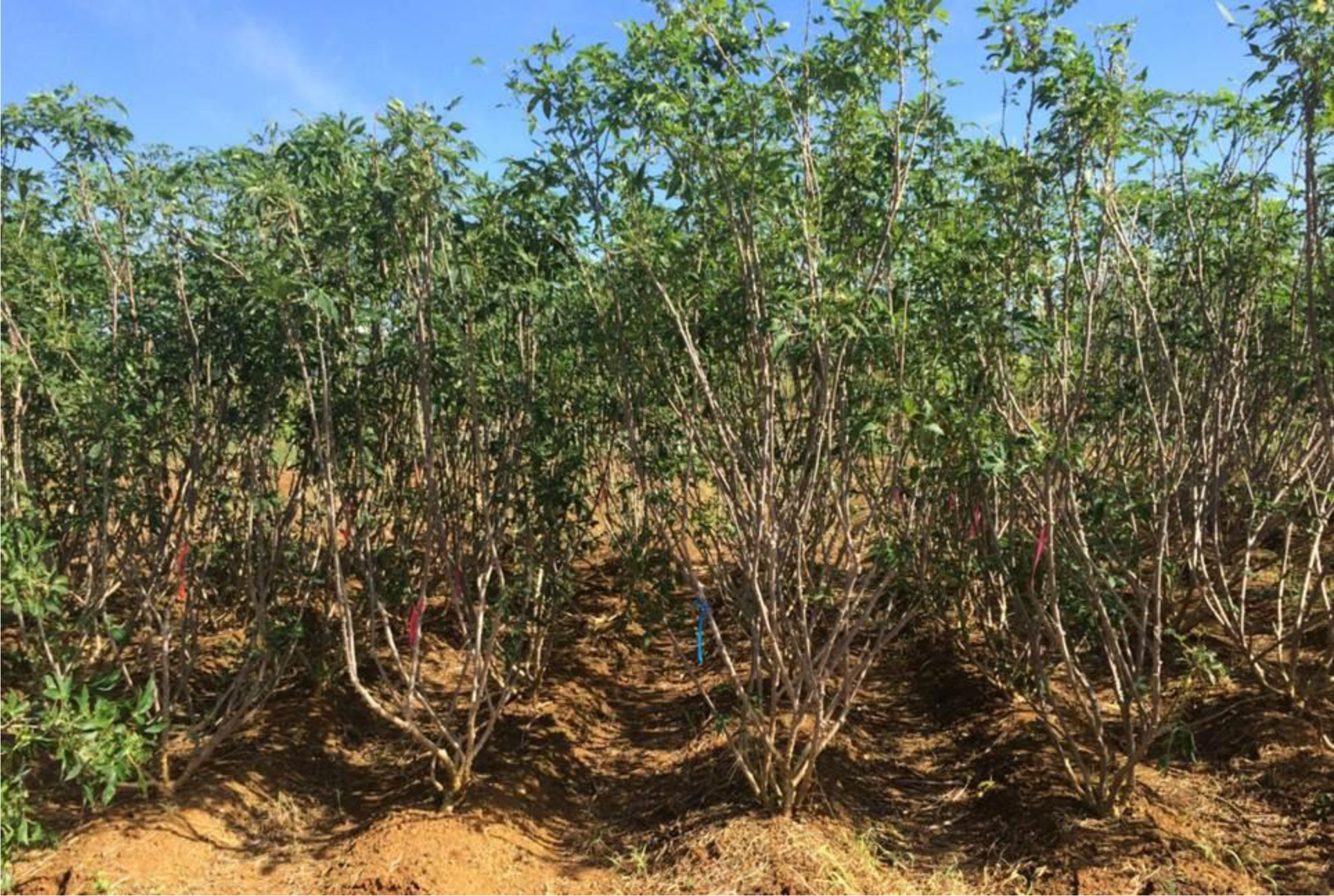

Supplement: IRT1 + FER1 transgenic plants 10 months after establishment from stem cuttings growing under confined-field-trial conditions at Isabela Research Station, University of Puerto Rico. — CFT was established using a randomized block design with 4 reps and 20 plants/line/rep. [file 41587_2018_2_Fig14_ESM.jpg]

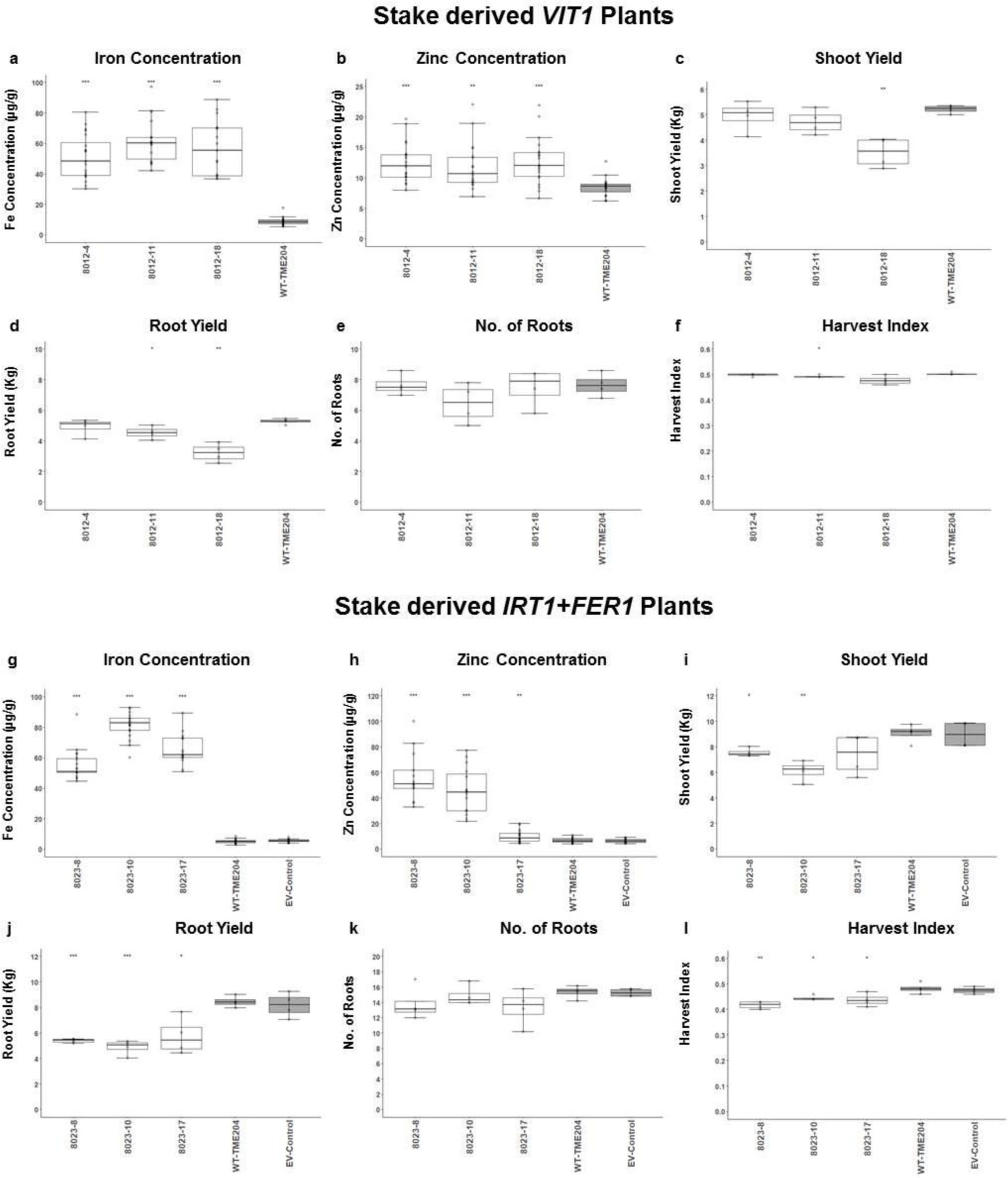

Supplement: Stake-derived VIT1, IRT1 + FER1 transgenic and nontransgenic control cassava plants grown under field conditions. — a) Iron, (b) Zinc concentration of storage roots (c) Shoot yield, (d) root yield, (e) number of roots and (f) harvest index from stake-derived VIT1 transgenic and non-transgenic control cassava plants grown under field conditions. (g) Iron, (h) Zinc concentration of storage roots (i) Shoot yield, (j) root yield, (k) number of roots and (l) harvest index from stake-derived IRT1 + FER1 transgenic and non-transgenic control cassava plants. Values are means of 20 biologically independent plants in 4 replicates (5 plants per replicate). Box and whisker plots were constructed using the R software package The upper whisker extends from the hinge to the largest value no further than 1.5* IQR from the hinge (where IQR is the inter-quartile range, or distance between the first and third quartiles). The lower whisker extends from the hinge to the smallest value at most 1.5* IQR of the hinge. Data beyond the end of the whiskers are called “outlying” points and are plotted individually. Statistical tests were two-sided using Student’s t-test compared to non-transgenic control. *, ** and *** represent significance levels at p ≤ 0.05, p ≤ 0.01 and p ≤ 0.001 respectively. [file 41587_2018_2_Fig15_ESM.jpg]

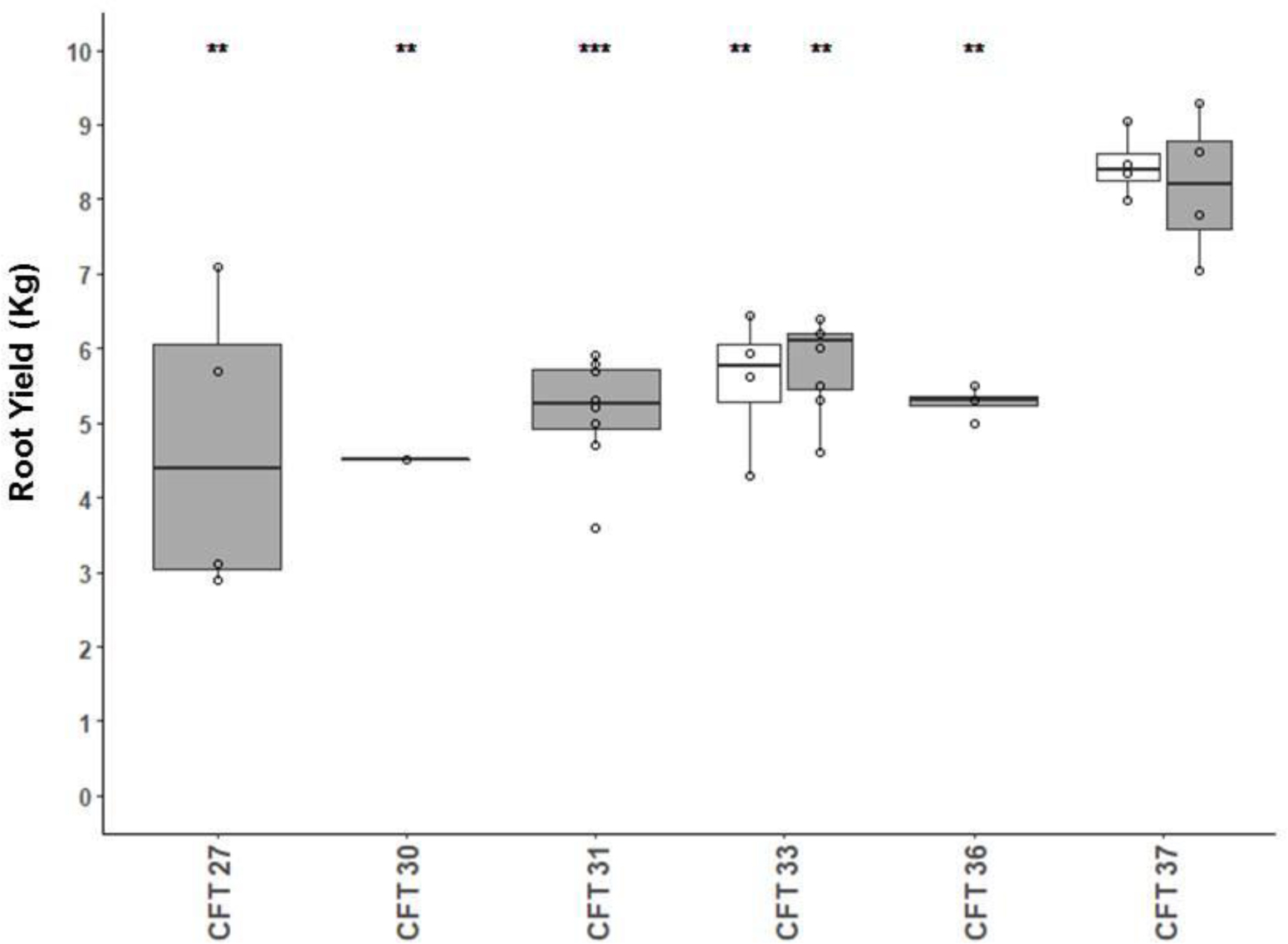

Supplement: Average storage root yields of wild-type TME 204 (gray bars) and empty-vector controls (white bars) under field conditions across different confined field trials, harvested 12 months after planting. — For CFT 27 and CFT 30, n = 3 biologically independent plants; CFT 31, n = 9 (3 plants per replicate); CFT33, n = 8; CFT 36 and 37, n = 20 (5 plants per replicate). Box and whisker plots were constructed using the R software package The upper whisker extends from the hinge to the largest value no further than 1.5* IQR from the hinge (where IQR is the inter-quartile range, or distance between the first and third quartiles). The lower whisker extends from the hinge to the smallest value at most 1.5* IQR of the hinge. Data beyond the end of the whiskers are called “outlying” points and are plotted individually. Statistical tests were two-sided using Student’s t-test compared to non-transgenic control. *, ** and *** represent significance levels at p ≤ 0.05, p ≤ 0.01 and p ≤ 0.001 respectively. [file 41587_2018_2_Fig16_ESM.jpg]

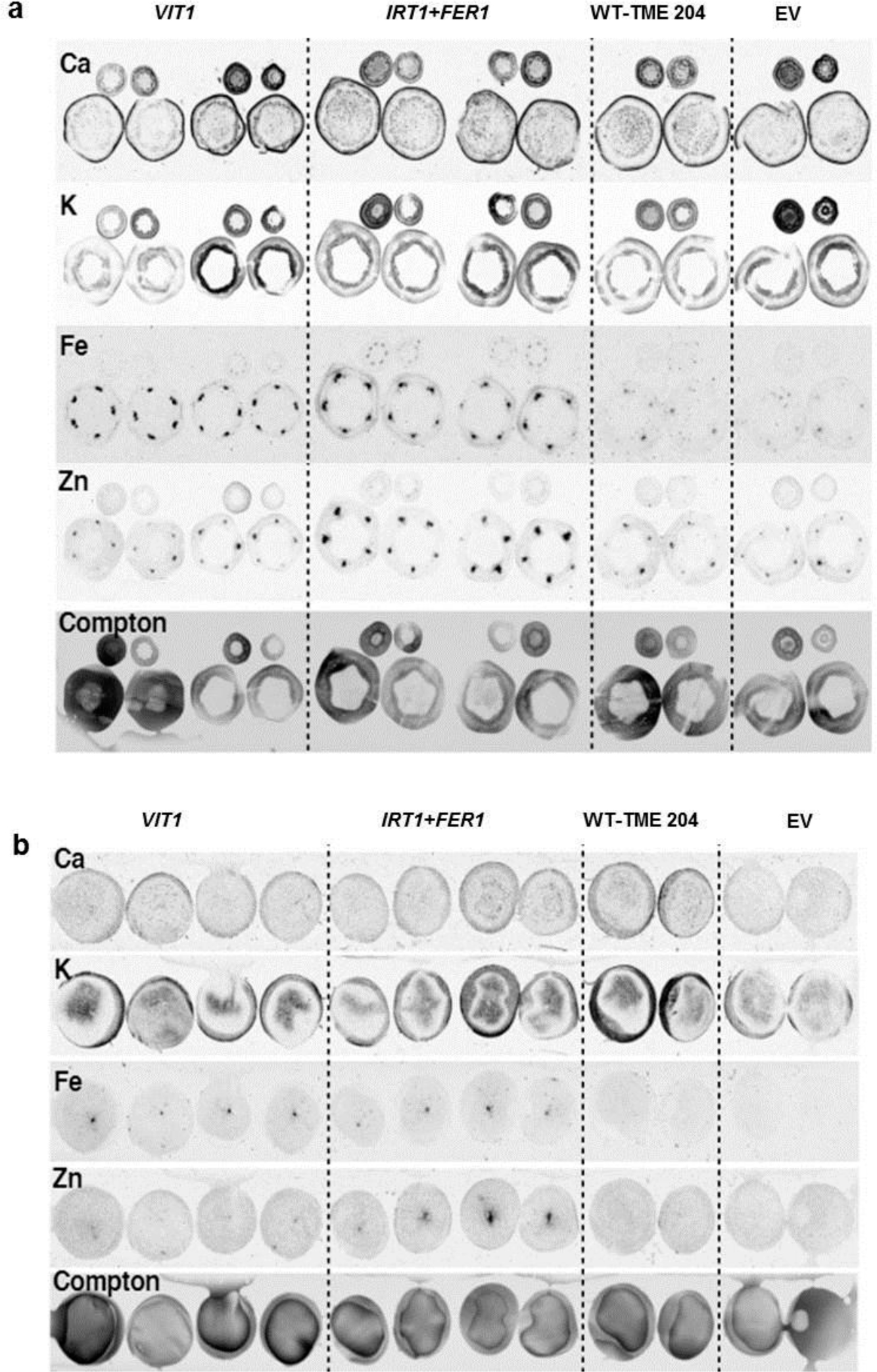

Supplement: Elemental and Compton scattering maps of VIT1 and IRT1 + FER1 transgenic cassava stems and petioles. — a) Elemental and Compton scattering maps obtained from a single scan (run 14761) of a series of transgenic, empty vector control (EV) and non-transgenic control cassava stems and petioles. Scan area was 96 mm × 15 mm with 0.02 mm pitch, and obtained with a collection time of 0.005 seconds/pixel for a total scan time of approximately 5 h. b) Elemental and Compton scattering maps obtained from a single scan (run 14762) of a series of transgenic, empty vector (EV) and wild type cassava storage roots. Scan area was 118 mm × 13 mm with 0.02 mm pitch, and obtained with a collection time of 0.006 seconds/pixel for a total scan time of approximately 7 h. The maximum intensity of each image has been scaled independently. [file 41587_2018_2_Fig17_ESM.jpg]

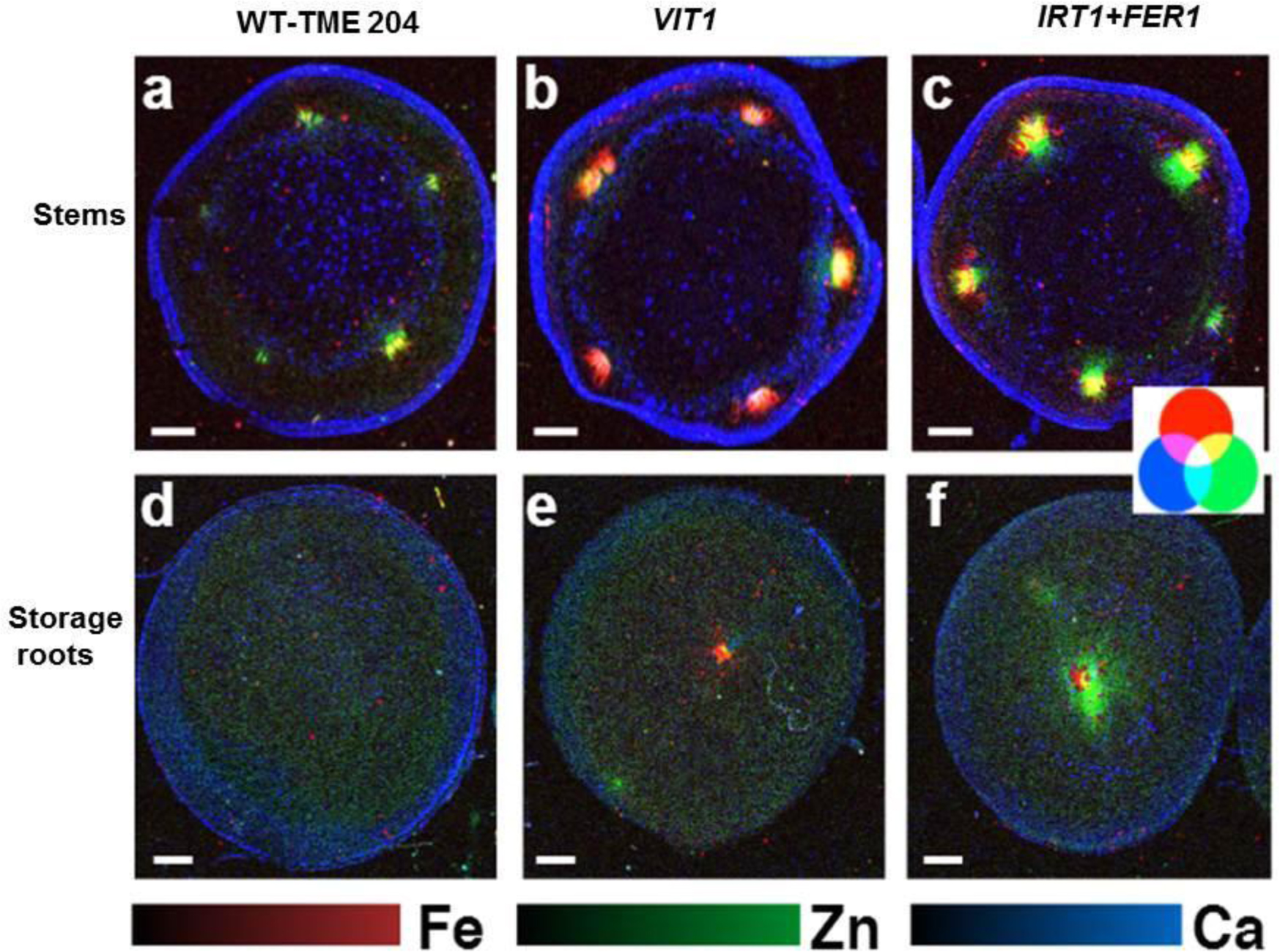

Supplement: Elemental mapping by synchrotron XRF of VIT1 and IRT1 + FER1 transgenic cassava stems and storage roots. — Composite, 2D elemental concentration maps of wet, as cut thin sections of (a, d) wild type, (b, e) VIT1 and (c, f) IRT1 + FER1 cassava (a-c) stems and (d-f) storage roots. Relative concentrations of iron, zinc and calcium are represented as the intensity of red, green and blue respectively. The brightness has been scaled separately for each element, but is identical for each sample type. Scale bars at the lower left correspond to 1 mm. The color guide at mid-right illustrates the appearance of overlapping colors. In particular, yellow regions in (c) indicate strong co-localization of Fe and Zn in vascular bundles in IRT1 + FER1 stems. [file 41587_2018_2_Fig18_ESM.jpg]

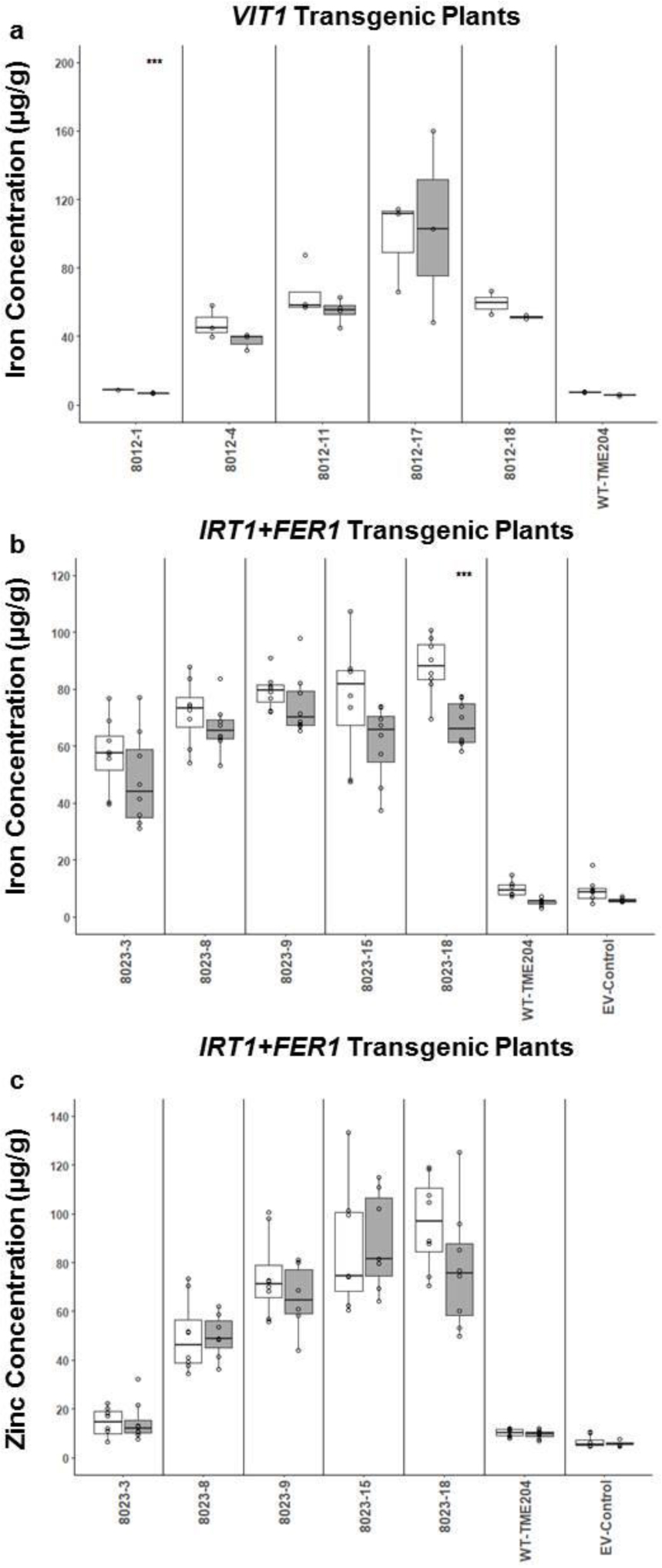

Supplement: Mineral retention of raw (white bars) and boiled (gray bars) VIT1 and IRT1 + FER1 storage roots. — Fe concentration after (a) boiling from storage roots of VIT1 transgenic cassava plants. (b) Fe and (c) Zn concentration after boiling from storage roots of IRT1 + FER1 transgenic cassava plants. For VIT1, n = 3 biologically independent plants and for IRT1 + FER1, n = 4 biologically independent plants (2 technical replicates/plant). Box and whisker plots were constructed using the R software package The upper whisker extends from the hinge to the largest value no further than 1.5* IQR from the hinge (where IQR is the inter-quartile range, or distance between the first and third quartiles). The lower whisker extends from the hinge to the smallest value at most 1.5* IQR of the hinge. Data beyond the end of the whiskers are called “outlying” points and are plotted individually. Statistical tests were two-sided using Student’s t-test compared to raw storage roots. *, ** and *** represent significance levels at p ≤ 0.05, p ≤ 0.01 and p ≤ 0.001 respectively. [file 41587_2018_2_Fig19_ESM.jpg]
