# Supplementary material for: Biofortification of field-grown cassava by engineering expression of an iron transporter and ferritin
Source: Nat Biotechnol. 2019 Jan 28;37(2):144–51. doi: 10.1038/s41587-018-0002-1 (PMC6784895; doi:10.1038/s41587-018-0002-1)
Supplement: Supplementary file 16 — Supplementary Figures 1–15 [file 41587_2018_2_MOESM1_ESM.pdf]

nature biotechnology

SUPPLEMENTARY INFORMATION

https://doi.org/10.1038/s41587-018-0002-1

In the format provided by the authors and unedited.

Biofortification of field-grown cassava by engineering expression of an iron transporter and ferritin

Narayanan Narayanan[1], Getu Beyene[1], Raj Deepika Chauhan[1], Eliana Gaitán-Solís[1], Jackson Gehan[1], Paula Butts[1], Dimuth Siritunga[2], Ihuoma Okwuonu[3], Arthur Woll[4], Dulce M. Jiménez-Aguilar[5], Erick Boy[6], Michael A. Grusak[7], Paul Anderson[1] and Nigel J. Taylor[1*]

[1]Donald Danforth Plant Science Center, St. Louis, MO, USA. [2]University of Puerto Rico Mayagüez, Puerto Rico, USA. [3]National Root Crops Research Institute, Umudike, Nigeria. [4]Cornell High Energy Synchrotron Source, Cornell University, Ithaca, NY, USA. [5]USDA-ARS Children's Nutrition Research Center, Baylor College of Medicine, Houston, TX, USA. [6]Harvest Plus/International Food Policy Research Institute, Washington, DC, USA. [7]USDA-ARS Edward T. Schafer Agricultural Research Center, Fargo, ND, USA. *e-mail: ntaylor@danforthcenter.org

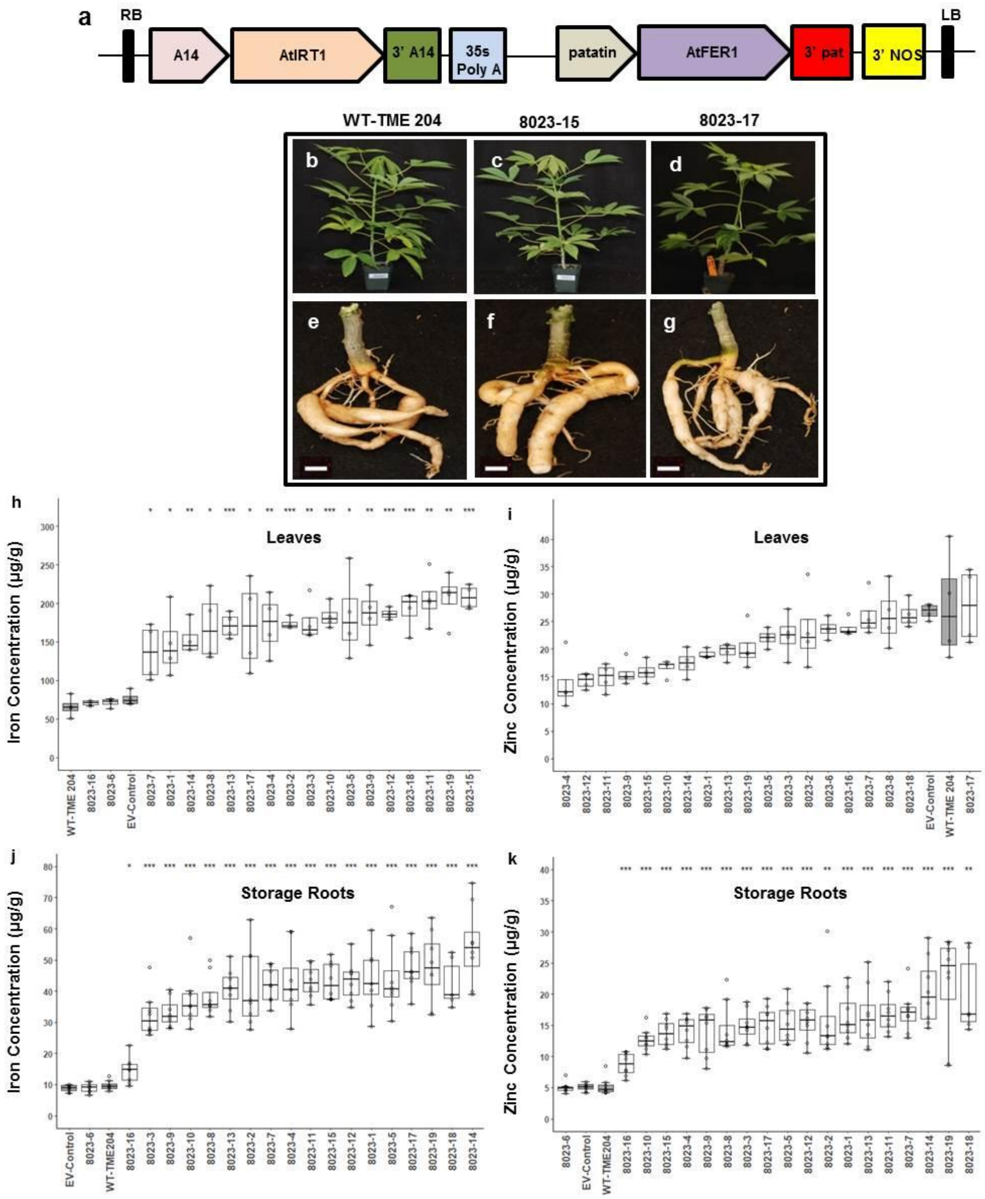

Supplementary Figure 1

Transgenic plants overexpressing *IRT1 + FER1*

(**a**) Schematic representation of the *IRT1+FER1* T-DNA construct for genetic transformation in cassava. RB and LB symbolizes the right and left borders of the T-DNA respectively, A14: epidermal promoter from *Arabidopsis*, *AtIRT1*: iron regulated transporter from *Arabidopsis*, 3' *A14*: 3'UTR from A14, 35s polyA: 3' UTR from Cauliflower mosaic virus, patatin: promoter from potato, *AtFER1*: ferritin storage protein from *Arabidopsis, 3' pat*: 3' UTR from patatin, 3'Nos: 3' UTR from *Agrobacterium*. (**b-d**) Phenotype (**e-g**) storage roots of 16-week old wildtype TME 204 and *IRT1+FER1* transgenic plants grown under greenhouse conditions. Scale bars at the lower left correspond to 1 cm. (**h**) Leaf iron (**i**) leaf zinc (**j**) storage root iron and (**k**) storage root zinc concentrations of *IRT1+FER1* transgenic plants. For *IRT1+FER1,* n= 4 biologically independent plants. Box and whisker plots were constructed using the R software package The upper whisker extends from the hinge to the largest value no further than 1.5* IQR from the hinge (where IQR is the inter-quartile range, or distance between the first and third quartiles). The lower whisker extends from the hinge to the smallest value at most 1.5* IQR of the hinge. Data beyond the end of the whiskers are called "outlying" points and are plotted individually. Statistical tests were two-sided using Student's t-test compared to non-transgenic control. *, ** and *** represent significance levels at $p \leq 0.05$, $p \leq 0.01$ and $p \leq 0.001$ respectively.

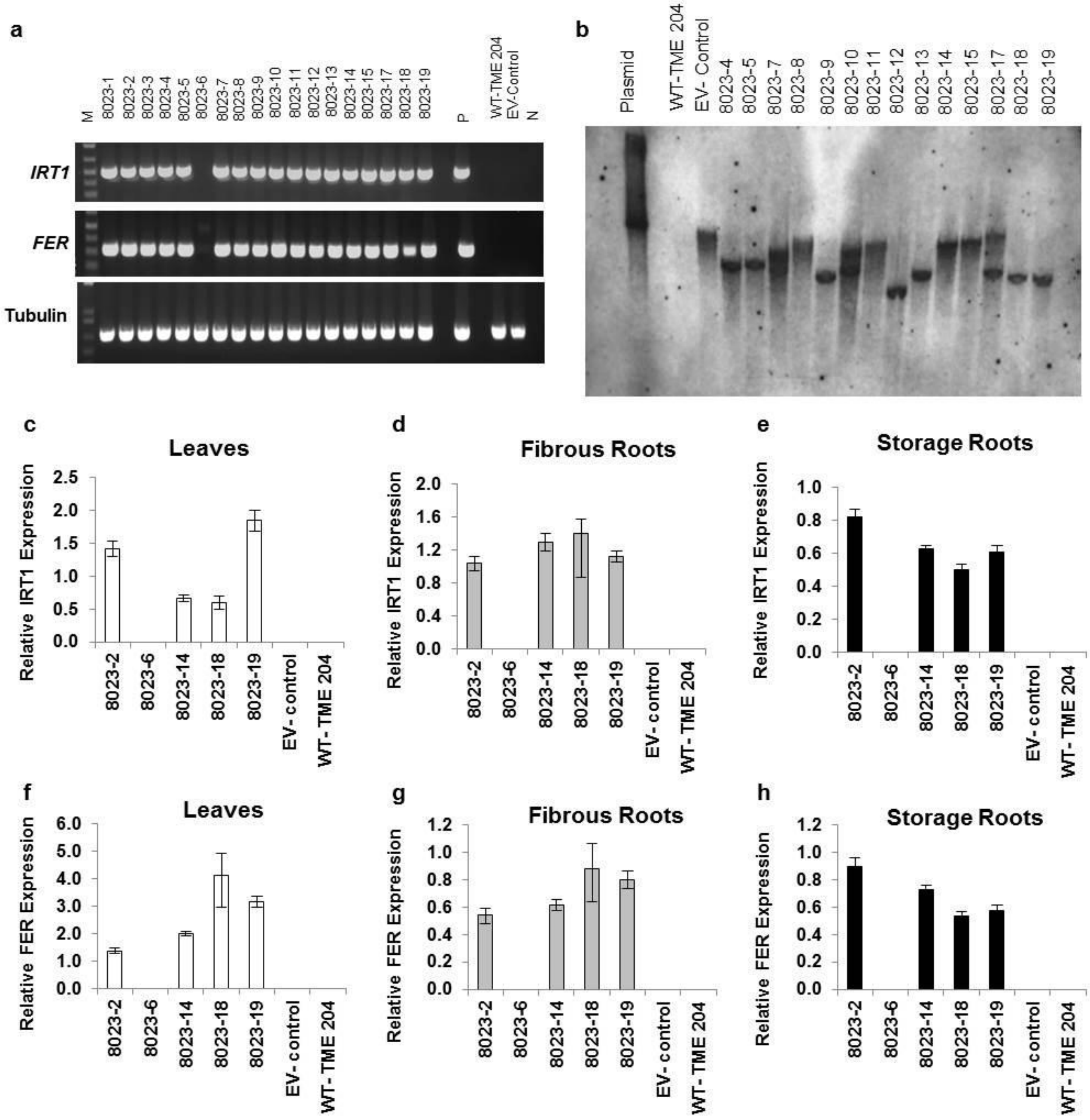

Supplementary Figure 2

Molecular characterization of *IRT1* + *FER1* transgenic plants

(a) PCR screening for presence of *IRT1* and *FER1* transgenes in leaves of 4-week old *in vitro* plants. Tubulin was used as a control. M- marker; WT- wildtype; EV- empty vector; N- negative water control; **(b)** Genomic DNA blot hybridization from wildtype and transgenic *IRT1+FER1* cassava leaves (6-week old) to determine T-DNA insertion number. Positive represents the plasmid DNA. Both gel and blots were cropped for better resolution. Quantitative expression of *IRT1* and *FER1* in **(c, f)** leaves, **(d, g)** fibrous roots and **(e, h)** storage roots of *IRT1+FER1* transgenic cassava plants. Tissues were collected from 16-week-old plants grown in the greenhouse. Expression was compared and normalized to protein phosphatase 2 (pp2A). Line 8023-2 expression values were adjusted to a value of 1 and all other expression values expressed relative to this line. Values are means of 4 biologically independent plants and error bars represent SD. This experiment was repeated 2 times independently with similar results.

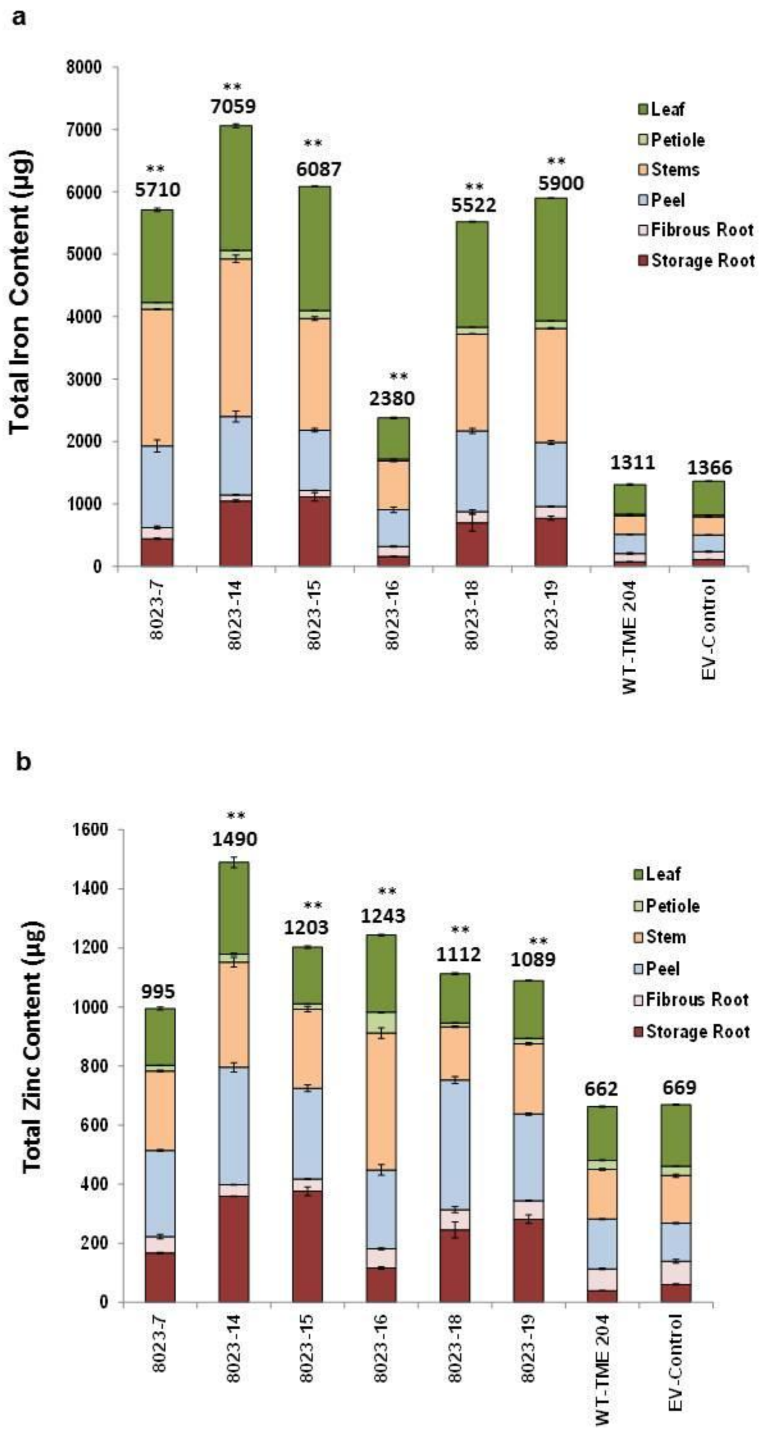

Supplementary Figure 3

Total iron and zinc content of *IRT1* + *FER1* transgenic cassava plants under greenhouse conditions

(a) Iron and (b) zinc. Tissues were collected from 16-week old cassava plants and subjected to ICP-OES elemental analysis. Values are means of four biological independent plants. Bars represent SD. *, and ** stands for significant difference, respectively, at $p \leq 0.05$, $p \leq 0.01$, using Student's t-test compared to wildtype. WT; wild type. EV control, empty vector control plants.

a

b

c

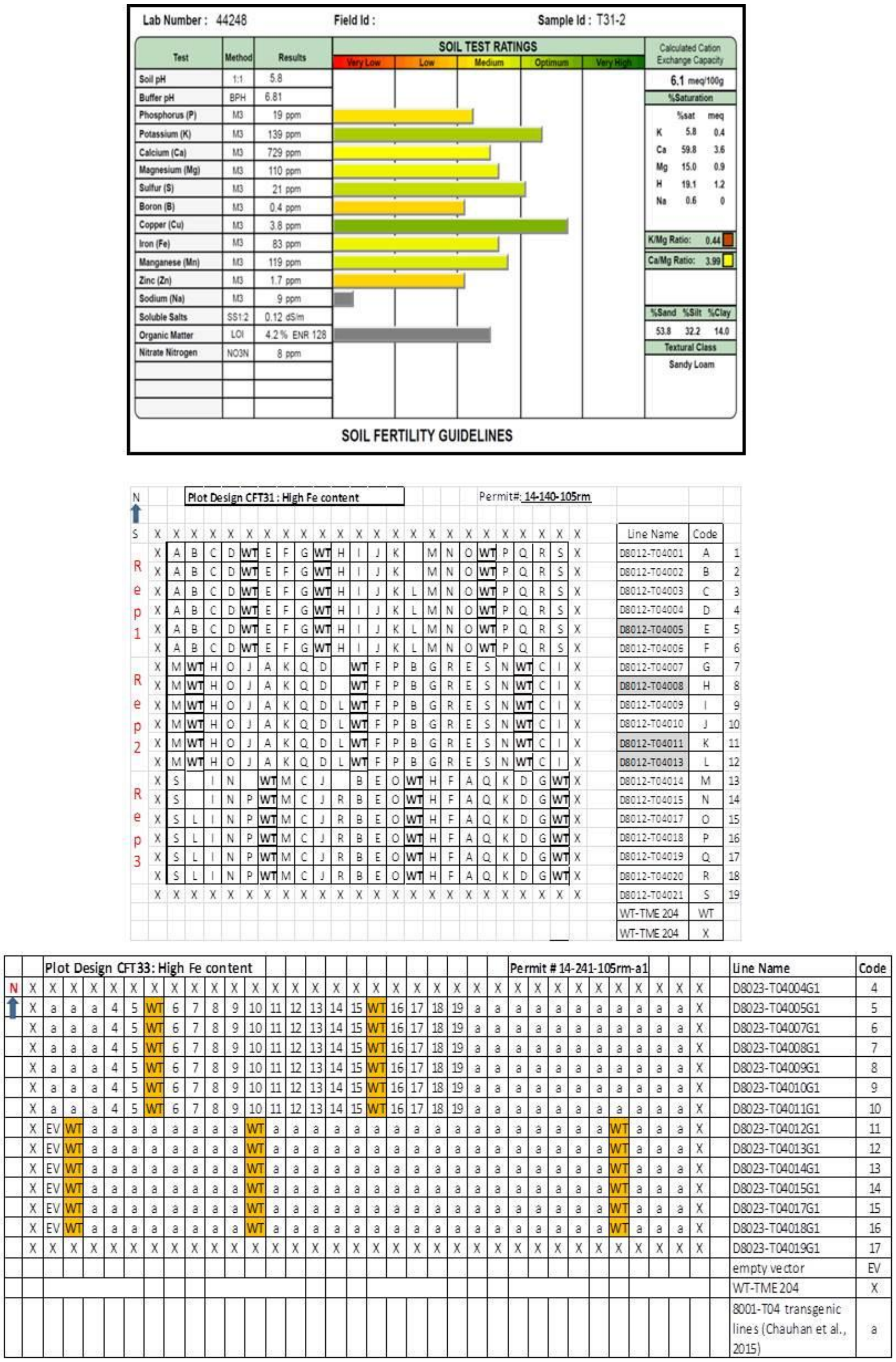

Test	Method	Results
Soil pH	1:1	5.8
Buffer pH	BPH	6.81
Phosphorus (P)	M3	19 ppm
Potassium (K)	M3	139 ppm
Calcium (Ca)	M3	729 ppm
Magnesium (Mg)	M3	110 ppm
Sulfur (S)	M3	21 ppm
Boron (B)	M3	0.4 ppm
Copper (Cu)	M3	3.8 ppm
Iron (Fe)	M3	83 ppm
Manganese (Mn)	M3	119 ppm
Zinc (Zn)	M3	1.7 ppm
Sodium (Na)	M3	9 ppm
Soluble Salts	SS1:2	0.12 dS/m
Organic Matter	LOI	4.2 % ENR 128
Nitrate Nitrogen	NO3N	8 ppm

Calculated Cation Exchange Capacity: 6.1 meq/100g

%Saturation	%sat	meq
K	5.8	0.4
Ca	59.8	3.6
Mg	15.0	0.9
H	19.1	1.2
Na	0.6	0

K/Mg Ratio: 0.44

Ca/Mg Ratio: 3.99

%Sand	%Silt	%Clay
53.8	32.2	14.0

Textural Class: Sandy Loam

N		Plot Design CFT31 : High Fe content																Permit#: 14-140-105rm									
↑																											
S	X	X	X	X	X	X	X	X	X	X	X	X	X	X	X	X	X	X	X	X	X	X	X	X	Line Name	Code	
	X	A	B	C	D	WT	E	F	G	WT	H	I	J	K		M	N	O	WT	P	Q	R	S	X	D8012-T04001	A	1
R	X	A	B	C	D	WT	E	F	G	WT	H	I	J	K		M	N	O	WT	P	Q	R	S	X	D8012-T04002	B	2
e	X	A	B	C	D	WT	E	F	G	WT	H	I	J	K	L	M	N	O	WT	P	Q	R	S	X	D8012-T04003	C	3
p	X	A	B	C	D	WT	E	F	G	WT	H	I	J	K	L	M	N	O	WT	P	Q	R	S	X	D8012-T04004	D	4
1	X	A	B	C	D	WT	E	F	G	WT	H	I	J	K	L	M	N	O	WT	P	Q	R	S	X	D8012-T04005	E	5
	X	A	B	C	D	WT	E	F	G	WT	H	I	J	K	L	M	N	O	WT	P	Q	R	S	X	D8012-T04006	F	6
	X	M	WT	H	O	J	A	K	Q	D		WT	F	P	B	G	R	E	S	N	WT	C	I	X	D8012-T04007	G	7
R	X	M	WT	H	O	J	A	K	Q	D		WT	F	P	B	G	R	E	S	N	WT	C	I	X	D8012-T04008	H	8
e	X	M	WT	H	O	J	A	K	Q	D	L	WT	F	P	B	G	R	E	S	N	WT	C	I	X	D8012-T04009	I	9
p	X	M	WT	H	O	J	A	K	Q	D	L	WT	F	P	B	G	R	E	S	N	WT	C	I	X	D8012-T04010	J	10
2	X	M	WT	H	O	J	A	K	Q	D	L	WT	F	P	B	G	R	E	S	N	WT	C	I	X	D8012-T04011	K	11
	X	M	WT	H	O	J	A	K	Q	D	L	WT	F	P	B	G	R	E	S	N	WT	C	I	X	D8012-T04013	L	12
	X	S		I	N		WT	M	C	J		B	E	O	WT	H	F	A	Q	K	D	G	WT	X	D8012-T04014	M	13
R	X	S		I	N	P	WT	M	C	J	R	B	E	O	WT	H	F	A	Q	K	D	G	WT	X	D8012-T04015	N	14
e	X	S	L	I	N	P	WT	M	C	J	R	B	E	O	WT	H	F	A	Q	K	D	G	WT	X	D8012-T04017	O	15
p	X	S	L	I	N	P	WT	M	C	J	R	B	E	O	WT	H	F	A	Q	K	D	G	WT	X	D8012-T04018	P	16
3	X	S	L	I	N	P	WT	M	C	J	R	B	E	O	WT	H	F	A	Q	K	D	G	WT	X	D8012-T04019	Q	17
	X	S	L	I	N	P	WT	M	C	J	R	B	E	O	WT	H	F	A	Q	K	D	G	WT	X	D8012-T04020	R	18
	X	X	X	X	X	X	X	X	X	X	X	X	X	X	X	X	X	X	X	X	X	X	X	X	D8012-T04021	S	19
																									WT-TME 204	WT	
																									WT-TME 204	X	

		Plot Design CFT33: High Fe content																							Permit # 14-241-105rm-a1											Line Name	Code
N	X	X	X	X	X	X	X	X	X	X	X	X	X	X	X	X	X	X	X	X	X	X	X	X	X	X	X	X	X	X	X	X	X	X		D8023-T04004G1	4
↑	X	a	a	a	4	5	WT	6	7	8	9	10	11	12	13	14	15	WT	16	17	18	19	a	a	a	a	a	a	a	a	a	a	a	X		D8023-T04005G1	5
	X	a	a	a	4	5	WT	6	7	8	9	10	11	12	13	14	15	WT	16	17	18	19	a	a	a	a	a	a	a	a	a	a	a	X		D8023-T04007G1	6
	X	a	a	a	4	5	WT	6	7	8	9	10	11	12	13	14	15	WT	16	17	18	19	a	a	a	a	a	a	a	a	a	a	a	X		D8023-T04008G1	7
	X	a	a	a	4	5	WT	6	7	8	9	10	11	12	13	14	15	WT	16	17	18	19	a	a	a	a	a	a	a	a	a	a	a	X		D8023-T04009G1	8
	X	a	a	a	4	5	WT	6	7	8	9	10	11	12	13	14	15	WT	16	17	18	19	a	a	a	a	a	a	a	a	a	a	a	X		D8023-T04010G1	9
	X	a	a	a	4	5	WT	6	7	8	9	10	11	12	13	14	15	WT	16	17	18	19	a	a	a	a	a	a	a	a	a	a	a	X		D8023-T04011G1	10
	X	EV	WT	a	a	a	a	a	a	a	a	WT	a	a	a	a	a	a	a	a	a	a	a	a	a	a	a	a	a	WT	a	a	a	X		D8023-T04012G1	11
	X	EV	WT	a	a	a	a	a	a	a	a	WT	a	a	a	a	a	a	a	a	a	a	a	a	a	a	a	a	a	WT	a	a	a	X		D8023-T04013G1	12
	X	EV	WT	a	a	a	a	a	a	a	a	WT	a	a	a	a	a	a	a	a	a	a	a	a	a	a	a	a	a	WT	a	a	a	X		D8023-T04014G1	13
	X	EV	WT	a	a	a	a	a	a	a	a	WT	a	a	a	a	a	a	a	a	a	a	a	a	a	a	a	a	a	WT	a	a	a	X		D8023-T04015G1	14
	X	EV	WT	a	a	a	a	a	a	a	a	WT	a	a	a	a	a	a	a	a	a	a	a	a	a	a	a	a	a	WT	a	a	a	X		D8023-T04017G1	15
	X	EV	WT	a	a	a	a	a	a	a	a	WT	a	a	a	a	a	a	a	a	a	a	a	a	a	a	a	a	a	WT	a	a	a	X		D8023-T04018G1	16
	X	X	X	X	X	X	X	X	X	X	X	X	X	X	X	X	X	X	X	X	X	X	X	X	X	X	X	X	X	X	X	X	X	X		D8023-T04019G1	17
																																				empty vector	EV
																																				WT-TME 204	X
																																				8001-T04 transgenic lines (Chauhan et al., 2015)	a

Supplementary Figure 4

Plot layout of *VIT1* and *IRT1* + *FER1* transgenic lines

a) Chemical properties of soil at Isabela field station, University of Puerto Rico, Mayaguez, PR, USA. **(b)** Plot layout of replicated field trial of *VIT1* transgenic lines in 2014. **(c)** Plot layout of *IRT1+FER1* transgenic lines in 2015. WT; wildtype, X; buffer row with wildtype plants.

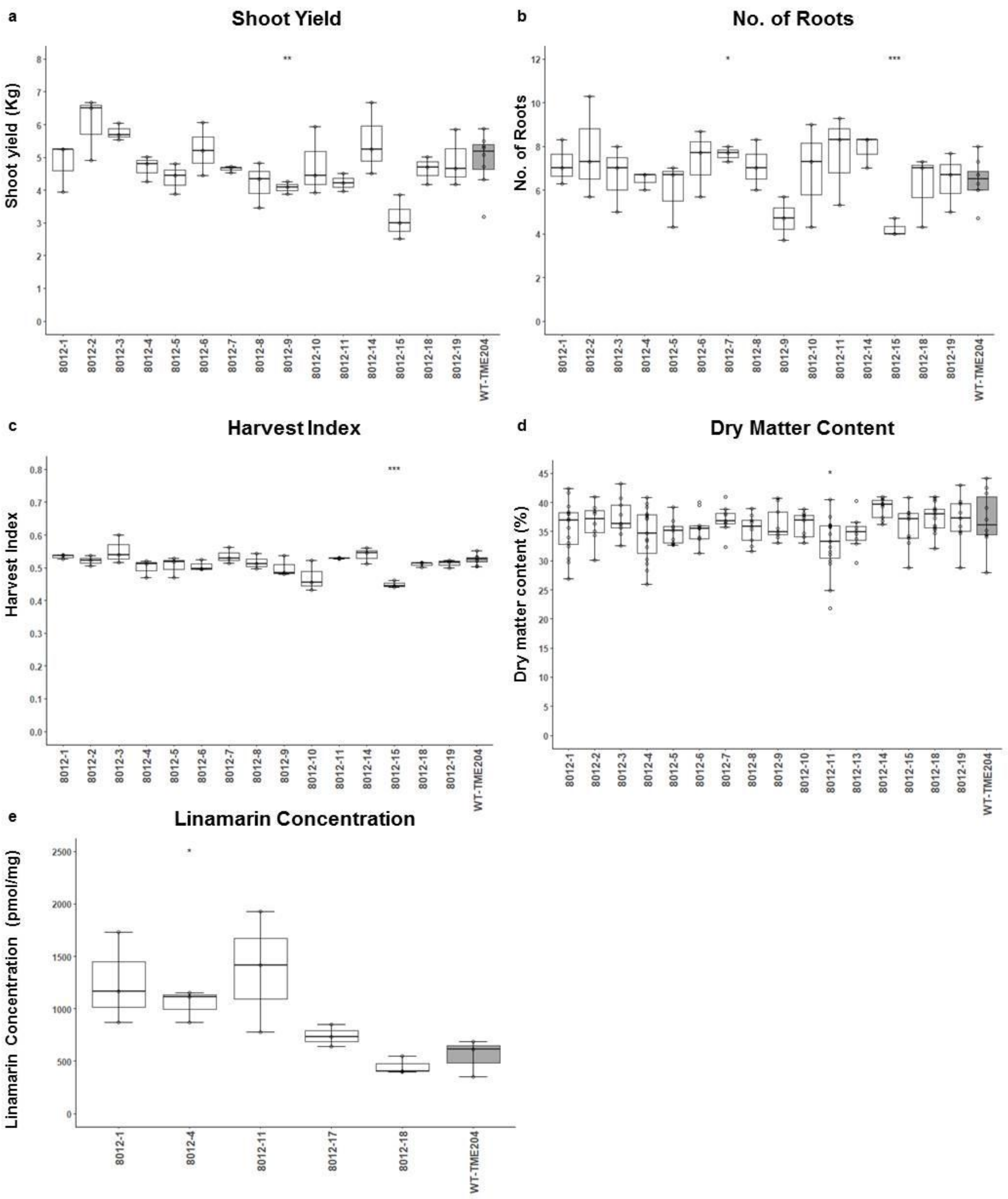

Supplementary Figure 5

Agronomic harvest data of *VIT1* transgenic plants

(a) Shoot yield, **(b)** number of roots, **(c)** harvest index, **(d)** dry matter content and **(e)** linamarin concentration of *VIT1* transgenic plants and non-transgenic control plants grown in confined field trial conditions. For *VIT1*, n= 9 biologically independent plants (3 plants/replicate). Box and whisker plots were constructed using the R software package The upper whisker extends from the hinge to the largest value no further than 1.5* IQR from the hinge (where IQR is the inter-quartile range, or distance between the first and third quartiles). The lower whisker extends from the hinge to the smallest value at most 1.5* IQR of the hinge. Data beyond the end of the whiskers are called "outlying" points and are plotted individually. Statistical tests were two-sided using Student's t-test compared to non-transgenic control. *, **, and *** represent significance levels at $p \leq 0.05$, $p \leq 0.01$ and $p \leq 0.001$ respectively.

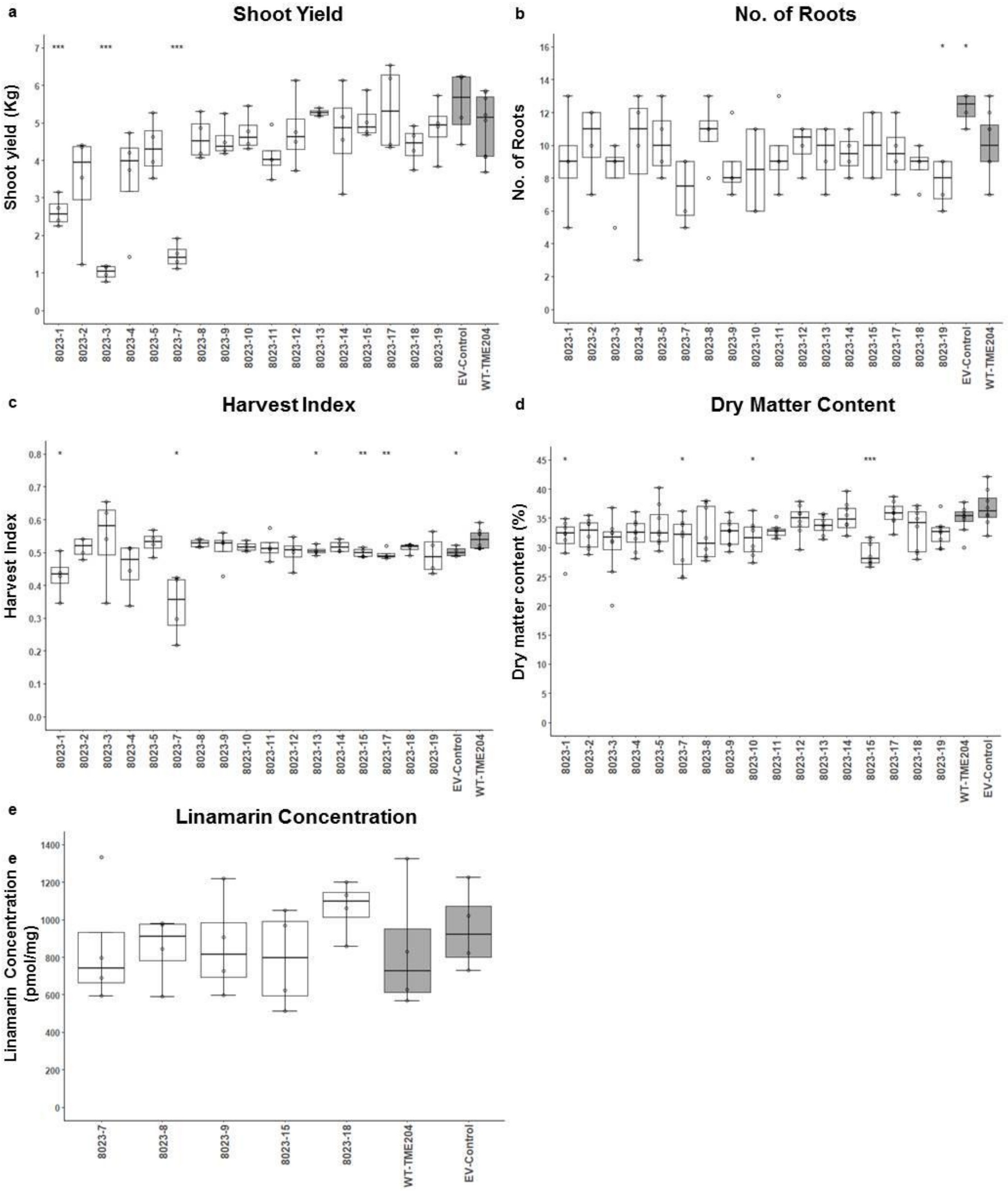

Supplementary Figure 6

Agronomic harvest data of *IRT1 + FER1* transgenic plants

(a) Shoot yield, **(b)** number of roots, **(c)** harvest index, **(d)** dry matter content and **(e)** linamarin concentration of *IRT1+FER1* transgenic plants and control plants grown in the confined field trial at University of Puerto Rico, Mayaguez. For *IRT1+FER1*, n= 4 biologically independent plants.). Box and whisker plots were constructed using the R software package The upper whisker extends from the hinge to the largest value no further than 1.5* IQR from the hinge (where IQR is the inter-quartile range, or distance between the first and third quartiles). The lower whisker extends from the hinge to the smallest value at most 1.5* IQR of the hinge. Data beyond the end of the whiskers are called "outlying" points and are plotted individually. Statistical tests were two-sided using Student's t-test compared to non-transgenic control. *, ** and *** represent significance levels at $p \leq 0.05$, $p \leq 0.01$ and $p \leq 0.001$ respectively.

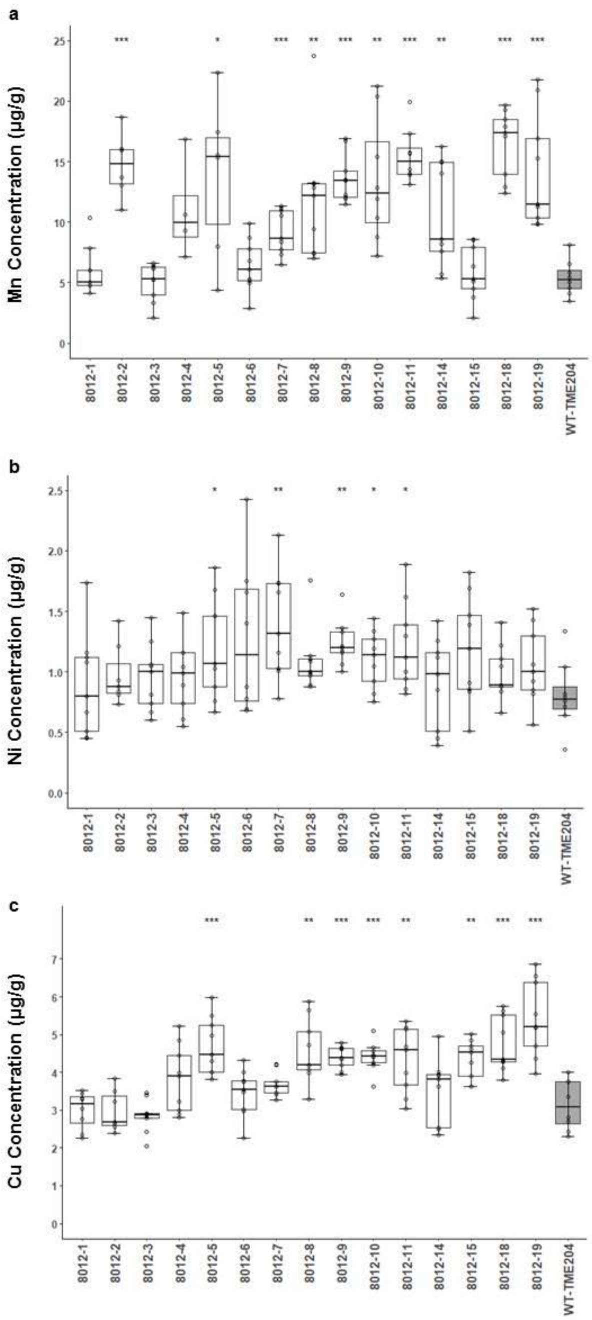

Supplementary Figure 7

Other mineral concentrations of storage roots of *VIT1* transgenic cassava plants under field conditions 12 months after planting

(a) Manganese, **(b)** Nickel, **(c)** Copper concentration. For *VIT1*, n= 9 biologically independent plants (3 plants/replicate).). Box and whisker plots were constructed using the R software package The upper whisker extends from the hinge to the largest value no further than 1.5* IQR from the hinge (where IQR is the inter-quartile range, or distance between the first and third quartiles). The lower whisker extends from the hinge to the smallest value at most 1.5* IQR of the hinge. Data beyond the end of the whiskers are called "outlying" points and are plotted individually. Statistical tests were two-sided using Student's t-test compared to non-transgenic control. *, **, and *** represent significance levels at $p \leq 0.05$, $p \leq 0.01$ and $p \leq 0.001$ respectively.

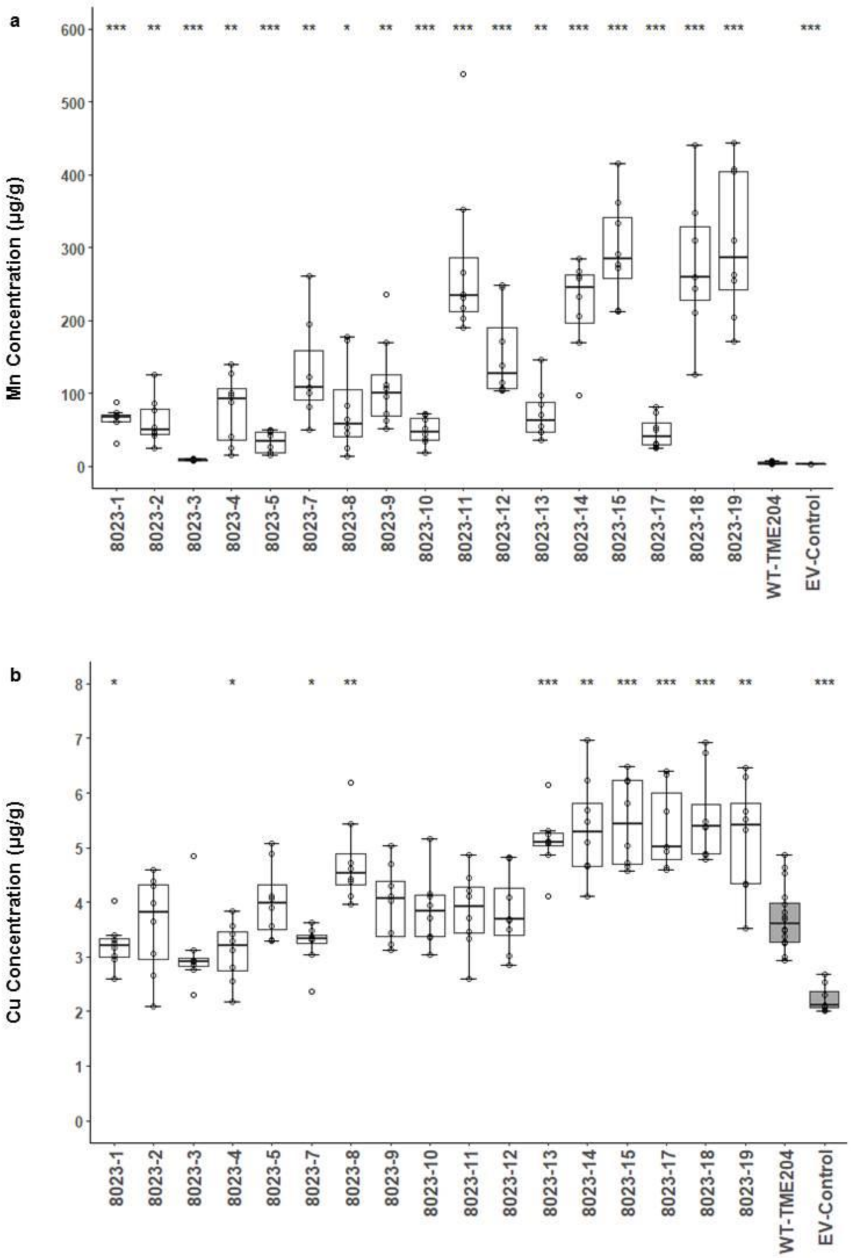

Supplementary Figure 8

Other mineral concentrations of storage roots of *IRT1* + *FER1* transgenic cassava plants under field conditions 12 months after planting

(a) Manganese and **(b)** **c**opper concentration. For *IRT1+FER1*, n= 4 biologically independent plants. Box and whisker plots were constructed using the R software package The upper whisker extends from the hinge to the largest value no further than 1.5* IQR from the hinge (where IQR is the inter-quartile range, or distance between the first and third quartiles). The lower whisker extends from the hinge to the smallest value at most 1.5* IQR of the hinge. Data beyond the end of the whiskers are called "outlying" points and are plotted individually. Statistical tests were two-sided using Student's t-test compared to non-transgenic control. *, ** and *** represent significance levels at $p \leq 0.05$, $p \leq 0.01$ and $p \leq 0.001$ respectively.

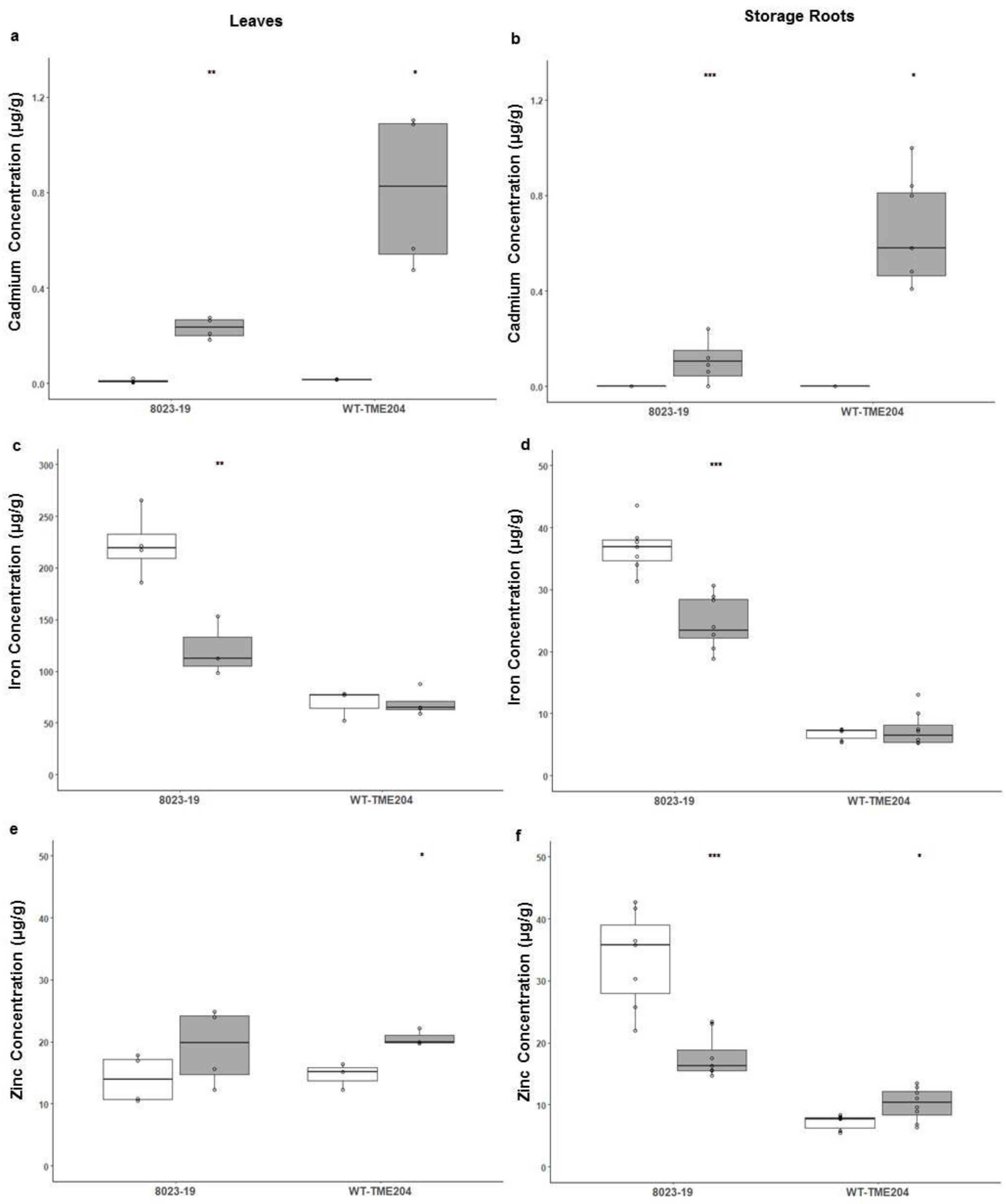

Supplementary Figure 9

Response of *IRT1 + FER1* transgenic plants to culture in high-cadmium conditions

Plants were treated with 10 µM $CdSO_4$ (grey bars) twice a week for with a 0 µM treatment (white bars). **(a)** Leaf cadmium, **(c)** iron and **(e)** zinc concentrations of WT and *IRT1+FER1* transgenic plants grown at low and high cadmium. Storage root **(b)** cadmium, **(d)** iron and **(f)** zinc concentrations of WT and *IRT1+FER1* transgenic plants grown at low and high cadmium. Values are means of 4 biologically independent plants. Box and whisker plots were constructed using the R software package The upper whisker extends from the hinge to the largest value no further than 1.5* IQR from the hinge (where IQR is the inter-quartile range, or distance between the first and third quartiles). The lower whisker extends from the hinge to the smallest value at most 1.5* IQR of the hinge. Data beyond the end of the whiskers are called "outlying" points and are plotted individually. Statistical tests were two-sided using Student's t-test compared to non-transgenic control. *, ** and *** represent significance levels at $p \leq 0.05$, $p \leq 0.01$ and $p \leq 0.001$ respectively.

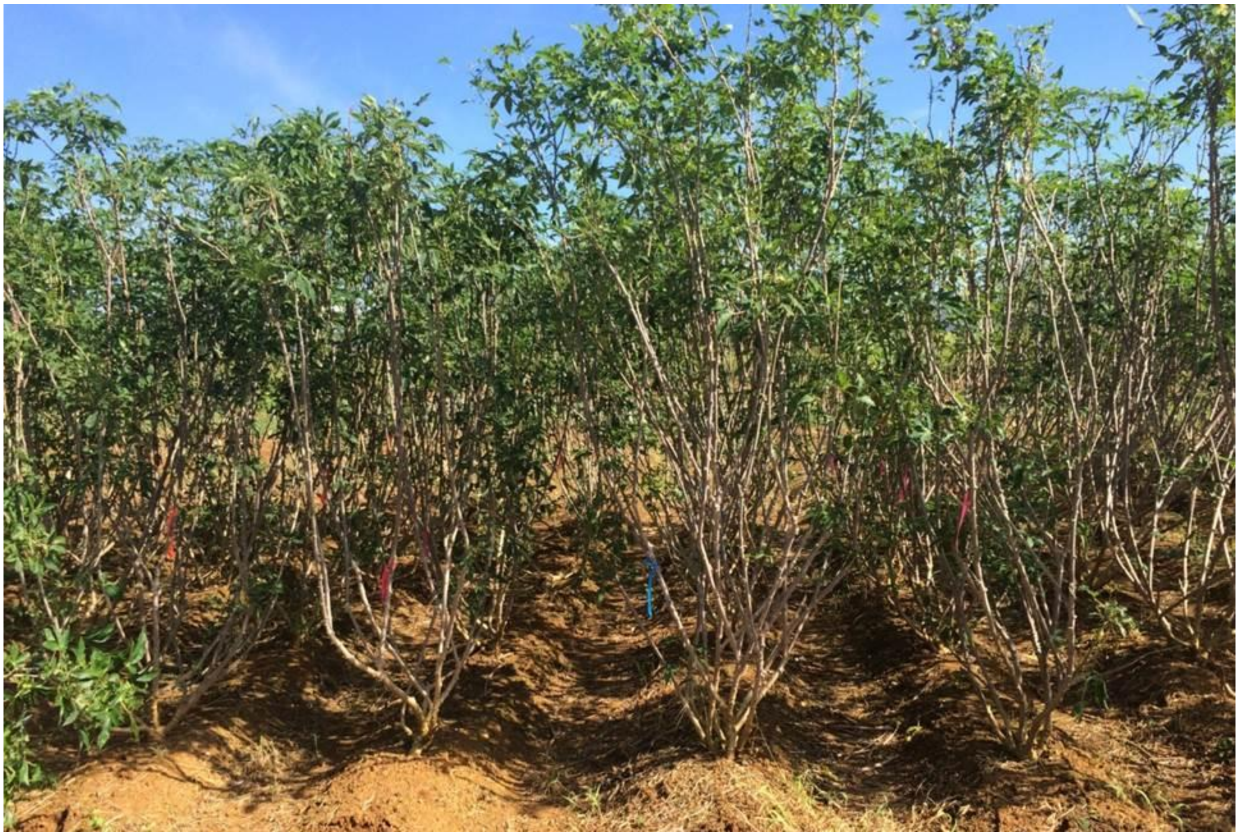

Supplementary Figure 10

IRT1 + FER1 transgenic plants 10 months after establishment from stem cuttings growing under confined-field-trial conditions at Isabela Research Station, University of Puerto Rico

CFT was established using a randomized block design with 4 reps and 20 plants/line/rep.

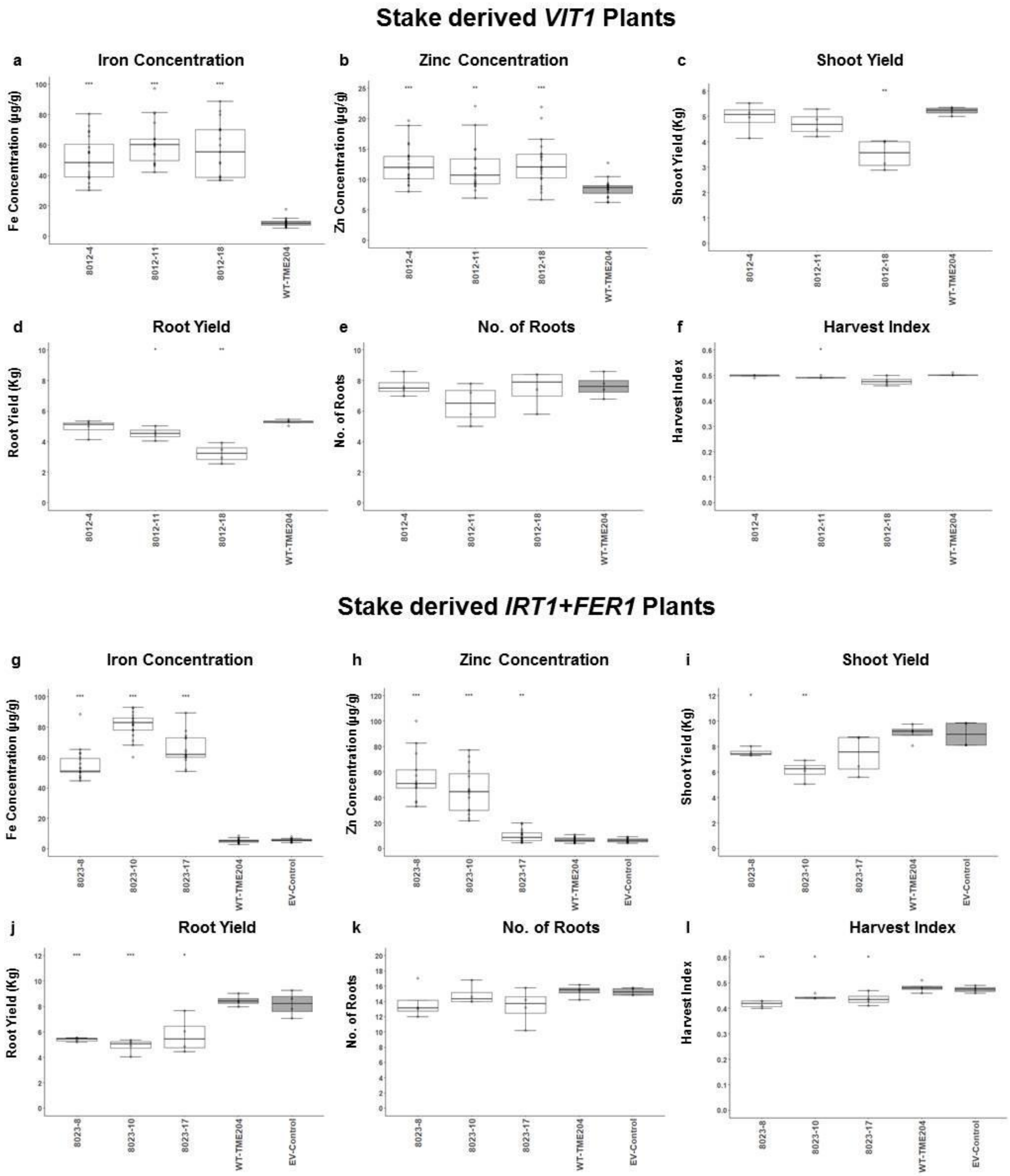

Supplementary Figure 11

Stake-derived *VIT1, IRT1 + FER1* transgenic and nontransgenic control cassava plants grown under field conditions

a) Iron, (**b**) Zinc concentration of storage roots **(c)** Shoot yield, **(d)** root yield, **(e)** number of roots and **(f)** harvest index from stake-derived *VIT1* transgenic and non-transgenic control cassava plants grown under field conditions. (**g**) Iron, (**h**) Zinc concentration of storage roots **(i)** Shoot yield, **(j)** root yield, **(k)** number of roots and **(l)** harvest index from stake-derived *IRT1+FER1* transgenic and non-transgenic control cassava plants. Values are means of 20 biologically independent plants in 4 replicates (5 plants per replicate). Box and whisker plots were constructed using the R software package The upper whisker extends from the hinge to the largest value no further than 1.5* IQR from the hinge (where IQR is the inter-quartile range, or distance between the first and third quartiles). The lower whisker extends from the hinge to the smallest value at most 1.5* IQR of the hinge. Data beyond the end of the whiskers are called "outlying" points and are plotted individually. Statistical tests were two-sided using Student's t-test compared to non-transgenic control. *, ** and *** represent significance levels at $p \leq 0.05$, $p \leq 0.01$ and $p \leq 0.001$ respectively.

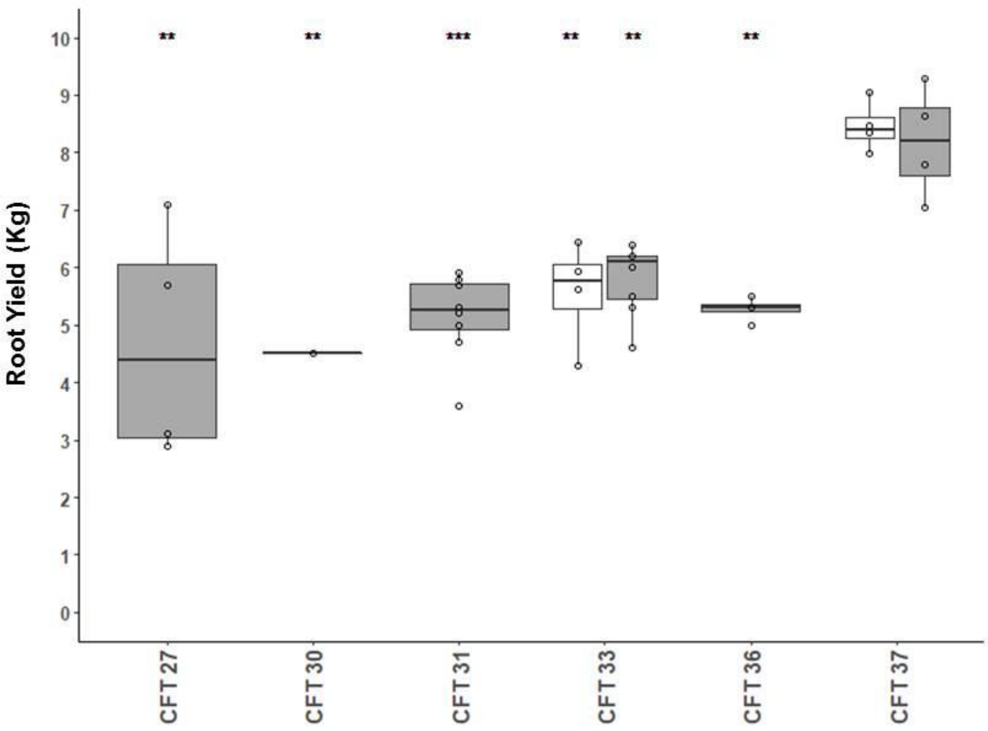

Supplementary Figure 12

Average storage root yields of wild-type TME 204 (gray bars) and empty-vector controls (white bars) under field conditions across different confined field trials, harvested 12 months after planting

For CFT 27 and CFT 30, n = 3 biologically independent plants; CFT 31, n =9 (3 plants per replicate); CFT33, n = 8; CFT 36 and 37, n =20 (5 plants per replicate). Box and whisker plots were constructed using the R software package The upper whisker extends from the hinge to the largest value no further than 1.5* IQR from the hinge (where IQR is the inter-quartile range, or distance between the first and third quartiles). The lower whisker extends from the hinge to the smallest value at most 1.5* IQR of the hinge. Data beyond the end of the whiskers are called "outlying" points and are plotted individually. Statistical tests were two-sided using Student's t-test compared to non-transgenic control. *, ** and *** represent significance levels at $p \leq 0.05$, $p \leq 0.01$ and $p \leq 0.001$ respectively.

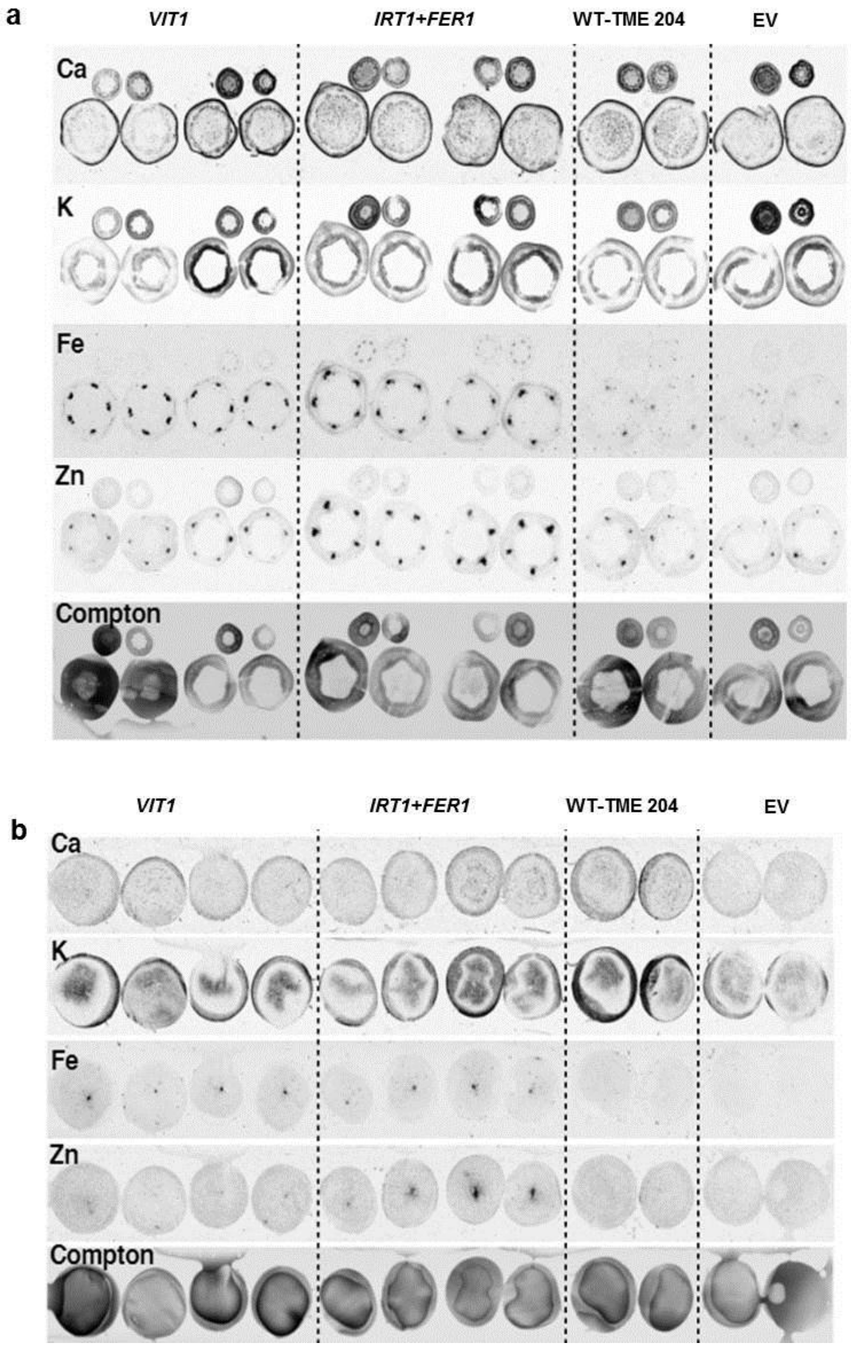

Supplementary Figure 13

Elemental and Compton scattering maps of *VIT1* and *IRT1 + FER1* transgenic cassava stems and petioles

a) Elemental and Compton scattering maps obtained from a single scan (run 14761) of a series of transgenic, empty vector control (EV) and non-transgenic control cassava stems and petioles. Scan area was 96 mm X 15 mm with 0.02 mm pitch, and obtained with a collection time of 0.005 seconds/pixel for a total scan time of approximately 5 hours. **b)** Elemental and Compton scattering maps obtained from a single scan (run 14762) of a series of transgenic, empty vector (EV) and wild type cassava storage roots. Sca n area was 118 mm X 13 mm with 0.02 mm pitch, and obtained with a collection time of 0.006 seconds/pixel for a total scan time of approximately 7 hours. The maximum intensity of each image has been scaled independently.

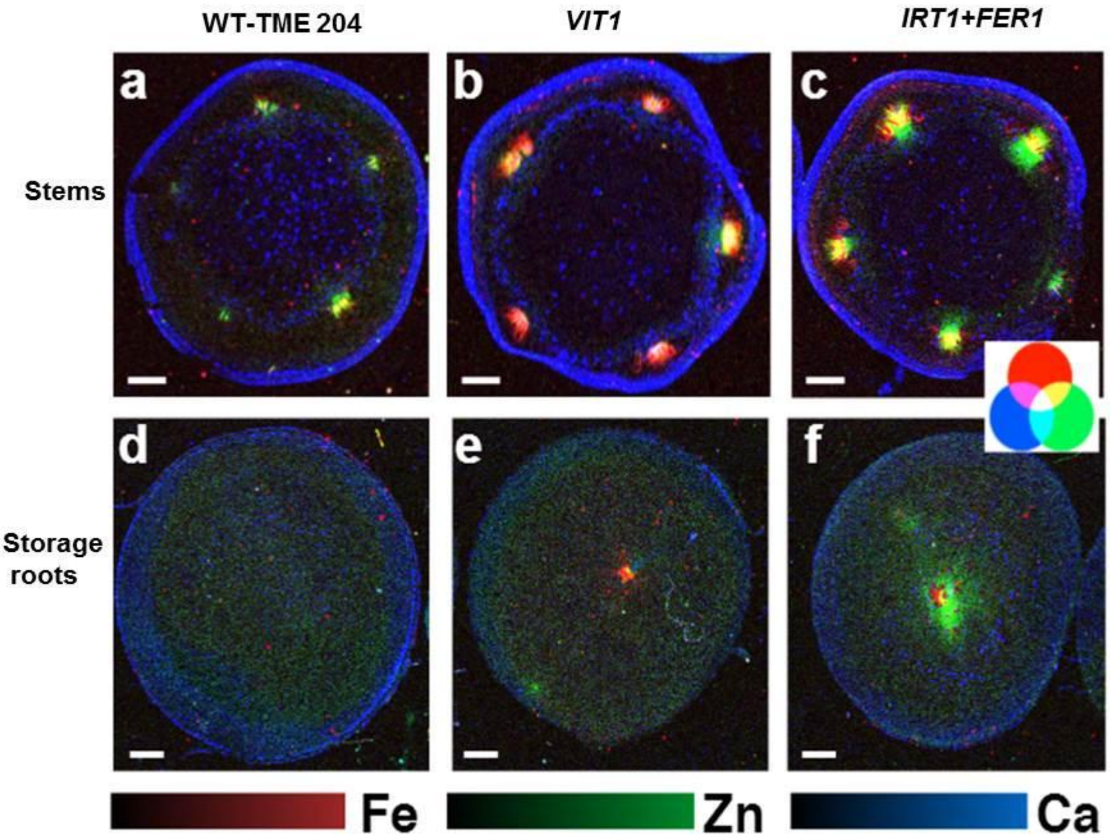

Supplementary Figure 14

Elemental mapping by synchrotron XRF of *VIT1* and *IRT1 + FER1* transgenic cassava stems and storage roots

Composite, 2D elemental concentration maps of wet, as cut thin sections of **(a, d)** wild type, **(b, e)** *VIT1* and **(c, f)** *IRT1+FER1* cassava **(a-c)** stems and **(d-f)** storage roots. Relative concentrations of iron, zinc and calcium are represented as the intensity of red, green and blue respectively. The brightness has been scaled separately for each element, but is identical for each sample type. Scale bars at the lower left correspond to 1 mm. The color guide at mid-right illustrates the appearance of overlapping colors. In particular, yellow regions in **(c)** indicate strong co-localization of Fe and Zn in vascular bundles in *IRT1+FER1* stems.

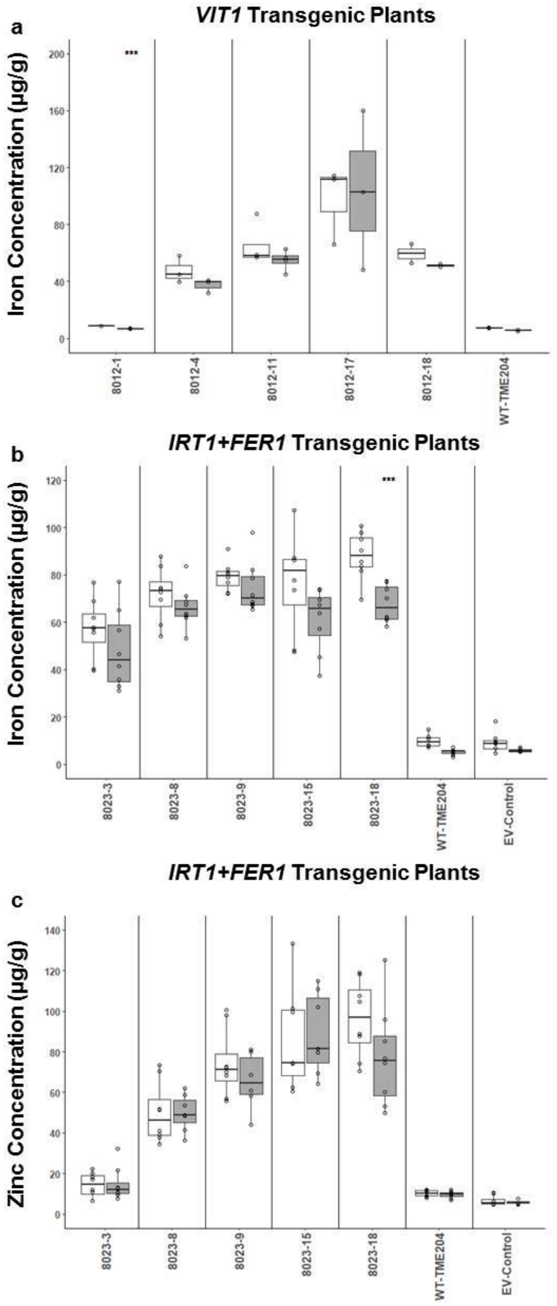

Supplementary Figure 15

Mineral retention of raw (white bars) and boiled (gray bars) *VIT1* and *IRT1 + FER1* storage roots

Fe concentration after **(a)** boiling from storage roots of *VIT1* transgenic cassava plants. **(b)** Fe and **(c)** Zn concentration after boiling from storage roots of *IRT1+FER1* transgenic cassava plants. For *VIT1,* n= 3 biologically independent plants and for *IRT1+FER1*, n= 4 biologically independent plants (2 technical replicates/plant). Box and whisker plots were constructed using the R software package The upper whisker extends from the hinge to the largest value no further than 1.5* IQR from the hinge (where IQR is the inter-quartile range, or distance between the first and third quartiles). The lower whisker extends from the hinge to the smallest value at most 1.5* IQR of the hinge. Data beyond the end of the whiskers are called "outlying" points and are plotted individually. Statistical tests were two-sided using Student's t-test compared to raw storage roots. *, ** and *** represent significance levels at $p \leq 0.05$, $p \leq 0.01$ and $p \leq 0.001$ respectively.
