# Supplementary material for: Biofortification of field-grown cassava by engineering expression of an iron transporter and ferritin
Source: Nat Biotechnol. 2019 Jan 28;37(2):144–51. doi: 10.1038/s41587-018-0002-1 (PMC6784895; doi:10.1038/s41587-018-0002-1)
Supplement: Supplementary file 17 — Reporting Summary [file 41587_2018_2_MOESM2_ESM.pdf]

nature research

Corresponding author(s): Nigel Taylor

Reporting Summary

Nature Research wishes to improve the reproducibility of the work that we publish. This form provides structure for consistency and transparency in reporting. For further information on Nature Research policies, see Authors & Referees and the Editorial Policy Checklist.

Statistical parameters

When statistical analyses are reported, confirm that the following items are present in the relevant location (e.g. figure legend, table legend, main text, or Methods section).

n/a	Confirmed	
☐	☒	The exact sample size (*n*) for each experimental group/condition, given as a discrete number and unit of measurement
☐	☒	An indication of whether measurements were taken from distinct samples or whether the same sample was measured repeatedly
☐	☒	The statistical test(s) used AND whether they are one- or two-sided<br>*Only common tests should be described solely by name; describe more complex techniques in the Methods section.*
☒	☐	A description of all covariates tested
☒	☐	A description of any assumptions or corrections, such as tests of normality and adjustment for multiple comparisons
☒	☐	A full description of the statistics including central tendency (e.g. means) or other basic estimates (e.g. regression coefficient) AND variation (e.g. standard deviation) or associated estimates of uncertainty (e.g. confidence intervals)
☐	☒	For null hypothesis testing, the test statistic (e.g. *F*, *t*, *r*) with confidence intervals, effect sizes, degrees of freedom and *P* value noted<br>*Give P values as exact values whenever suitable.*
☒	☐	For Bayesian analysis, information on the choice of priors and Markov chain Monte Carlo settings
☒	☐	For hierarchical and complex designs, identification of the appropriate level for tests and full reporting of outcomes
☒	☐	Estimates of effect sizes (e.g. Cohen's *d*, Pearson's *r*), indicating how they were calculated
☐	☒	Clearly defined error bars<br>*State explicitly what error bars represent (e.g. SD, SE, CI)*

Our web collection on statistics for biologists may be useful.

Software and code

Policy information about availability of computer code

Data collection	R software, version 3.4.0 and GeoPIXE v7.1 for X-ray fluorescence (XRF)
Data analysis	Construction of graphics and statistical analysis were performed using R software, version 3.4.0. Box and whisker plots were constructed using the R package “ggplot2”. To generate p-values as indicated in the graphics, a two-sample t-test was performed comparing the wild-type to the transgenic lines unless otherwise stated. X-ray fluorescence (XRF) data was analyzed by the dynamic analysis (DA) method to obtain elemental maps using the software GeoPIXE v7.1. The incident flux was calibrated using reference films of known weight concentration, used to calculate elemental weight concentrations from XRF peak areas via a fundamental-parameters approach.

For manuscripts utilizing custom algorithms or software that are central to the research but not yet described in published literature, software must be made available to editors/reviewers upon request. We strongly encourage code deposition in a community repository (e.g. GitHub). See the Nature Research guidelines for submitting code & software for further information.

Data

Policy information about availability of data

All manuscripts must include a data availability statement. This statement should provide the following information, where applicable:

- Accession codes, unique identifiers, or web links for publicly available datasets
- A list of figures that have associated raw data
- A description of any restrictions on data availability

The datasets generated and/or analyzed during the current study are available from the corresponding author on reasonable request. All data generated or analysed during this study are included in this published article and its supplementary information files.

Field-specific reporting

Please select the best fit for your research. If you are not sure, read the appropriate sections before making your selection.

☒ Life sciences ☐ Behavioural & social sciences ☐ Ecological, evolutionary & environmental sciences

For a reference copy of the document with all sections, see nature.com/authors/policies/ReportingSummary-flat.pdf

Life sciences study design

All studies must disclose on these points even when the disclosure is negative.

Sample size	For all the greenhouse and field experiments, sample size were determined with statistical rigor
Data exclusions	For VIT1 transgenic plants, one transgenic event was excluded due to poor growth performance in the field and had very small amount of roots.
Replication	All experiments in the greenhouse and field were replicated. They all contained statistically sufficient both biological and technical replicates.
Randomization	Whenever possible in the field experiments, plants were tested in a randomized replicated trial. In other experiments, plants were allocated in a random way to minimize edge effects.
Blinding	All the mineral measurements (ICP) were done by a in-house/third party lab and they were not aware of the sample name and number. During processing and collecting data in the greenhouse, technicians also were not aware of the sample name and number.

Reporting for specific materials, systems and methods

Materials & experimental systems

n/a	Involved in the study
☒	☐ Unique biological materials
☒	☐ Antibodies
☐	☒ Eukaryotic cell lines
☒	☐ Palaeontology
☒	☐ Animals and other organisms
☒	☐ Human research participants

Methods

n/a	Involved in the study
☒	☐ ChIP-seq
☒	☐ Flow cytometry
☒	☐ MRI-based neuroimaging

Eukaryotic cell lines

Policy information about cell lines

Cell line source(s)	n/a
Authentication	n/a
Mycoplasma contamination	n/a
Commonly misidentified lines (See ICLAC register)	n/a
